# Emerging Theranostic Nanomaterials in Diabetes and Its Complications

**DOI:** 10.1002/advs.202102466

**Published:** 2021-11-25

**Authors:** Yuntao Liu, Siqi Zeng, Wei Ji, Huan Yao, Lin Lin, Haiying Cui, Hélder A. Santos, Guoqing Pan

**Affiliations:** ^1^ School of Food & Biological Engineering Jiangsu University Zhenjiang 212013 China; ^2^ College of Food Science Sichuan Agricultural University Yaan 625014 China; ^3^ Department of Pharmaceutics School of Pharmacy Jiangsu University Zhenjiang Jiangsu 212013 China; ^4^ Sichuan Institute of Food Inspection Chengdu 610097 China; ^5^ Drug Research Program Division of Pharmaceutical Chemistry and Technology Faculty of Pharmacy University of Helsinki Helsinki FI‐00014 Finland; ^6^ Department of Biomedical Engineering and W.J. Kolff Institute for Biomedical Engineering and Materials Science University of Groningen/University Medical Center Groningen Ant. Deusinglaan 1 Groningen 9713 AV The Netherlands; ^7^ Institute for Advanced Materials School of Materials Science and Engineering Jiangsu University Zhenjiang Jiangsu 212013 China

**Keywords:** acetone sensing, antioxidant, diabetic complications, glucose detection, nanocarrier, wound healing

## Abstract

Diabetes mellitus (DM) refers to a group of metabolic disorders that are characterized by hyperglycemia. Oral subcutaneously administered antidiabetic drugs such as insulin, glipalamide, and metformin can temporarily balance blood sugar levels, however, long‐term administration of these therapies is associated with undesirable side effects on the kidney and liver. In addition, due to overproduction of reactive oxygen species and hyperglycemia‐induced macrovascular system damage, diabetics have an increased risk of complications. Fortunately, recent advances in nanomaterials have provided new opportunities for diabetes therapy and diagnosis. This review provides a panoramic overview of the current nanomaterials for the detection of diabetic biomarkers and diabetes treatment. Apart from diabetic sensing mechanisms and antidiabetic activities, the applications of these bioengineered nanoparticles for preventing several diabetic complications are elucidated. This review provides an overall perspective in this field, including current challenges and future trends, which may be helpful in informing the development of novel nanomaterials with new functions and properties for diabetes diagnosis and therapy.

## Graphical abstract

Nanomaterials have been widely used in various areas of medicine due to their mutable structures and extraordinary properties. This review paper comprehensively discusses the recent studies about the mechanisms and applications of functional nanomaterials in diabetes‐related diagnosis, prevention and therapy.

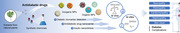

## 1 Introduction

### 1.1 Diabetes and Its Complications

**Figure 1:**
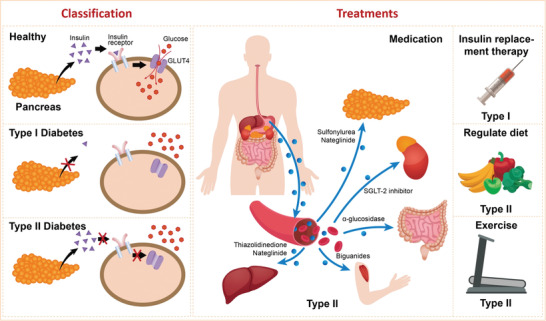
Diabetes classifications and approaches for management. The circle shows the various types of diabetes drugs that act on different tissues. GLUT4, glucose transporter 4; SGLT‐2, sodium‐dependent glucose transporters‐2.

Diabetes mellitus (DM) refers to a group of metabolic disorders that are characterized by a deficiency in insulin secretion, impaired insulin action, or a combination of both, resulting in hyperglycemia.^[^ ^1^, ^2^ ^]^ The main DM subtypes are Types I and II, which are grouped on the basis of their pathogenesis, such as insulin deficiency (Type I) and/or resistance (Type II). Moreover, there are other types of diabetes, such as gestational diabetes and neonatal diabetes (**Figure** **1**). The rapid increase in global diabetes prevalence has been attributed to rapid urbanization and significant changes towards sedentary lifestyles as well as unhealthy eating habits. The International Diabetes Federation (IDF) estimates that there were 451 million people worldwide with diabetes in 2017, and predicts that this number will have increased to 693 million people by the year 2045.^[^ ^3^ ^]^

Diabetes‐associated complications are the main causes of patient death compared to diabetes itself. Sustained hyperglycemia causes generalized vascular damage that can lead to renal failure, cardiovascular disease, ketoacidosis, stroke, nerve damage and retinopathy, as well as lower limb amputation.^[^ ^4^ ^]^ A specific diabetes type can lead to microvascular and macrovascular complications. When they are associated with other comorbidities like dyslipidemia and hypertension, these complications, such as nephropathy, retinopathy, neuropathy, and cardiovascular disease events vary according to the degree and duration of poorly controlled diabetes.^[^ ^5^ ^]^

### 1.2 Conventional Therapies for Diabetes

With the increase in the number of diabetes patients, diabetes management and treatment have become a major challenge to researchers and healthcare personnel. However, due to varying pathogenesis, different types of diabetes are treated differently (Figure 1). Since Type I is primarily associated with insulin deficiency, insulin administration through daily injections or an insulin pump is the main therapeutic option. Relatively, treatment of Type II diabetes is much easier as diet and exercise may be appropriate treatments, especially in the early stages. Patients can also be prescribed with some medications as auxiliary treatments. With rapid advances in drug discovery, new drugs such as SGLT‐2 (sodium‐dependent glucose transporters) and *α*‐glucosidase inhibitors are commercially available along with conventional drugs such as insulin, sulfonylureas, biguanides, and thiazolidinediones.^[^ ^6^ ^]^ However, the increase in the number of diabetes patients, as predicted by IDF, underscores the fact that current therapeutic approaches for diabetes are not effective.^[^ ^7^ ^]^ Therefore, there is an urgent need to develop new drugs as well as effective drug delivery strategies with more precise efficacies and fewer side effects. In this context, nanomedicine has emerged, and it has offered new hope in biomedicine due to its potential versatility, accuracy, and intelligence of the well‐designed nanomaterials.

### 1.3 Nanomaterials in Diabetes

**Figure 2:**
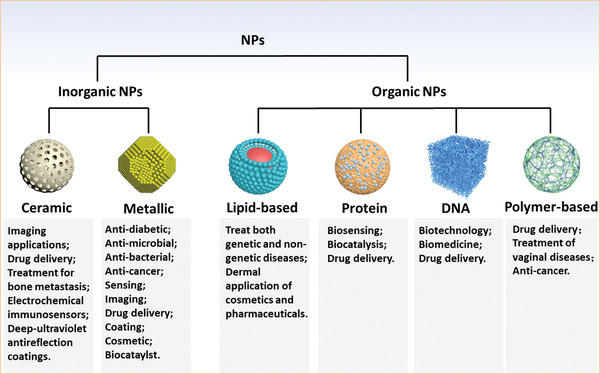
Classification and biomedical applications of nanoparticles (NPs).

Based on their chemical composition, nanoparticles can be divided into inorganic or organic nanoparticles.^[^ ^8^, ^9^, ^10^ ^]^ Due to their unique structures, these nanoparticles have several important characteristics, and thus, have various applications in the biomedical field (**Figure** **2**).

Inorganic nanoparticles are a class of materials without carbon‐hydrogen bonds in their chemical structures. They can be divided into ceramic (e.g., metallic oxide, metalloid, nonmetal and their oxides, nitrides, and carbides) and metallic nanoparticles. Quantum dots, an emerging group of ultrasmall semiconductor or carbon nanomaterials, are also considered as nanoceramics. Advances in the development of functional inorganic nanomaterials have enhanced their applications in chemical industries, energy, environment, and medicine.^[^ ^9^ ^]^ Organic nanoparticles are nanosized materials that are based on organic compounds derived from natural life or made by chemical synthesis. Biogenic macromolecules (such as lipids, proteins, and nucleic acids), synthetic polymers, and their composites have been used to fabricate nanomaterials, particularly, for biomedical applications. Synthetic polymer nanoparticles are a group of nanomaterials that are composed of man‐made polymers. These nanomaterials play significant roles in our daily lives, from energy, environmental biomedicine, and even art (e.g., paintings).^[^ ^11^, ^12^ ^]^ Synthetic polymer nanoparticles occur in various forms, such as solid spheres, micelles, vesicles, and dendrimers. Due to their advantages, including diversities in chemical structures, functional designability, controlled sizes, and long clearance time, synthetic polymer nanoparticles have a great potential for improving the therapeutic efficacies of drug delivery systems.^[^ ^13^, ^14^, ^15^, ^16^ ^]^ In recent years, nanomaterials have been introduced in the diabetes field, and many emerging applications for diabetes diagnosis and therapy have been proposed based on these nanomaterials. Nanomaterial‐assisted biomedicine has shown significant advantages in almost all kinds of diabetes‐associated diagnosis and therapy, i.e., diabetic biomarker detection, glucose control, insulin mimicking, and prevention of complications.

**Figure 3:**
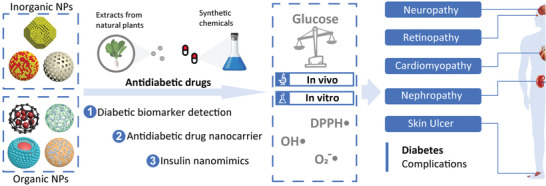
General overview of current nanomaterials for diabetes theranosis and their implications.

In this review, we provide a general overview of current nanomaterials in diabetes and their implications (**Figure** **3**). We elucidate on representative nanomaterials for diabetes‐related therapy and early diagnosis. We described semi‐conductive nanoparticles, functional polymer nanomaterials and their composites, which enable specific detection of various diabetic biomarkers. The potential of nanomaterials as biosensors for early diabetes diagnosis is also discussed. Then, we discussed diabetic nanotherapy. Functional nanomaterials as antidiabetic drugs or as drug carriers for diabetes treatment and glucose control are emphasized. In addition to diabetes treatment, recent developments in nanomaterials for the prevention of typical diabetic complications are also highlighted. Finally, we provide perspectives in this field, including current challenges and future trends of nanomaterials for diabetes. This review provides readers with an overview of nanomaterial‐assisted theranostics of diabetes and shows the potential for exploiting novel nanomaterials with unique functions and properties for better applications in diabetes.

## 2 Nanomaterials for Diabetes Diagnosis

Diagnosis is a prerequisite for disease treatment. Early diagnostic testing might identify individuals in need of interventions, such as lifestyle management or pharmacological approaches to prevent dysglycemia or the onset of disease‐associated complications.^[^ ^17^ ^]^ For instance, treatment of Type‐II diabetes involves participation in physical activities and eating healthy diets.^[^ ^18^ ^]^ Conventional methods for diabetes diagnosis are based on plasma glucose levels criteria, with a major reliance on fasting plasma glucose (FPG) levels, 2 h plasma glucose (2 h PG) levels during a 75 g oral glucose tolerance test (OGTT), or the hemoglobin‐A1c criteria.^[^ ^19^ ^]^ However, approaches for determining blood glucose levels are deemed painful by some patients while measured values vary depending on various factors, including age, time of testing, and other physiological conditions. In relative terms, A1c testing is widely for chronic hyperglycemia, since it can reflect a 2 to 3 month average of blood glucose levels. However, due to laborious, time‐consuming whole blood processing and bulky instruments, A1c testing is restricted to hospitals and central laboratories.^[^ ^20^ ^]^ To overcome the limitations associated with these traditional diagnostic methods, biosensors that are based on emerging nanotechnologies have been developed to enable timely and non‐invasive diabetes diagnosis.^[^ ^21^ ^]^

### 2.1 Nanosensors for Acetone Gas Detection in Human Breath

Abnormal acetone levels in human breath are a specific marker for noninvasive diabetes diagnosis.^[^ ^22^ ^]^ Concentrations of exhaled acetone from diabetes patients exceed 1.8 ppm, while the value for healthy people is only 0.3–0.9 ppm.^[^ ^23^, ^24^ ^]^ Currently, chemiresistive sensors that are capable of detecting acetone have been used in diabetes diagnosis. Semiconductor metal oxides (e.g., In_2_O_3_, CeO_2_, WO_3_, SnO_2_, and ZnO)^[^ ^25^, ^26^ ^]^ have been broadly used in chemiresistive sensing of gases. The sensing mechanism relies on changes in electrical conductivity in the presence of gases and oxygen, which lead to catalytic reduction/oxidation reactions at the metal oxide surface. When semiconductor metal oxides are exposed to air, O_2_ is chemically adsorbed (O_2_ + 2e^‐^ → 2O^‐^_(ads)_) under high operating temperatures, leading to formation of electron depletion regions on surfaces. When exposed to reducing gas atmospheres, such as acetone, trapped electrons in chemisorbed oxygens are donated to SMOs as shown in the following reaction: CH_3_COCH_3_ (gas) + 2O^‐^_(ads)_ → CH_3_O^‐^ + C^+^H_3_ + CO_2_ (gas) + 2e^‐^, leading to a decrease in resistance and specific acetone sensing. Chemiresistive gas sensors are advantageous for portable devices, including miniaturization, low power consumption, as well as low‐cost mass production.

#### 2.1.1 WO_3_‐Based Nanosensors

**Figure 4:**
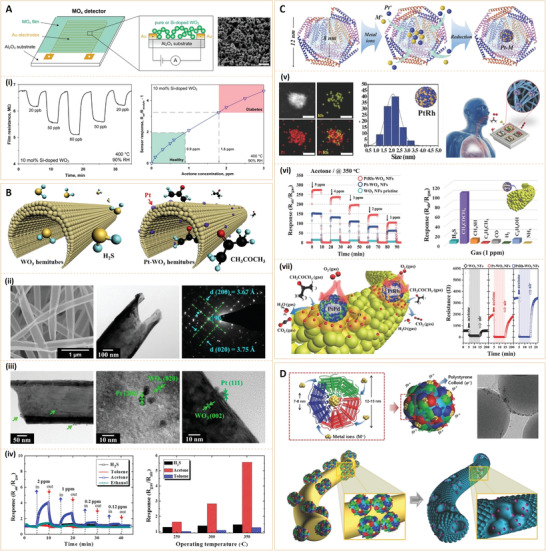
A) Detector schematic (a metal oxide (MOx) film is deposited onto a sensor substrate consisting of an Al_2_O_3_ support with interdigitated Au electrodes) and a scanning electron microscope (SEM) image of a pure WO_3_ film directly deposited onto the sensor substrate after in situ annealing. (i) WO_3_ sensor resistance at 90% of relative humidity (RH) and upon exposure to different ultralow acetone concentrations (20, 50, and 80 ppb) at 400 °C (Left); Sensor responses for acetone detection (0–3 ppm) of a 10 mol% of Si‐doped WO_3_ sensor with 90% of RH at 400 °C (Right). Reproduced with permission.^[^ ^22^ ^]^ Copyright 2010, American Chemical Society. B) Schematic presentation of polycrystalline WO_3_ hemitube optimized for H_2_S detection and Pt functionalized WO_3_ hemitube for selective acetone detection. (ii,iii) Morphologies and crystal structures of Pt‐functionalized WO_3_ hemitubes. (iv) Gas sensor characterization: response of Pt catalyst decorated WO_3_ hemitubes at 300 °C (Left) and Pt‐functionalized WO_3_ hemitubes at temperature ranges of 250−350 °C (Right). Reproduced with permission.^[^ ^28^ ^]^ Copyright 2012, American Chemical Society. C) Schematic presentation of apoferritin‐encapsulating method for preparing PtM‐apo NPs. (v) As‐synthesized PtRh‐apo NPs: particle size distribution histogram (Left) and energy dispersive spectroscopy (EDS) elemental mapping (Right). (vi) Acetone sensing property of PtRh‐WO_3_ NFs at 350 °C: Response property at concentration ranges of 1–5 ppm (Left) and selective property toward 1 ppm, as well as response towards seven interfering gases (Right). (vii) Sensing mechanism of PtPd‐, or PtRh‐WO_3_ NFs (Left) and dynamic resistance change transients: pristine WO_3_ NFs, Pt‐, PtRh‐WO_3_ NFs toward 1 ppm acetone at 350 °C (Right). Reproduced with permission.^[^ ^29^ ^]^ Copyright 2017, WILEY‐VCH. D) Schematic presentation of Apof‐catalyst nanoparticles (NPs) and self‐assembly of Apof‐catalyst NPs on polystyrene (PS) colloid templates. Reproduced with permission.^[^ ^30^ ^]^ Copyright 2016, WILEY‐VCH.

Chemo‐resistive detectors that are based on semiconductor WO_3_ nanofilms have been used in the analysis of exhaled breath acetone.^[^ ^27^ ^]^ This is because spontaneous electric dipole moments of the *ε*‐phase of WO_3_ nanoparticles enhance interactions between sensor and acetone with high dipole moments. Sensing responses of metal oxide films are highly dependent on their surface structures and morphologies. For high sensitivity and fast response rates, sensing materials should have large specific surface areas and high accessibility of gas molecules to material surface by diffusion. In this regard, nanomaterials have unique advantages. Righettoni et al.^[^ ^22^ ^]^ used pure and Si‐doped WO_3_ nanoparticles (10–13 nm in diameter) with a gas phase to direct deposition onto interdigitated electrodes, resulting in a novel acetone detector (**Figure** **4**A). Ultrasmall nanoparticles increase specific surface areas and enhance the accessibility of sensors to acetone molecules. They found that at 400 °C and 90% relative humidity, healthy humans (≤900 ppb acetone) and diabetic patients (≥1800 ppb) can be clearly distinguished by a remarkable gap (40%) in sensor responses.

For accurate diabetes diagnosis using acetone sensors, research efforts have aimed at fabricating highly sensitive exhaled breath sensors; in particular, by combining noble catalysts and unique metal oxide nanostructures that have a high surface area and high porosity. Choi et al.^[^ ^28^ ^]^ developed a Pt‐functionalized WO_3_ hemitube for acetone detection by using electrospun nanofibers as sacrificial templates and subsequently, coating WO_3_ films on the polymeric nanofibers, followed by high temperature calcination (Figure 4B). Pt‐functionalized WO_3_ hemitubes with wall thickness of 60 nm exhibited superior acetone sensitivities (*R*_air_/*R*_gas_ = 4.11 at 2 ppm) with negligible H_2_S responses. They also performed a cyclic test of Pt‐functionalized WO_3_ hemitubes after 7 months and found that selectivity and sensitivity were confined to acetone, indicating its potential as a platform for diabetes diagnosis by detecting acetone traces in human breath.

Compared to monometallic nanoparticles, Pt‐based bimetallic nanoparticles are potential oxygen reduction, methanol oxidation, and hydrogen oxidation catalysts. Kim et al.^[^ ^29^ ^]^ proposed a novel bimetallic nanoparticle employing bimetallic Pt‐based nanoparticles (PtM, where M = Pd, Rh, and Ni) via a protein encapsulating route supported on mesoporous WO_3_ nanofibers (NFs), for detecting acetone gas in highly humid exhaled breath (Figure 4C). The PtRh‐WO_3_ nanofibers (NFs) showed excellent acetone responses (*R*_air_/*R*_gas_ = 104 at 1 ppm.), which better than those of Pt‐WO_3_ NFs (*R*_air_/*R*_gas_ = 61 at 1 ppm) and pristine WO_3_ NFs (*R*_air_/*R*_gas_ = 5.7 at 1 ppm). In addition, PtRh‐WO3 NFs have excellent acetone selectivities with negligible cross‐responses towards other interfering gases. Due to their synergistic effects, the superior catalytic activities of PtRh nanoparticles and the additional oxide states of the bimetallic alloyed nanoparticles enhance acetone responses. In addition to using bimetallic noble catalysts for improved sensing, they also attempted to prepare porous WO_3_ fibers to increase the surface area of WO_3_‐based acetone sensor. They proposed a novel noble metallic catalyst functionalization method, based on protein‐encapsulated metallic nanoparticles and their self‐assembly on polystyrene (PS) colloid templates to form catalyst‐loaded porous WO_3_ NFs (Figure 4D).^[^ ^30^ ^]^ The PS templates generated open pores on electrospun WO_3_ NFs, thereby facilitating acetone gas transport into sensing layers and promoting active surface reactions. Compared to the dense WO_3_ NFs (*R*_air_/*R*_gas_ = 7.7), the Pt NP‐loaded porous WO_3_ NFs exhibited enhanced acetone sensitivities (*R*_air_/*R*_gas_ = 28.9). Therefore, the utilization of polymeric colloids as sacrificial templates to form well‐distributed open pores on semiconductor metal oxide surfaces can serve as a novel means for optimization of breath biomarker sensing performance.

#### 2.1.2 SnO_2_‐Based Nanosensors

SnO_2_ has been investigated as semiconductor metal oxide for acetone gas sensing. SnO_2_‐based acetone sensors have limitations in sensitivity and selectivity. To enhance their sensitivity to target gas species, efforts have aimed at tailoring their micro/nanostructures or integrating nanosized catalysts. Among them, 1D SnO_2_ nanotubes (high surface area), decorated with nanocatalysts exhibit great promise at improving sensing by maximizing the ratio of reactive sites at both inner and outer surfaces as well as electronic or chemical sensing properties of nanocatalysts.

**Figure 5:**
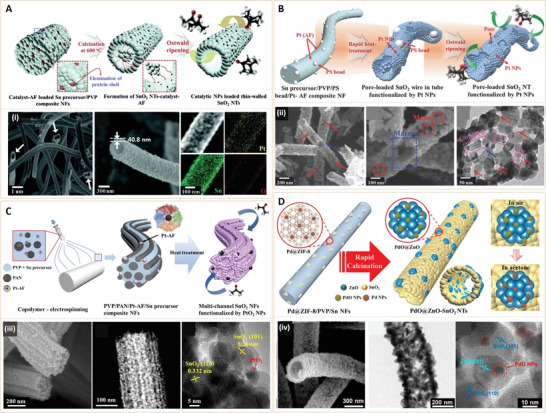
A) Schematic presentation of the processing steps of SnO_2_ NTs functionalized by bio‐inspired catalysts (Pt‐loaded SnO_2_ NTs and Au‐loaded SnO_2_ NTs). (i) SEM images of: Pt‐ loaded SnO_2_ NTs (Left), cross‐sectional Pt‐loaded SnO_2_ NTs (Middle) and EDX elemental mapping of Pt‐loaded SnO_2_ NTs (Right). Reproduced with permission.^[^ ^31^ ^]^ Copyright 2015, The Royal Society of Chemistry. B) Schematic presentation of Sn precursor/PVP/PS bead/Pt@AF composite NF, pore loaded SnO_2_ wire in tube functionalized by Pt NPs, and pore loaded SnO_2_ NTs functionalized by Pt NPs. (ii) SEM (Left, Middle) and TEM (Right) images of Pt‐PS_SnO_2_ NTs. Reproduced with permission.^[^ ^32^ ^]^ Copyright 2016, WILEY‐VCH. C) Schematic presentation of copolymer‐electrospinning, as‐spun PVP/PAN/Pt‐AF/Sn precursor composite NFs, and PtO_2_‐SnO_2_ MCNFs. (iii) SEM images of PtO_2_‐SnO_2_ MCNFs (Left), TEM (Middle) and HRTEM (Right) of PtO_2_‐SnO_2_ MCNFs. MCNFs, multichannel NFs; PVP, polyvinylpyrrolidone; PAN, polyacrylonitrile; HRTEM, high‐resolution transmission electron microscopy. Reproduced with permission.^[^ ^33^ ^]^ Copyright 2018, American Chemical Society. D) Schematic presentation of the synthetic process of PdO@ZnO‐SnO_2_ NTs. (iv) SEM (Left), TEM (Middle) and HRTEM (Right) images of PdO@ZnO‐SnO_2_ NTs. NTs, nanotubes. Reproduced with permission.^[^ ^34^ ^]^ Copyright 2018, American Chemical Society.

Kim et al.^[^ ^31^ ^]^ developed an SnO_2_‐based nanosensor using Pt nanoparticle‐immobilized thin‐walled SnO_2_ nanotubes by taking into account the electronic and chemical sensitization properties of Pt nanoparticles (**Figure** **5**A). The acetone nanosensor was synthesized via the hollow protein nanocage (e.g., Pt‐encapsulated apoferritin) template route and directly functionalized on the interior and exterior walls of electrospun SnO_2_ nanotubes. Functionalization was performed during controlled single‐nozzle electrospinning, followed by high‐temperature calcination with heating rate control. When Pt was oxidized to PtO*_x_* (p‐type semiconductor), it increased the depletion region by forming p–n junctions at the interface between SnO_2_ (n‐type) and PtOx NPs. Subsequently, the thick depletion region enhanced sensitivity to acetone molecules that donate electrons to SnO_2_. The catalytic effect of Pt NPs enhanced sensitivity and selectivity to acetone. Pt‐loaded SnO_2_ nanotubes exhibited a significant enhanced sensitivity (*R*_air_/*R*_gas_ = 92 at 5 ppm) to acetone, compared to pristine SnO_2_ nanotubes (*R*_air_/*R*_gas_ = 11 at 5 ppm) and dense SnO_2_ nanotubes (*R*_air_/*R*_gas_ = 4.8 at 5 ppm). In addition, Pt‐loaded SnO_2_ nanotubes exhibited excellent sensitivity and selectivity to acetone (*R*_air_/*R*_gas_ = 25, at 1 ppm) with little cross‐sensitivity (*R*_air_/*R*_gas_ < 5, at 1 ppm) to traces (1 ppm) of interfering gases such as H_2_S, NO, toluene, CO, pentane and NH3, measured at 350 °C. Therefore, the Pt‐decorated 1D SnO_2_ nanotubes are sensitive enough for detecting diabetic gas biomarkers for diabetes monitoring. The same group prepared a Pt‐loaded 1D SnO_2_ nanotube with bimodal pore distribution (Figure 5B).^[^ ^32^ ^]^ In the study, Ostwald ripening‐driven electrospinning combined with sacrificial template route using PS colloid and Pt‐encapsulated protein apoferritin were used. Since PS colloids and protein shells are thermally decomposed during calcination of electrospun fibers, Pt nanocatalyst‐loaded 1D thin‐walled SnO_2_ nanotubes with dual‐sized pores can be easily prepared. The Pt‐decorated bimodal porous SnO_2_ nanotubes exhibited exceptionally high acetone gas responses (*R*_air_/*R*_gas_ = 192 at 5 ppm) when compared to those of the previous Pt‐loaded SnO_2_ nanotubes (*R*_air_/*R*_gas_ = 92 at 5 ppm) at 350 °C. In addition, superior selectivities against other interfering gases and very low limits of detection (10 ppb) to simulated diabetic acetone molecules were observed. The mechanism of significantly enhanced sensing characteristics can be attributed to synergistic effects of unique morphologies, i.e., highly porous tubular structures and well‐dispersed Pt/PtO*_x_* NPs on thin‐walled SnO_2_ NTs, which provides both chemical and electronic sensitization.

Since highly porous structures play vital roles in acetone responses of SnO_2_‐based nanosensors, Kim et al. further developed a PtO_2_ nanocatalyst‐loaded SnO_2_ multichannel NF for acetone sensing (Figure 5C).^[^ ^33^ ^]^ Multichannel nanofibers were synthesized by utilizing two immiscible polymers, that is, poly(vinylpyrrolidone) and polyacrylonitrile. For effective functionalization of Pt nano‐catalysts, apoferritin was used as a Pt^4+^ loading template. Due to the multichannel structure with a high porosity, effective activation of catalysts on interior and exterior sites of SnO_2_ nanofibers was realized. The PtO_2_ nano‐catalyst‐loaded SnO_2_ multichannel nanofiber exhibited significantly high acetone responses (*R*_air_/*R*_gas_  = 194.15) to 5 ppm acetone gas at 400 °C. In addition to superior selectivity to acetone molecules among various interfering gas species, the SnO_2_ nanosensor showed an excellent stability during 30 cycles of response and recovery to 1 ppm acetone gas. Principal component analysis using a sensor array of SnO_2_ and PtO_2_‐loaded SnO_2_ clearly distinguished acetone molecules from other interfering gases. The study showed the potential feasibility of Pt/SnO_2_ based nanosensors as acetone sensing platforms.

Kim et al. also improved chemical and electronic sensitization to enhance acetone sensitivity. Heterogeneous sensitization of PdO‐embedded ZnO (PdO@ZnO) complex nanocatalyst on hollow SnO_2_ nanotubes was used for acetone sensing (Figure 5D).^[^ ^34^ ^]^ Nanoscale Pd nanoparticles (≈2 nm) loaded Zn‐based zeolite imidazole framework (Pd@ZIF‐8, ≈80 nm) were used as new catalyst‐loading platforms for effective functionalization of the PdO@ZnO complex catalyst. As a result of the well‐dispersed PdO nano‐catalysts (3–4 nm), multiheterojunctions between PdO/ZnO and ZnO/SnO_2_ as well as the hollow SnO_2_ nanostructures, a high‐performance acetone gas nanosensor was obtained. It was found that heterogeneous sensitization of the PdO@ZnO catalyst on SnO_2_ nanotubes significantly improved acetone sensing performance. The synergistic enhancement was due to: (i) The *n*‐*n* heterojunction of PdO@ZnO/SnO_2_ and (ii) the catalytic effects of PdO nanoparticles. As a result, the PdO@ZnO SnO_2_ nanotubes exhibited significant responses to 1 ppm of acetone (*R*_air_/*R*_gas_ = 5.06 at 400 °C) with a high selectivity and stability. Moreover, PdO@ZnO SnO_2_ nanotubes showed rapid responses and a recovery time of less than 20 and 64 s, respectively. The proposed design concept in this study provides a new strategy for preparing heterogeneous complex catalyst loaded 1D nanotubes, which may pave way for the development of hollow architectures that are functionalized with various synergistic catalysts for diabetic gas sensing.

#### 2.1.3 Other Oxide‐Semiconductor‐Based Nanosensors

**Figure 6:**
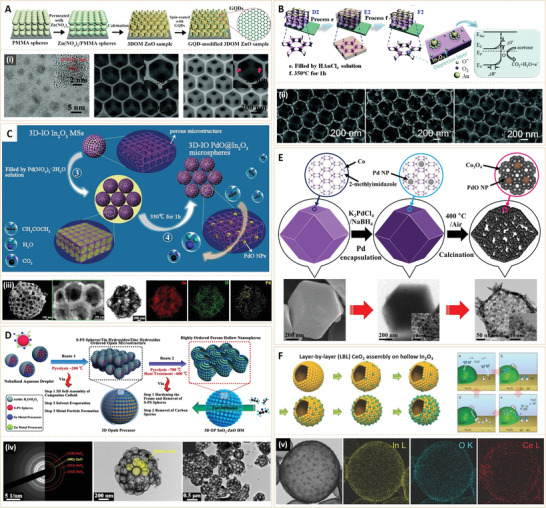
A) Schematic presentation of the synthesis process of GQD‐modified 3DOM ZnO sample. (i) TEM images of pure GQDs (Left), SEM image (Middle) of the 3DOM ZnO sample and SEM image (Right) of the GQD‐modified 3DOM ZnO sample. GOD, graphene quantum dot. Reproduced with permission.^[^ ^35^ ^]^ Copyright 2019, The Royal Society of Chemistry. B) Schematic presentation of the fabrication process of Au loaded 3DIO In_2_O_3_ films. (ii) SEM images of S3‐S5 3DIO Au/In_2_O_3_ films. Reproduced with permission.^[^ ^37^ ^]^ Copyright 2015, The Royal Society of Chemistry. C) Schematic presentation of the fabrication process of PdO‐loaded 3D‐IO In_2_O_3_ MSs. (iii) High magnification SEM, TEM images of the S3 sample and its EDS elemental mapping images of In, O, and Pd, respectively. Reproduced with permission.^[^ ^38^ ^]^ Copyright 2018, American Chemical Society. D) Synthesis procedure for 3D OP SnO_2_‐ZnO HM via self‐assembly method. (iv) SAED pattern (Left) and (Middle, Right) TEM images of the S2 sample. Reproduced with permission.^[^ ^39^ ^]^ Copyright 2018, American Chemical Society. E) Schematic presentation of the synthetic process of PdO‐Co_3_O_4_ HNCs derived from Pd@ZIF‐67 by optimized thermal treatment. Actual SEM image and TEM images are shown below the illustrations, respectively. Reproduced with permission.^[^ ^40^ ^]^ Copyright 2017, American Chemical Society. F) Schematic illustration of the synthesis process of In_2_O_3_ sensors layer‐by‐layer (LBL)‐coated with CeO_2_ nanoclusters. (v) Elemental mapping of 11.7 Ce‐In_2_O_3_ hollow spheres. Reproduced with permission.^[^ ^41^ ^]^ Copyright 2016, Wiley‐VCH.

In addition to WO_3_ and SnO_2_, there are other types of oxide semiconductor nanomaterials with porous structures and catalyst loadings, which have been widely investigated as candidates for acetone gas sensing. Due to typical adsorption–desorption mechanism of gas nanosensors, morphological and compositional control of nanoscales of other oxide semiconductors, that is, enlarging the specific surface area were evaluated. The 3D ordered mesoporous (3DOM) structures have unique advantages, including large specific surface areas and highly interconnected networks, showing the superiority for adequate gas exposure, fast electron transport, and a rapid reaction process with gas molecules. Liu et al.^[^ ^35^ ^]^ fabricated a graphene quantum dots (GQDs)‐functionalized 3DOM ZnO nanomaterial for acetone sensing (**Figure** **6**A). They found that the superior electrical conductivity of GQDs and the p–n heterojunction between p‐type GQDs and n‐type ZnO effectively enhanced resistance variation due to alterations in oxygen adsorption. This electronic characteristic, together with hierarchical pore size distribution (macroscale and mesoscale) and the high surface area (88.2 m^2^ g^−1^), endowed the ZnO‐based nanosensor with a remarkably enhanced gas sensing performance, for example, superior responses to acetone gas (*R*_air_/*R*_gas_ = 15.2 at 1 ppm), rapid response/recovery time (9/16 s) and lower theoretical detection limit (8.7 ppb). The GQD‐modified 3DOM ZnO acetone sensor, therefore, has the potential to distinguish between exhaled breath samples for healthy people and diabetics.

Researchers from the same group also fabricated a series of 3DOM ZnO‐CuO sensors,^[^ ^36^ ^]^ which were capable of detecting acetone (0.1–50 ppm) even under a high humidity. They selected gold nanoparticles (Au NPs) as catalysts for surface reactions and specific adsorption sites for oxygen, and then fabricated another 3DOM In_2_O_3_ film loaded with Au NPs to enhance electrical responses (Figure 6B).^[^ ^37^ ^]^ Due to its excellent electrical properties for gas detection, In_2_O_3_ was selected as the model semiconductor oxide nanomaterial (n‐type); Due to better thermal stability when compared to Ag and lower costs when compared to Pt or Pd, Au NPs were selected as catalysts for surface reactions and specific oxygen adsorption sites. In the In_2_O_3_‐based sensors, In_2_O_3_ resistance was controlled by reactive oxygen species (O^2−^, O^−^ or O^2−^) concentrations, which reacted with reductive acetone gas, resulting in reduced semiconductor conductivity. Due to chemical sensitization mechanisms and catalytic effects of AuNPs, the resultant 3DIO Au/In_2_O_3_ sensor showed high responses to trace acetone, low detection limits (15 ppb) and good anti‐interference. Clinical tests proved that the as‐prepared 3DIO Au/In_2_O_3_ sensor can clearly distinguish and measure acetone in human breath, implying its applicability in diabetes diagnosis. Inspired by the high performance of 3DOM structures, through one‐step ultrasonic spray pyrolysis (USP) using self‐assembly sulfonated polystyrene (S‐PS) spheres as sacrificial templates, Wang et al.^[^ ^38^ ^]^ fabricated a 3D inverse opal In_2_O_3_ microsphere (3D‐IO In_2_O_3_ MS) (Figure 6C). To obtain good acetone sensing properties, catalytic palladium oxide nanoparticles (PdO NPs) were loaded on 3D‐IO In_2_O_3_ MSs via a simple impregnation method. Compared to pristine 3D‐IO In_2_O_3_ MSs, the PdO‐loaded one exhibited 3.9 times higher responses (*R*_air_/*R*_gas_ = 50.9) to 100 ppm acetone at 250 °C. In addition, the 3D‐IO PdO@In_2_O_3_ MSs exhibited excellent selectivities to acetone, with a high response of 49.7–100 ppm after a long‐term stability test at 250 °C. Using a similar method, they developed another 3D opal porous (OP) hollow microsphere (HM) that was based on SnO_2_‐ZnO semiconductor oxide nanocomposite (Figure 6D).^[^ ^39^ ^]^ Advantages of this nanosensor are associated with 3D interconnection and bimodal (mesoscale and macroscale) pores of 3D OP HM structures, as well as the n–n heterojunction‐induced change in oxygen adsorption between SnO_2_ and ZnO. The 3D OP SnO_2_‐ZnO HM sensor showed high responses and ultra‐fast dynamic process (response time ≈4 s and recovery time ≈17 s) to 1.8 ppm acetone under high humid ambient conditions (98% of RH). Moreover, it rapidly identified the states of exhaled breaths for healthy people and simulated diabetics.

The above study provides a new nanostructural concept by integrating hierarchical pores (e.g., the hollow structures here) to design gas sensors with high specific areas. Hollow nanostructures were also adopted in other semiconductor oxide‐based acetone nanosensors. Kim et al.^[^ ^40^ ^]^ developed a nanoscale PdO catalyst loaded Co_3_O_4_ hollow nanocage (HNC) for improved acetone detection by using metal‐organic framework (MOF) templates (Figure 6E). To obtain PdO‐loaded hollow Co_3_O_4_ structures, Pd nanoparticles (2–3 nm) were well dispersed in the cavity of the Co‐based zeolite imidazole framework (ZIF‐67). Subsequently, calcination of Pd‐loaded ZIF‐67 led to catalytic PdO‐functionalized Co_3_O_4_ hollow nanocages (PdO‐Co_3_O_4_ HNCs). Figure 6E shows that the ultrasmall PdO NPs (3–4 nm) were well‐distributed in the walls of Co_3_O_4_ hollow nanocages (HNCs). The unique structure provided high surface areas and high catalytic activities, thereby improving acetone sensing responses when compared to PdO‐Co_3_O_4_ nanopowders. Yoon et al.^[^ ^41^ ^]^ also designed In_2_O_3_ hollow spheres for acetone sensing (Figure 6F). To address humidity dependence, an optimal concentration of CeO_2_ nanoclusters was uniformly loaded onto the In_2_O_3_ hollow spheres via layer‐by‐layer (LBL) assembly. Optimization of the CeO_2_ nanocluster‐loaded hollow In_2_O_3_ led to the synthesis of a CeO_2_/In_2_O_3_ sensor with fast response/recovery, low detection limit (500 ppb), and high selectivity to acetone, even in highly humid conditions (80% of RH). Apart from the unique hollow structure of In_2_O_3_ that was capable of improving acetone sensitivity, its self‐refreshing mechanism, which is based on facile redox reaction (Ce^3+^/Ce^4+^) of CeO_2_ nanoclusters was used in most oxide semiconductor gas sensors, which may provide a general solution for designing high‐performance gas sensors without humidity dependence.

Oxide semiconductor nanosensors are very promising for detection of diabetes‐associated trace acetone gas. However, development of ultrafast response sensors for accurate detection of trace acetone gas in human exhaled breath, and applications of these sensors in clinical diabetes diagnosis remains a challenge. Studies have increasingly focused on the practical aspects of clinical testing, and it is believed that commercially available semiconductor oxide acetone sensors will be available in the near future.

### 2.2 Nanosensors for Diabetic Biomarker Detection in Human Biofluids

In addition to acetone gas in the exhaled breath, there are various diabetes‐related biomarkers, including glucose, insulin, and glycated hemoglobin (HbA1c) in biofluids (e.g., blood, tear, and urine) that are used for diabetes diagnosis. Blood glucose levels reflect real‐time glycemic levels. However, due to its large fluctuations that depend on living conditions, such as daily diets, stress levels, and physical activity, blood glucose levels may not be a critical index for long‐term glycemic control. HbA1c reflects average blood glucose levels over 2 to 3 months, and is associated with the life span of red blood cells. Normal HbA1c (%) levels, which are related to total hemoglobin (Hb), are less than 6%, and levels greater than 9% indicate poor glycemic control. These levels have been recommended as the criterion for diabetes diagnosis by the American Diabetes Association (ADA). Therefore, it is necessary to develop different methods for different diabetes biomarkers, which may be helpful for definite diabetes diagnosis. As earlier mentioned, traditional diagnostic methods for these biomarkers have obvious limitations, therefore, biosensors can be used for real‐time rapid diabetes diagnosis. According to types of signal sources, the current nanomaterial‐based biosensors for diabetes biomarkers mainly focus on electrochemical and optical detection.

#### 2.2.1 Electrochemical Detection

**Figure 7:**
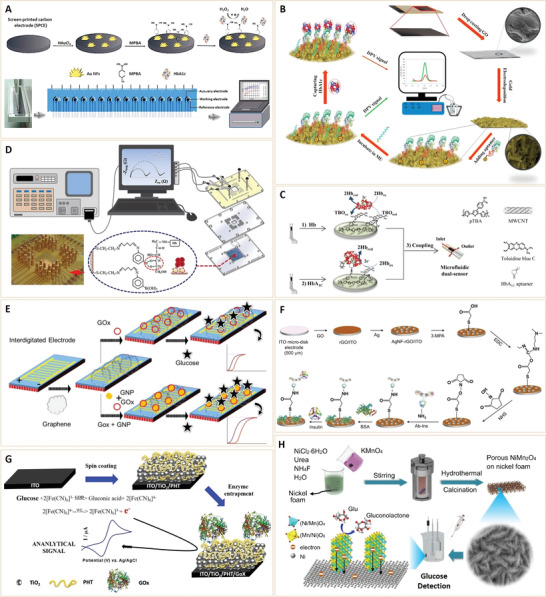
A) Schematic images of the 16‐channel screen‐printed carbon electrode (16‐SPCE) detection system, modification of 4‐MPBA (4‐mercaptobenzeneboronic acid)‐Au NFs and HbA1c detection. Reproduced with permission.^[^ ^44^ ^]^ Copyright 2019, Elsevier B.V. B) Schematic presentation of the HbA1c nano‐biosensor. Reproduced with permission.^[^ ^45^ ^]^ Copyright 2019, Elsevier B.V. C) Schematic diagram of microfluidic dual electrode and presentation of the fabrication of Hb and HbA_1C_ sensors. Reproduced with permission.^[^ ^46^ ^]^ Copyright 2016, Elsevier B.V. D) Schematic presentation of an impedance‐based label‐free affinity sensor for recognition of glycated hemoglobin (HbA1c). Reproduced with permission.^[^ ^47^ ^]^ Copyright 2016, American Chemical Society. E) Schematic presentation of graphene dielectric sensing surfaces modified with GOx or GOx‐GNP for comparisons. Reproduced with permission.^[^ ^48^ ^]^ Copyright 2019, Dove Press Ltd. F) Schematic presentation of the preparation and binding mechanism of insulin ITO electrode sensors with covalently attached receptor antibodies on the AgNF‐rGO nanocomposite. Reproduced with permission.^[^ ^49^ ^]^ Copyright 2016, Elsevier B.V. G) Illustration of ITO/TiO_2_/PHT/GOx nanohybrid sensor probe design and sensing principle. ITO, indium tin‐oxide; PHT, poly (3‐hexylthiophene); GOx, glucose oxidase. Reproduced with permission.^[^ ^50^ ^]^ Copyright 2018, Wiley‐VCH. H) Schematic illustration of the fabrication process for porous NiMn_2_O_4_ nanosheet arrays on nickel foam (NiMn_2_O_4_ NSs@NF) sensor electrode. Reproduced under the terms of the Creative Commons CC‐BY license.^[^ ^51^ ^]^ Copyright 2019, The Authors. Published by Springer Nature.

Due to their low‐cost, easy‐to‐handle, high sensitivity,^[^ ^42^ ^]^ and optimal specificity, electrochemical biosensors provide a promising point‐of‐care approach in clinical diagnosis. In cases of combined nanostructural materials and specific biomolecules (e.g., antibody or enzymes), innovative electrochemical biosensors with rapid electron/ion transfer rates, high adsorption abilities and loading as well as optional immobilization of biomolecules were developed for diabetes diagnosis.^[^ ^43^ ^]^ For example, gold nano‐flowers (Au NFs) modified screen‐printed carbon electrode electrochemical sensor has been developed for label‐free and quantitative detection of HbA1c (**Figure** **7**A).^[^ ^44^ ^]^ The electrochemical biosensor was decorated with a chemical ligand (4‐mercaptophenylboronic acid, 4‐MPBA). Phenylboronic acid of 4‐MPBA interacted with the sugar subunit of HbA1c. When HbA1c was captured by 4‐MPBA onto the electrode surface, it catalytically reduced H_2_O_2_ and resulted in specific electrochemical responses. Under optimal conditions, the sensor showed rapid HbA1c detection using a small amount of samples (<5 µL) and a wide linear dynamic range (2−20%) for HbA1c. Moreover, this method can be applied in analysis of human serum from diabetic patients.^[^ ^44^ ^]^ To enhance electrochemical sensitivity, conductive graphene nanosheets or carbon nanotubes were combined with noble metal nanoparticles (Au or Ag) for designing novel nanosensors. Omidinia et al.^[^ ^45^ ^]^ developed a HbA1c sensor by electrochemical deposition of nanocomposites of reduced graphene oxide (rGO) and gold on a cheap and flexible graphite sheet (GS) electrode, followed by modification with HbA1c specific aptamers (Figure 7B). The rGO‐Au nanocomposite enlarged the surface area, improved electron transfer on the electrode surface and augmented the signal. The rGO‐Au provided a suitable substrate for covalently bound DNA aptamer as a HbA1c receptor on the electrode surface. By using Fe(CN)_6_^3−/4−^ as the redox probe, HbA1c binding led to decreased current. The nanosensor exhibited a high sensitivity towards HbA1c with a wide linear range of 1 × 10^‐9^−13.83 × 10^‐6^ m and a low detection limit of 1 × 10^‐9^ m. Moreover, it was successfully used for measuring HbA1c in blood samples.

To accurately evaluate HbA1c levels relative to total Hb, a microfluidic amperometric dual‐sensor was developed for separate detection of HbA1c and total Hb (Figure 7C).^[^ ^46^ ^]^ To fabricate the sensor probe, poly(2,2´:5´,5″‐terthiophene‐3´‐*p*‐benzoic acid) and a multi‐wall carbon nanotube (MWCNT) composite layer (pTBA@MWCNT) was electropolymerized on a screen printed carbon electrode (SPCE), followed by immobilization of TBO for total Hb probe and aptamer/TBO for HbA1c probe, respectively. Accurate total Hb levels were determined by measuring cathodic currents of total Hb catalyzed by a toluidine blue O (TBO)‐modified working electrode. After removing unbound Hb in the fluidic channel, cathodic current related to captured HbA1c was monitored using another aptamer/TBO‐modified working electrode. After optimization of experimental conditions, the dual‐sensor determined the percentage of HbA1c in a finger prick blood sample (1 µL). This study provided a more accurate means for point‐of‐care analysis, diagnosis, and management of diabetes.

Impedance changes of electrochemical sensors can be used for detecting surface molecular binding and chemical reactions. Therefore, impedance‐based electrochemical nanosensors were developed for the recognition and detection of HbA1c. To amplify signal sensitivity, interdigitated gold microelectrode arrays (IDAs) were used (Figure 7D).^[^ ^47^ ^]^ HbA1c chemical ligand 3‐aminophenylboronic acid (APBA) was conjugated on a cysteamine self‐assembled IDAs to selectively bind HbA1c via cis–diol/phenylboronic acid interactions. Without the use of biomolecular recognition elements (e.g., antibodies), the impedance sensor had the merit of great simplicity and robustness. As expected, sensing performance was shown to be highly responsive to HbA1c in clinically relevant levels (0.1–8.36%) with a detection as well as quantitation limits of 0.024% (3*σ*/slope) and 0.08% (10*σ*/slope), respectively. Moreover, electrodes for HbAc1 binding were reversible, and showed advantages such as reusability, stability, reproducibility, which were compared with a commercial method using patient‐derived blood samples.

In addition to HbA1c, glucose and insulin blood levels were considered for electrochemical sensing and diabetes diagnosis. Gold or silver nanoparticle‐based sensors, gold‐graphene nanocomposites on a dielectric surface (Figure 7E),^[^ ^48^ ^]^ and silver nanoflower‐reduced graphene oxide composite based microdisk electrode (Figure 7F),^[^ ^49^ ^]^ have been developed for glucose and insulin detection. The common feature in these studies is that the noble metal or graphene NPs can be decorated with specific affinities toward glucose or insulin, and the excellent electrochemical properties and increased specific surface areas of nano‐Au or Ag greatly contribute to the high sensitivity and specificity for diabetes biomarker detection.

Since glucose oxidation by glucose oxidase (GOx) can produce electrons that affect current signals or electrochemical impedance of an electrode, GOx‐functionalized electrochemical sensors have the potential for glucose detection.^[^ ^50^, ^52^ ^]^ Kadian et al. synthesized TiO_2_ NPs that comprised poly (3‐hexylthiophene) (PHT) nanohybrids, which were deposited onto an indium‐tin‐oxide (ITO) glass substrate, followed by immobilization of glucose oxidase (GOx) for glucose detection (Figure 7G).^[^ ^50^ ^]^ The developed biosensor was able to specifically detect glucose with detection limits at 0.62 ± 0.02 and 0.54 ± 0.02 mg dL^‐1^ in a standard buffer and human saliva samples, respectively. The highly specific performance of this sensor towards glucose indicated the potential of GOx‐functionalized sensors for glucose detection in real sample matrices.

However, the greatest limitation of enzymatic glucose sensors is their short shelf‐time and high costs that are associated with their intrinsic dependence on enzymatic or antibody activities. To address this limitation, studies have aimed at developing nonenzymatic amperometric glucose sensors that are based on noble metal or metal oxide NPs.^[^ ^51^, ^53^, ^54^, ^55^, ^56^ ^]^ These nanostructured electrocatalysts significantly improved glucose oxidation, due to their higher active surface areas, thus attracting their applications in glucose sensing. Zhang et al.^[^ ^51^ ^]^ developed a porous NiMn_2_O_4_ nanosheet array on nickel foam (NiMn_2_O_4_ NSs@NF) fabricated by simple hydrothermal and heat treatment (Figure 7H). Due to its strong electro‐site based on generation of two redox couples of Ni^2+^/Ni^3+^ and Mn^3+^/Mn^4+^ in alkaline solutions, the transition metal oxide, NiMn_2_O_4_, has high electrical conductivities and good electrochemical performances as electrode materials. In their design, the porous NiMn_2_O_4_ NSs@NF electrode provided abundant accessible active sites, effective ion‐transport pathways, and highly efficient electron transport pathways for electrochemical catalytic reactions, thereby showing excellent sensitivities and low detection limits for glucose detection. Excellent properties of this porous NiMn_2_O_4_ NSs@NF electrode suggest the potential for applications of nonenzymatic electrochemical glucose sensors in the clinical detection of glucose.

The two strategies, incorporating nanostructures in enzyme‐based and nonenzymatic glucose sensing, have their own limitations. The former can enhance sensitivity while their shelf‐life will be reduced and biofouling problems will rise. Moreover, since measurements are frequently made, in particular, by individuals with no specific training, sample handling processes should be as simple as possible. For nonenzymatic sensors that are based on transition metal oxides, sample testing processes have limited dynamic ranges or operating conditions that are far from physiological conditions.^[^ ^57^ ^]^ It is difficult to prepare novel nanomaterials that can nonenzymatically oxidize glucose at physiological pH values with high selectivities and sensitivities.^[^ ^58^ ^]^ Majority of the nonenzymatic approaches use complex strategies, such as the incorporation of binary or tertiary nanostructures, which limits their clinical applications. Therefore, although nonenzymatic nanomaterials can overcome some of the limitations, their clinical efficiencies are low when compared to those of enzymatic sensors that are currently in use.

#### 2.2.2 Optical Detection

With rapid developments in nanotechnology and photonic materials, nanosensors that are based on detection of optical signals have drawn great interest in the biomedical field.^[^ ^59^, ^60^, ^61^, ^62^, ^63^ ^]^ Recently, optical sensors based on fluorescence, chemiluminescence, colorimetry, surface plasmon resonance (SPR), surface‐enhanced Raman scattering (SERS), and photonic materials with analyte‐triggered color‐changing properties have been extensively explored for the detection of diabetes biomarkers. These optical signals can provide qualitative and quantitative results for analytes, thus optical sensors may provide more intuitive conclusion (e.g., visualization of the analytes) on the detection processes, even without using other equipment.

##### Spectroscopic Methods

Spectroscopic sensors use specific optical signals of matter, and thus, probe the presence and levels of analytes in samples for detection. The ability of fluorescent nanosensors for biomarker detection has been evaluated, and they are commonly prepared by incorporating analyte‐sensitive and reference fluorophores into nanoparticle matrices. Currently, organic fluorescent small molecules, quantum dots (QDs), 2D transition metal dichalcogenides (TMDs), upconversion nanoparticles (UCNPs), Au nanoclusters (AuNCs), MOFs, covalent organic frameworks (COFs), and single‐walled carbon nanotubes (SWCNTs) have been used as fluorescent sources for these nanosensors.^[^ ^64^, ^65^, ^66^, ^67^, ^68^, ^69^, ^70^, ^71^, ^72^, ^73^, ^74^, ^75^, ^76^, ^77^, ^78^ ^]^

**Figure 8:**
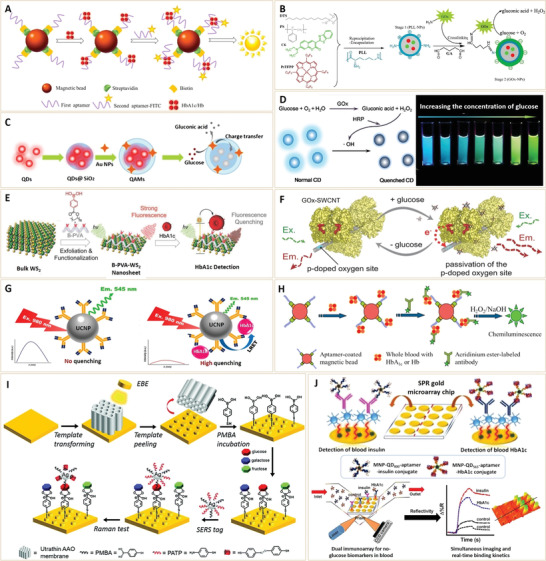
A) Illustration of experimental procedures for measuring Hb and HbA1c levels in human blood. Reproduced with permission.^[^ ^75^ ^]^ Copyright 2016, Elsevier B.V. B) Schematic presentation of the synthesis of photoluminescent glucose nanosensor GOx‐NPs. Reproduced with permission.^[^ ^64^ ^]^ Copyright 2015, Elsevier B.V. C) Illustration of the procedures for preparing QAMs and glucose detection. QAMs, QD (CdTe quantum dots)‐Au NP@silica mesoporous microspheres. Reproduced with permission.^[^ ^65^ ^]^ Copyright 2014, Elsevier B.V. D) Mechanism of CD quenching by the GOx/HRP bioenzyme system. HRP, horseradish peroxidase. Reproduced with permission.^[^ ^71^ ^]^ Copyright 2018, Elsevier B.V. E) Schematic illustration of simultaneous exfoliation and functionalization of WS_2_ nanosheets with B‐PVA and B‐PVA‐WS_2_ nanosheet based optical sensors for selective detection of HbA1c. B‐PVA‐WS_2_, boronic acid‐modified poly(vinyl alcohol)‐functionalized WS_2_ nanosheets. Reproduced with permission.^[^ ^67^ ^]^ Copyright 2018, WILEY‐VCH. F) Schematic presentation of sensing mechanism for GOx‐SWCNTs. Reproduced with permission.^[^ ^69^ ^]^ Copyright 2017, Wiley‐VCH. G) Schematic presentation of luminescence resonance energy transfer (LRET)‐based immunosensor: (Left) In the absence of HbA1c; (Right) In the presence of HbA1c. Reproduced with permission.^[^ ^77^ ^]^ Copyright 2016, American Chemical Society. H) Schematic illustration of experimental processes for measuring Hb and HbA1c. Reproduced with permission.^[^ ^79^ ^]^ Copyright 2015, Elsevier B.V. I) Schematic illustration of the fabrication of Au‐ND metal surface and corresponding flow chart for glucose detection based on the Au‐ND/glucose/Ag NPs sandwich structure. ND, nanodisk. Reproduced with permission.^[^ ^73^ ^]^ Copyright 2020, Royal Society of Chemistry. J) Assay process for measuring clinical levels of insulin and HbA1c in 20 × dilute whole blood in PBS. PBS, phosphate buffer saline. Reproduced with permission.^[^ ^80^ ^]^ Copyright 2017, American Chemical Society.

The principle of the earliest fluorescent nanosensors is based on the specific labeling of target analytes. For example, to measure blood HbA1c concentrations, fluorescent aptamers with a high affinity and specificity for HbA1c were used (**Figure** **8**A). Li et al.^[^ ^75^ ^]^ developed a fluorescent microfluidic system for automatic HbA1c measurements by combining a microfluidic system and a HbA1c‐specific fluorescent aptamer. The fluorescent microfluidic system has the potential for being a point‐of‐care device for screening and managing diabetic patients. However, such kinds of analyte‐specific labeling methods may not be suitable for other diabetes biomarkers, such as glucose, since glucose‐targeting options are relatively less than those of HbA1c. The reducibility of glucose provides another hint for fluorescence glucose detection. Given this, a novel photoluminescent glucose nanosensor was prepared by coupling glucose oxidase (GOx) with polymeric nanoparticles, in which a fluorescent dye, coumarin 6, and an oxygen‐sensitive probe, Pt(II)‐meso‐tetra(pentafluorophenyl)porphine, were incorporated (Figure 8B).^[^ ^64^ ^]^ The GOx molecules residing on nanoparticle surfaces can catalyze glucose at the expense of oxygen, thereby producing an oxygen‐sensitive fluorescence. In this case, glucose calibration can be performed with ratiometric photoluminescence and time‐resolved fluorescence (TRF).

Compared to organic small molecular fluorescent probes, semiconductor quantum dots (QDs) have excellent optoelectronic properties, including high quantum yields, broad excitation ranges, narrow and symmetric emission, high photostability, as well as long fluorescence lifetimes among others.^[^ ^81^, ^82^, ^83^ ^]^ Since hydrogen peroxide can quench the photoluminescence signal of QDs, NPs functionalized with GOx‐like activities have the potential for glucose detection. As a biomimetic catalyst, Au NPs can catalytically oxidize glucose as glucose oxidase (GOx)‐mimicking and produce hydrogen peroxide. In view of this, Au NPs were modified, QD‐encapsulated mesoporous silica microsphere (QD‐Au NP@silica) was fabricated as a novel enzyme‐mimicking nanosensor for glucose detection (Figure 8C).^[^ ^65^ ^]^ In the presence of glucose, hydrogen peroxide (H_2_O_2_) was catalytically produced. Due to charge transfer, H_2_O_2_ quenched the photoluminescence (PL) signal of QDs in the microsphere core. In this study, the QD‐Au NP@silica nanosensor was successfully used for glucose detection in human serum samples with a good accuracy and precision.

In addition to semiconductor QDs, carbon QDs (CDs) are able alternatives to QDs, and due to their low toxicity, biocompatibility, and chemical stability, they are especially suitable for biosensor applications. Electron‐transfer properties of CDs were used for glucose sensing, based on monochromatic fluorescence turn‐on/turn‐off mode.^[^ ^84^ ^]^ Moreover, Cho and Park developed a ratiometric fluorescence glucose biosensor by combining two fluorescence fluorophores (CDs and rhodamine 6G) to induce color changes during glucose detection (Figure 8D).^[^ ^71^ ^]^ Fluorescence color change was realized by fluorescence quenching, due to bienzymatic reactions of glucose oxidase (GOx) and horseradish peroxidase (HRP) with glucose. In case of excitement at 360 nm, a ratiometric fluorescence color change from blue to green was observed as glucose concentrations increased. Moreover, they fabricated a stable solid‐state biosensor film by immobilizing CD/Rh6G/GOx/HRP in a photo‐crosslinked hydrogel film. Apart from the possibility of detecting glucose using the naked eye, this approach expanded the potential of CDs‐based glucose biosensors as convenient and practical detection approaches.

Fluorescence properties of 2D transition metal dichalcogenides (TMDs) were also used for biomolecular sensing. It has been reported that simultaneous exfoliation and functionalization of fluorescent 2D WS_2_ nanosheets with HbA1c recognition abilities have HbA1c‐dependent fluorescence quenching properties (Figure 8E).^[^ ^67^ ^]^ Boronic acid‐modified poly(vinyl alcohol) (B‐PVA) was used for exfoliation of bulk WS_2_ as well as chemical recognition elements for HbA1c. Fluorescence of resultant B‐PVA‐WS_2_ nanosheets was found to be linearly quenched as HbA1c concentrations increased, even in the presence of high glucose concentrations. The study showed a high selectivity of B‐PVA‐WS_2_ nanosheets for HbA1c, suggesting their potential for fluorescent sensing of HbA1c in diabetic patients. In addition to 2D TMDs, SWCNTs can also exhibit intrinsic near‐infrared fluorescence, which has the potential for biosensing, even in vivo. Zubkovs et al.^[^ ^69^ ^]^ developed a reversible, mediatorless, near‐infrared glucose sensor that is based on GOx‐wrapped SWCNTs (GOx‐SWCNTs) (Figure 8F). The GOx‐SWCNTs were able to selectively increase fluorescence in the presence of aldohexoses, thereby showing strong responses to glucose. They found that glucose removal reversed the doping effects, resulting in a full recovery of fluorescence intensity. Reversible and near‐infrared fluorescence of GOx‐SWCNTs implies that it is as an implantable sensor for continuous glucose monitoring.

When it comes to near‐infrared fluorescence, upconversion NPs (UCNPs) are a class of nanomaterials that have to be mentioned. UCNPs are lanthanide‐doped nanomaterials that are capable of converting near‐infrared radiation into visible radiation through a nonlinear optical process. These NPs have a sharp emission bandwidth, low toxicity, high chemical stability, deep NIR light penetration into tissues, and a high signal‐to‐noise ratio when compared to traditional fluorescent labels like organic small molecules and QDs.^[^ ^85^ ^]^ In view of these advantages, Kim et al.^[^ ^77^ ^]^ developed a UCNPs immunosensor that was based on luminescence resonance energy transfer (LRET) to detect HbA1c (Figure 8G). The system used NaYF_4_:Yb^3+^, Er^3+^ as the donor and HbA1c as the acceptor, because HbA1c absorption at 541 nm corresponds with UCNPs emission. The anti‐HbA1c monoclonal antibody‐functionalized UCNPs can detect HbA1c and initiate LRET under 980 nm irradiation, leading to quenching of upconversion luminescence. The HbA1c concentration‐dependent luminescence quenching in this study was successfully applied in detection of HbA1c in blood samples. Compared to the above described photoluminescent labels, chemiluminescent molecules with light emission triggered by chemical treatment can also be applied in diabetes‐related fluorescence biosensors. For example, the acridinium‐labeled antibody was used to introduce fluorescence signals and molecular specificity into a microfluidic system for HbA1c detection (Figure 8H).^[^ ^79^ ^]^ The sensing mechanism is comparable to that of photoluminescent sensors. The linear relationship between chemiluminescent signals and HbA1c levels provides a reliable fluorescent method for HbA1c determination.

Apart from the above fluorescent nanosensors, there are two more types of spectroscopic sensors for diabetic biomarker detection, such as SERS and SPR. Due to its molecular fingerprinting ability, high sensitivity in single‐molecule levels, and anti‐interference in blood–tissue matrix, SERS is a powerful analytical method for glucose detection.^[^ ^86^ ^]^ The strong near‐field coupling between the Au film and Ag NPs has been used to obtain highly sensitive SERS glucose sensors.^[^ ^87^ ^]^ Long et al.^[^ ^73^ ^]^ designed a SERS‐based hybrid sensing substrate consisting of Ag NPs modified with Raman tag coupled to a phenylboronic acid (PBA)‐functionalized gold nano‐disk metasurface for non‐destructive glucose sensing (Figure 8I). They used quasi‐periodic structures for An NPs coupling, which led to spatially broad and increased intensities in plasmonic hotspots. Experimental results confirmed that SERS signals toward glucose were significantly enhanced by a factor of ≈8 × when compared to the substrate of smooth Au films and Ag NPs. Moreover, it showed a high selectivity as well as sensitivity to glucose over analogs like fructose and galactose, greatly promoting the applications of SERS‐based nanosensors for scalable and cost‐effective diagnosis of diabetes in future.

Compared to SERS sensing mechanisms, SPR reflects changes in refractive index upon binding of molecules onto the metal surface that is in contact with the dielectric medium (e.g., buffer solution). When an SPR chip is functionalized with analyte‐specificity, it can specifically detect target analytes. In a recent study, HbA1c and insulin‐targeted SPR gold microarray chip were designed for sensing blood HbA1c and insulin levels (Figure 8J).^[^ ^80^ ^]^ They used aptamers as capture probes attached to magnetic nanoparticles (MNPs) decorated with two distinct QDs to selectively isolate insulin and HbA1c. Using a surface plasmon microarray with validation by fluorescence imaging, the SPR immunoarray sensor could simultaneously measure blood insulin and HbA1c levels that were relevant to diabetic and prediabetic disorders. The study showed its considerable significance for clinical diagnosis of nonglucose diabetes biomarkers, which may inform better treatment approaches, prediction of complications, and management of diabetes.

In summary, the ability of spectroscopic nanosensors for diabetic biomarker detection has been evaluated. The direct or indirect optical signals reflected by biomarker binding processes provide reliable sensing mechanisms. The increased accuracy in detection of diabetic biomarkers based on these spectroscopic methods will greatly improve the efficiency of clinical diabetes diagnosis. These spectroscopic sensors need sophisticated spectrum instruments, and are not capable of rapid, intuitive, and qualitative diagnosis without special instruments, leading to difficulties in their miniaturization.^[^ ^58^ ^]^ Therefore, new sensing methods that are characterized by simplicity, convenience, rapidity, and more attractively, visual detection are being assessed for point‐of‐care disease diagnosis.

##### Coloration Method

**Figure 9:**
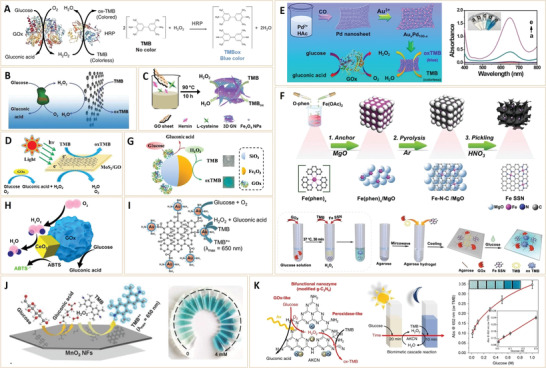
A) Glucose detection using enzymes: colorimetric detection of glucose using GOx and HRP. Reproduced under the terms of the Creative Commons CC‐BY license.^[^ ^88^ ^]^ Copyright 2019, The Authors. Published by Springer Nature. B) Schematic illustration of colorimetric detection of glucose using GOx and GO‐COOH‐catalyzed reactions. Reproduced with permission.^[^ ^90^ ^]^ Copyright 2010, WILEY‐VCH. C) Schematic presentation for Fe_3_O_4_ NPs loaded 3D graphene nanocomposites with peroxidase‐like activities. Reproduced with permission.^[^ ^92^ ^]^ Copyright 2017, American Chemical Society. D) Schematic presentation of glucose detection with GOx and MoS_2_/GO‐catalyzed reactions under light irradiation. Reproduced with permission.^[^ ^93^ ^]^ Copyright 2015, Elsevier B.V. E) Illustration for constructing 0D/2D Au*_x_* Pd_100‐_ *_x_* nanostructures for glucose detection. Reproduced with permission.^[^ ^94^ ^]^ Copyright 2020, American Chemical Society. F) Schematic illustration for preparation of single iron site nanozyme (Fe SSN), modified colorimetric detection and integrated agarose‐based hydrogel colorimetric detection of glucose. Reproduced with permission.^[^ ^95^ ^]^ Copyright 2020, WILEY‐VCH. G) Schematic illustration of peroxidase‐like activities of JFSNs in catalysis of TMB−H_2_O_2_ system and using GOx‐JFSNs as biosensing platforms for colorimetric detection of H_2_O_2_ and glucose. JFSNs, Janus *γ*‐Fe_2_O_3_/SiO_2_ nanoparticles; TMB, 3,3′,5,5′‐tetramethylbenzidine. Reproduced with permission.^[^ ^96^ ^]^ Copyright 2015, American Chemical Society. H) Schematic illustration of cascade reactions catalyzed by hybrid CeO_2_/GOx complexes. Reproduced with permission.^[^ ^97^ ^]^ Copyright 2019, Chinese Chemical Society and Institute of Materia Medica, Chinese Academy of Medical Sciences. Published by Elsevier B.V. I) Schematic illustration of the glucose detection strategy. Reproduced with permission.^[^ ^98^ ^]^ Copyright 2018, WILEY‐VCH. J) Nonenzymatic glucose colorimetric sensing based on MnO_2_ NFs. Reproduced with permission.^[^ ^99^ ^]^ Copyright 2018, WILEY‐VCH. K) Left: Glucose detection using a synthetic bifunctional nanozyme: photocatalytic aerobic oxidation of glucose with in situ production of H_2_O_2_ on AKCN (modified GCN); Middle: Schematic presentation of cascade reaction with continuous O_2_‐purging in a batch mode; Right: Concentration–response curve with linear calibration plots (inset) and color change (inset) for glucose detection in a batch reactor. Reproduced with permission.^[^ ^88^ ^]^ Copyright 2019, The Authors. Published by Springer Nature.

Another optical analysis method for diabetes diagnosis is the colorimetric method, which is based on enzyme cascade catalysis of GOx and HRP. As the prototype enzyme pair, glucose monitoring abilities of GOx and HRP have been evaluated (**Figure** **9**A).^[^ ^88^ ^]^ Glucose oxidation in the presence of GOx generates hydrogen peroxide (H_2_O_2_). The resulting H_2_O_2_ is catalyzed by HRP to oxidize chromogenic substrates (e.g., 3,3’,5,5’‐tetramethylbenzidine, TMB), producing colored products (e.g., TMBox). Due to glucose concentration‐dependent coloration of TMB, enzyme cascade catalysis of GOx and HRP can be used for colorimetric detection of glucose levels.

Recently, due to their simple preparation, low cost, high activity, as well as long shelf‐time when compared to natural enzymes, nanomaterials with enzyme‐like properties (nanozymes)^[^ ^89^ ^]^ have attracted great research interests in biomedicine. For glucose detection, nanozymes with peroxidase‐like catalytic activities are of particular interest in colorimetric methods, since nanozymes have been shown to be perfect substitutes for HRP. Qu's group found that carboxyl‐modified graphene oxide (GO‐COOH) has intrinsic peroxidase‐like activities that can catalyze TMB reactions in the presence of H_2_O_2_ to produce the blue color TMBox (Figure 9B).^[^ ^90^ ^]^ Subsequently, they used GO‐COOH to replace the function of HRP and developed a highly sensitive and selective colorimetric glucose detection strategy that is based on the enzyme cascade of GOx and GO‐COOH. The method was successfully used for buffer solutions or diluted blood and fruit juice samples, indicating the possibility of GO‐COOH nanozyme in diabetes diagnostics and other biotechnological applications. With rapid advances in nanozymes, various nanomaterials, including noble metals, metal oxides or sulfide NPs have been used as HRP substitutes for colorimetric glucose detection. For high‐efficient detection, these peroxidase nanomimics are integrated with 2D nanosheets to fabricate porous frameworks with large specific surfaces, high conductivity, and good mechanical properties. Wang et al. combined graphene (GN) with Fe_3_O_4_ NPs through a one‐pot strategy in the presence of hemin with proper concentrations. The introduced 3D porous structure improved the peroxidase‐like activity of Fe_3_O_4_ NPs,^[^ ^91^ ^]^ as well as the catalysis velocity and its affinity for substrates (Figure 9C).^[^ ^92^ ^]^ Colorimetric determination of glucose using the nanocomposite showed a low detection limit of 0.8 × 10^‐6^ m. In another study, a MoS_2_ and graphene oxide (MoS_2_/GO) nanohybrid, which exhibited a high sensitivity, was prepared to detect glucose (Figure 9D).^[^ ^93^ ^]^ As a result of fast electron transfer and synergistic interactions at interfaces of MoS_2_ and GO, Michaelis constant of this nanohybrid was 4.35‐fold lower while the maximal reaction velocity was 3.34‐fold higher than those of HRP. Furthermore, due to photocatalytic activities of MoS_2_, peroxidase‐like activities of the nanohybrid were efficiently enhanced under visible light and near‐infrared light exposure. In addition to graphene‐based 2D nanomaterials, some 2D noble metal nanosheets exhibited similar functions. Recently, Cai et al.^[^ ^94^ ^]^ reported a 0D/2D Au*_x_* Pd_100‐_ *_x_* nanocomposite with enhanced nanozyme catalysis for sensitive glucose detection (Figure 9E). The Au*_x_* Pd_100‐_ *_x_* nanocomposites were prepared by replacing Pd atoms of 2D Pd nanosheet (NSs) with Au^3+^ ions. Due to rapid electron transfer and large specific surface area, the 0D/2D Au_x_Pd_100–x_ nanocomposite enhanced the catalysis of TMB oxidation. By combining the nanocomposite with GOx, which has a high specificity, a sensitive assay for glucose biosensing was established.

Taking these low‐dimensional NPs one‐step further, a single atom site nanozyme with peroxidase‐like activity has recently been reported by Chen et al. (Figure 9F).^[^ ^95^ ^]^ The single iron site nanozyme (Fe SSN) was prepared by a support‐sacrificed strategy using magnesium oxide powder (MgO) as the support for reactions between ferrous acetate (Fe(OAc)_2_) and 1,10‐phenanthroline monohydrate (O‐phen). After pyrolysis and pickling, the Fe SSN nanozyme was incorporated with TMB and GOx into an agarose‐based hydrogel, which was found to realize reliable visualization evaluation and quantitative glucose detection. This finding introduces a new strategy for developing highly efficient nanozymes and informs a potential strategy for point‐of‐care detection of glucose during diabetes diagnosis.

A recurring issue with the above nanozyme‐based glucose detection method is that more GOx is required to ensure enzyme cascade catalysis. As a result, integrated nanozymes with GOx and peroxidase‐like dual activity are causing increased concern.^[^ ^100^ ^]^ In this regard, Lu et al.^[^ ^96^ ^]^ reported GOx‐attached Janus NPs comprising of *γ*‐Fe_2_O_3_ and SiO_2_ for colorimetric glucose detection (Figure 9G). The multifunctional hybrid Janus NPs demonstrated GOx and peroxidase catalytic activity with high selectivity and acceptable reproducibility. Owing to the incorporation of two enzymatic activities into the Janus particles, an all‐in‐one, and reusable blood glucose sensor was formed, capable of determining glucose in complex samples, such as serum. Similarly, a hybrid multienzyme system, and CeO_2_/GOx nanocomplex, were developed via self‐assembly and reported to exhibit excellent catalytic activity for colorimetric glucose detection (Figure 9H).^[^ ^97^ ^]^ When compared to individual GOx and CeO2 NPs, the integrated CeO_2_/GOx nanocomplexes demonstrated improved efficiency, which was attributable mostly to the minimal diffusion of intermediate in the nanocomplexes system. Furthermore, the nanocomplexes exhibited long‐term stability and high recyclability, thus offering a simple and efficient example of how to construct spatially confined multi‐enzyme systems for analysis and diagnosis.

The two studies propose a logical technique for integrating GOx and peroxidase‐like activities using GOx‐functionalized nanozymes. However, one issue with the approach is the short shelf‐life of the natural enzyme (i.e., the GOx). Recently, researchers have dedicated themselves to investigating enzyme‐free multifunctional nanozymes as integrated glucose sensors. Zhang et al., for example, reported a “non‐naked” Au NP with peroxidase‐ and GOx‐like dual activity at the same pH (Figure 9I).^[^ ^98^ ^]^ Natural protein (bovine serum albumin, BSA) was used as a stabilizer and protector in the preparation of the Au NP (dubbed Au@BSA NPs). The Au@BSA NPs were efficiently designed as an all‐in‐one sensor for the rapid colorimetric detection of glucose by tandem catalysis of glucose oxidation and TBM oxidation after covalent bonding with graphene oxide (GO). Similarly, MnO_2_ nanoflakes (NFs) with controllable morphology were prepared using a BSA‐directed method (Figure 9J).^[^ ^99^ ^]^ In a comparable pH range, the MnO_2_ NFs showed dual enzyme activity (i.e., GOx‐ and peroxidase‐like dual activity). This feature also enabled tandem catalysis of glucose, which was used as a one‐pot, nonenzymatic strategy for colorimetric detection of blood glucose. In short, tandem nanozymes showed remarkable convenience in bioanalysis, breaking traditional colorimetric detection processes and achieving the purpose of “one‐pot” and “nonenzyme.”

Despite the success of enzyme‐free and dual‐functional nanozymes, the majority of them rely on expensive noble metal or toxic metal oxide catalysts. In this regard, metal‐free multifunctional nanozymes composed of earth‐abundant and low‐toxicity components are highly desired. With this idea in mind, Zhang et al.^[^ ^88^ ^]^ synthesized a metal‐free nanozyme of modified graphitic carbon nitride (GCN) and demonstrated its dual‐functional photocatalytic capabilities for GOx‐mimicking in glucose oxidation and HRP‐mimicking in chromogenic substrate (TMB) oxidation (Figure 9K). Under visible light, efficient charge separation on the modified GCN promotes in situ photogeneration of H_2_O_2_ from glucose oxidation. The in situ generated H_2_O_2_ can be used for subsequent TMB peroxidation on the same modified GCN in the dark, demonstrating tandem catalytic activity in glucose detection. Finally, the dual‐functional catalysis was successfully demonstrated in microfluidics for real‐time colorimetric detection of glucose with a low detection limit of 0.8 × 10^‐6^ m within 30 s. The study not only revealed a practical strategy for mimicking the multifunction of natural enzymes, but also an eco‐friendly and low‐toxic nanosensor for biomedical diagnostics.

##### Color‐Changing method

**Figure 10:**
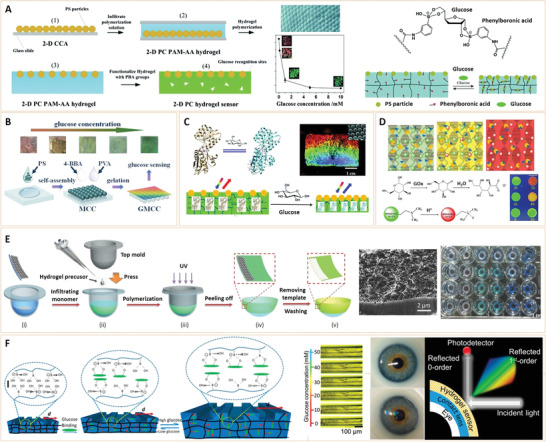
A) Fabrication of a glucose‐responsive 2‐D PC hydrogel and a scheme of the shrinking response for the 2‐D PC hydrogel response to glucose. Reproduced with permission.^[^ ^103^ ^]^ Copyright 2014, The Royal Society of Chemistry. B) Illustration of the construction of gelated monolayered colloidal crystal (GMCCs). Reproduced with permission.^[^ ^104^ ^]^ Copyright 2018, American Chemical Society. C) Venus flytrap conformational change of glucose/galactose binding protein (GGBP) induced by glucose and the bottom of the schematic illustration shows that glucose binding induced 2‐D PC‐GGBP hydrogel VPT causing diffraction blue‐shifts. PC, photonic crystal; VPT, volume phase transition. Reproduced with permission.^[^ ^105^ ^]^ Copyright 2016, The Royal Society of Chemistry. D) Schematic illustrations of the mechanism of glucose detection using the IPN_GOX_ array film before and after glucose addition, and protonation of PDMAEMA by the GOx reaction with glucose. IPN, interpenetrating polymer network; PDMAEMA, poly (2‐dimethylaminoethyl methacrylate). Reproduced with permission.^[^ ^106^ ^]^ Copyright 2020, Elsevier B.V. E) Schematic illustration of the fabrication of a circle structural colored contact lens and photographs and SEM image of structural colored contact lenses. Reproduced with permission.^[^ ^107^ ^]^ Copyright 2014, Wiley‐VCH. F) Left: Effect of glucose−phenylboronic acid complexation in the 1D PS sensor; Middle: Microscopic images of the 1D PS sensor's cross‐section in various glucose concentrations; Right: Contact lens integrated glucose sensor. Reproduced with permission.^[^ ^108^ ^]^ Copyright 2018, American Chemical Society.

In addition to colorimetric assays, analyte‐dependent color‐changing methods can be used to visually identify diabetes‐related biomarkers. Radhakumary et al.^[^ ^101^ ^]^ found that when GOx‐capped gold NPs were exposed to ≈100 µg mL^−1^ glucose, their color changed from red to blue, which was most likely due to the formation of aggregates with enhanced sizes. This work is one of the earliest reports of color‐changing methods for bioanalysis, indicating their great potential for detecting glucose in urine with the naked eye. However, due to the short blue shift in the UV–Vis absorption spectra, this method only exhibits minor color changes, implying a low visual detection accuracy. Responsive photonic crystal (PC) materials with tunable structural colors, on the other hand, have gained increasing attention due to their visual diffraction signals and outstanding response to external stimuli. According to Bragg's law, PC materials possess periodically dielectric structures, which act as selective wavelength filters and diffract specific wavelengths. The obtained PC materials act as PC materials by incorporating analyte‐responsive factors into the PC structure. One of the most extensively used motifs in the fabrication of glucose‐responsive PC materials is Phenylboronic acid (PBA).^[^ ^102^ ^]^ PBA‐modified PC hydrogels, for example, were simply prepared with a layer of 2‐D colloidal crystals on a hydrogel for visual glucose sensing (**Figure** **10**A,B).^[^ ^103^, ^104^ ^]^ These sensors efficiently diffract visible light, which changes its color from red to yellow to green as the glucose concentration changes from 0 × 10^‐3^ to 20 × 10^‐3^ m. Because the range of glucose concentrations encompasses both the physiological glucose levels in tears and blood, these intelligent PC sensing materials offer the potential for frontier point‐of‐care glucose monitoring. A genetically engineered *Escherichia coli* glucose/galactose binding protein (GGBP) could also be used to fabricate a PC hydrogel with highly selective glucose‐sensing properties (Figure 10C).^[^ ^105^ ^]^ The conformational change in GGBP induced by glucose binding could trigger significant volume phase transitions (VPTs), resulting in a visible color change in the PC hydrogels. The blue‐shifted diffractions in the study specifically enabled GGBP‐based PC hydrogel sensors to report on glucose concentrations, and it functioned as a selective and sensitive sensor that easily monitors glucose concentrations ranging from 0.2 × 10^‐6^ to 10 × 10^‐3^ m. For the first time, protein‐ligand binding was used to induce structural color changes in PC hydrogels. In contrast to the relatively low selectivity of PBA‐glucose binding, the innovation in this work may promote the development of highly selective glucose sensors due to the high specificity of protein‐ligand interactions, and can also be used in high‐throughput screening devices that can monitor protein‐drug binding interactions.

In addition to PBA and GGBP glucose binding, the enzymatic interactions of GOx with glucose which causes a reduction in pH was used to trigger a color change in PC hydrogel composed of cationic polyelectrolyte networks (Figure 10D).^[^ ^106^ ^]^ The photonic hydrogel was prepared by intertwining solid‐state cholesteric liquid crystals with cationic polyelectrolyte poly(2‐dimethylaminoethyl methacrylate)  (PDMAEMA). Because PDMAEMA is a typical weak cationic polyelectrolyte that protonates and expands at pH values below its pK_b_, the structural color of this hydrogel revealed a clear pH response in the form of a red‐shift with decreasing pH. The enzymatic reaction of GOx with glucose in the hydrogel results in a reduction in pH value. Thus, the polyelectrolyte photonic hydrogel with GOx immobilization can also be used as a naked‐eye optical sensor for glucose.

Glucose‐responsive PC hydrogels have unique superiority for point‐of‐care glucose detection. Moreover, the plasticity and stretchability of hydrogels make these PC hydrogels suitable for wearable monitoring devices. A class of circular structurally colored contact lenses, for example, was prepared by replicating self‐assembled SiO_2_ colloidal PC templates on 2‐poly(hydroxyethyl methacrylate) (PHEMA) contact lenses (Figure 10E).^[^ ^107^ ^]^ The structural colored contact lenses not only display variable and brilliant colors under light illumination, but they also avoid the addition of any colorants to the hydrogel lenses, thus preventing potential harm posed by traditional colored contact lenses. Taking this work a step further, Elsherif et al.^[^ ^108^ ^]^ introduced PBA groups into a photonic microstructural hydrogel and then used the photonic microstructural hydrogel to fabricate a wearable contact lens optical sensor (Figure 10F). Due to PBA's glucose‐responsiveness, the microstructure volume of PC hydrogel increased upon binding with glucose in tears.^[^ ^109^ ^]^ Changes in periodicity constant and Bragg's diffraction modulated the space between zero‐ and first‐order spots. A correlation was established between the periodicity constant and glucose concentrations ranging from 0 × 10^‐3^ to 50 × 10^‐3^ m. The sensor's sensitivity was determined at 12 nm mM^−1^, and the saturation response time was less than 30 min. When hydrogel was integrated with commercial contact lenses, the resulting contact lens optical sensor could be used for continuous glucose quantification under physiological conditions, equipped with smartphone readouts. In short, this study has made significant progress toward point‐of‐care and continuous glucose monitoring devices, and a PC‐based contact lens optical sensor could be a very essential product in the future for diabetes diagnoses and management at home.

## 3 Nanomaterials for Diabetes Prevention and Therapy

Following a definite diagnosis of diabetes, the next stage is to discover effective treatments for the ailment. Recent studies have explored the potential use of nanomaterials in the treatment of both Type‐I and II diabetes, with particular emphasis on the latter, since approximately 80–90% of diabetes is Type‐II and only 5–10% is Type‐I.^[^ ^110^ ^]^ Type‐II diabetes is caused by insulin that is unable to execute its job adequately. Antidiabetic drugs can be used efficiently to promote insulin secretion or to reduce glucose production. Type‐I diabetes, on the other hand, is completely different in that the pancreas produces very little or no insulin, and treatment with insulin is required for survival. Nonetheless, because of the smart properties of nanoparticles, nanomaterials loaded with natural or synthetic antidiabetic drugs (e.g., insulin, glibenclamide, metformin) improved the clinical outcomes of both Type‐I and ‐II diabetes.

First, because most drugs are poorly absorbed and/or efficiently distributed in the body, they may not reach a systemic concentration high enough to deliver the desired pharmacological effect.^[^ ^111^ ^]^ Metformin and glycyrrhizin (an active ingredient of the roots and rhizomes of *Glycyrrhiza glabra*) have been both shown to have poor/low bioavailability following oral administration due to the slow and incomplete absorption in the gastrointestinal tract.^[^ ^112^, ^113^ ^]^ Nanoparticulate systems have the potential to transform poorly soluble and/or poorly absorbed bioactive molecules into efficiently deliverable drugs. Because of their small size, NPs can enter cells and interact with molecules in the cytoplasm or the genome without causing harm. Controlled drug release is another advantage of nanomaterials for antidiabetic drug loading. Nanomaterials having “ON/OFF” surface properties may provide a hint to regulate the drug release on demand, which is extremely beneficial in terms of improving pharmacological effects and reducing adverse effects. Furthermore, with the development of nanotechnology, many functional nanomaterials with bio‐targeting, photoelectric, photothermal, or antioxidation properties have been reported, which may either improve efficiency or broaden the means for diabetes treatments. In this section, we will discuss recent advances in nano‐assisted diabetes treatment, which use nanomaterials as drug carriers or as independent antidiabetic agents.

### 3.1 Nanomaterials As Carriers

#### 3.1.1 Nanomaterials Loaded with Natural Antidiabetic Extracts

Plants, marine organisms, and microorganisms are abundant in nature, and many of their extracts have therapeutic potential.^[^ ^114^ ^]^ Natural extracts from plants such as *Allium sativum, Eugenia jambolana, Ficus glomerata, Momordica charantia, Murraya koenigii, Catharanthus roseus, Terminalia arjuna*, and *Brassica juncea* have been used to treat diabetes since ancient times.^[^ ^115^ ^]^ As these natural antidiabetic substances are combined with (or loaded into) nanomaterials, excellent antidiabetic effects may be observed, most likely due to increased biocompatibility and decreased toxicity when compared to the use of drugs alone.

Current NPs used for the loading of naturally occurring antidiabetic substances were primarily made of inorganic elements such as Au, Pt, Ag, and ZnO.^[^ ^116^, ^117^ ^]^ Au NPs prepared from the potent anti‐diabetic herb *Cassia auriculata* yielded nano‐Au with average diameters of 15–25 nm. The Au NPs were stable in solutions ranging in pH from 3.4 to 10.2, indicating a high potential for antidiabetic treatment.^[^ ^114^ ^]^ Another Au NPs in Wistar albino rats using the antidiabetic plant *Gymnema sylvestre R. Br*. The Au NPs (≈50 nm, ‐17.5 mV) significantly reduced blood glucose levels in the experimental group (diabetic rats treated with Au NPs, 0.5 mg kg^‐1^ body weight).^[^ ^118^ ^]^ In addition to Au NPs, Pt NPs loaded with *Whitania somnifera* leaf extract (12 nm) generated a significant decrease in plasma glucose levels after administration into streptozotocin‐induced diabetic rats at a dose of 1 mg kg^‐1^.^[^ ^119^ ^]^ Ag NPs (4–25 nm) were also made using an extract from *Solanum nigrum*, a medicinal plant that has historically been used to treat diabetes. The administration of these nanoparticles (10 mg kg^‐1^) to alloxan‐induced diabetic rats resulted in improvements in body weight and dyslipidemia, as well as a decrease in blood glucose levels.^[^ ^120^ ^]^ Similarly, when compared to watercress leaf extract (150 mg dL^‐1^) and insulin (10 U kg^‐1^) therapies, the diabetic status of alloxan‐diabetic Wister rats that received the treatment of enriched ZnO NPs (average pore diameters is 4.8 nm) by watercress (*Nasturtium officinale*) leaf extract at a dosage of 8 mg dL^‐1^ showed the best significant performance.^[^ ^121^ ^]^ These findings suggest that inorganic NPs loaded with extracts of naturally potent antidiabetic plants could be used as potent antidiabetic agents.

#### 3.1.2 Nanomaterials Loaded with Recombinant Human Insulin

Aside from the natural extracts, several synthetic antidiabetic drugs approved by the U.S. Food and Drug Administration (FDA), such as sulfonylureas, biguanides (glucosidase/starch inhibitors), thiazolidinediones (insulin secretagogues), and, in particular, recombinant human insulin, have been used for the control of blood glucose levels in diabetic patients.^[^ ^115^ ^]^ There are two techniques of drug administration; subcutaneous injection and oral delivery of drugs. Small molecular synthetic drugs can be used orally and intravenously. However, because oral insulin administration has very low bioavailability, subcutaneous insulin injection remains the recommended technique for the management of insulin‐requiring diabetic patients.^[^ ^122^ ^]^ It is still a conundrum that insulin administration via subcutaneous injection is delivered to the peripheral circulation rather than to the portal circulation, whereas oral administration can not only alleviate the pain and trauma caused by injections but also mimic the physiological fate of insulin.^[^ ^4^ ^]^ Various nanosystems have been reported in this regard to efficiently increase the oral bioavailability of insulin or other synthetic drugs either by enhancing drug permeability or by overcoming the first‐pass effect.^[^ ^123^ ^]^

Before orally administered insulin can begin working, it must maintain its conformation across the stomach, small intestine, and colon. Along the way, the harsh environment in the gastrointestinal (GI) tract and the acidic environment of the stomach remain significant challenges. As a result, an ideal delivery nano‐system for oral administration of insulin should prolong its intestinal residence time, reversibly increase the permeability of the mucosal epithelium to enhance drug absorption, and improve its stability against GI enzymes and the highly acidic conditions of the stomach to ensure the active fraction of insulin remains intact.^[^ ^124^ ^]^ According to Mukhopadhyay et al.,^[^ ^125^ ^]^ effective delivery systems for oral insulin delivery must meet several criteria, including 1) pH‐sensitivity, the ability to protect drugs at low pH of the stomach and release them at intestinal pH; 2) “site‐specific” drug release, i.e., close to the absorption surface to avoid intestinal proteases; 3) selective and reversible opening of the tight junctions is preferable; 4) the release process should be controlled to attain the physiological insulin concentrations in the blood; and 5) the drug delivery vehicle should be biocompatible. In this regard, different nanomaterials have been produced to address the above issues. pH‐sensitivity and a targeted strategy to improve cellular uptake, for example, were integrated into nanomaterials and developed for drug delivery. Furthermore, several developing nanomaterials with biofeedback mechanisms capable of releasing antidiabetic drugs in the presence of high glucose levels (i.e., glucose‐responsiveness) have recently been reported. These smart nanosystems provide a variety of methods for improving not only oral bioavailability, but also subcutaneous injection of antidiabetic drugs.

##### pH Sensitivity

**Figure 11:**
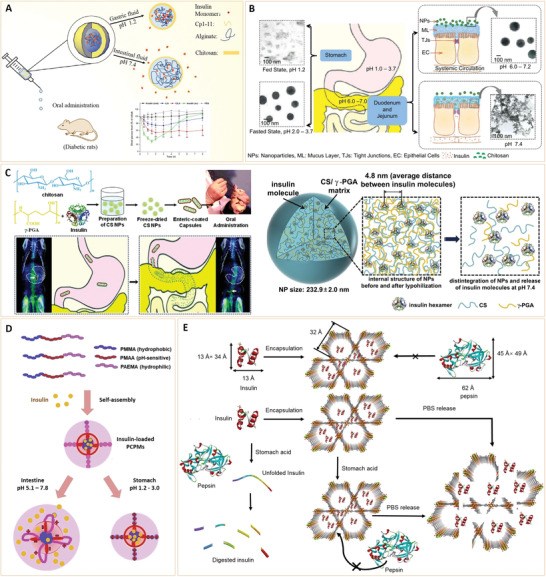
A) Schematic illustration of ALG/CS NPs as a carrier for the delivery of the Cp1‐11 peptide/insulin complex. Reproduced with permission.^[^ ^129^ ^]^ Copyright 2019, Elsevier B.V. B) Schematic illustrations of the presumed mechanism of the paracellular transport of insulin released from test NPs [using the proximal small intestine (duodenum and jejunum) as an example]. Reproduced with permission.^[^ ^131^ ^]^ Copyright 2008, Elsevier B.V. C) Schematic illustrations of the synthesis of enteric‐coated capsules filled with freeze‐dried CS/poly (g‐glutamic acid) NPs (Left); Schematic illustrations of the internal structures of test NPs and their disintegration at pH 7.4 (Right). Reproduced with permission.^[^ ^132^ ^]^ Copyright 2010, Elsevier B.V. D) Schematic illustrations of the smart pH‐responsive polymeric micelles for programmed oral delivery of insulin. Reproduced with permission.^[^ ^133^ ^]^ Copyright 2019, Elsevier B.V. E) Schematic illustration of insulin encapsulation in the NU‐1000 mesopores, exclusion of pepsin from the MOF framework, and exposure of free insulin and insulin@NU‐1000 to stomach acid. Reproduced with permission.^[^ ^134^ ^]^ Copyright 2018, American Chemical Society.

pH‐sensitive nanomaterials were among the first materials to gain widespread attention as nanocarriers for encapsulating insulin and protecting it from degradation. These nanomaterials can be engineered to be stable at low pH levels, such as those found in the stomach, and to release drugs at higher pH levels, such as those found in the intestine.^[^ ^124^, ^126^ ^]^ Alginate (ALG) is an anionic natural linear polysaccharide, that has been widely used as a carrier for oral insulin delivery due to its excellent biodegradability, biocompatibility, pH‐sensitivity, bioadhesion, and complementary function (carboxyl groups) to positively charged insulin. Hydrogels based on ALG swell at pH > 4.5, due to the low p*K*a (≈4.5) value of carboxyl groups, but the swelling is limited at pH < 4.5. Thus, by altering the ambient pH values, the release of encapsulated drugs in ALG‐based materials may be controlled, demonstrating the potential for drug retention in the stomach and protection against enzymatic deactivation.^[^ ^127^ ^]^ Chitosan (CS, a cationic polysaccharide)^[^ ^125^ ^][^ ^128^, ^129^ ^]^ is commonly used to nucleate anionic ALG molecules through electrostatic attraction, to obtain nanosized ALG drug carriers, and the combination of CS and ALG is currently being extensively studied in pharmaceutical research for oral insulin delivery. Mukhopadhyay et al.^[^ ^130^ ^]^ prepared CS/ALG NPs (100–200 nm) by forming an ionotropic pre‐gelation with an ALG core for insulin entrapment. The results showed that almost all of the encapsulated insulin (≈85%) was retained in the simulated gastric buffer, followed by a sustained release in the simulated intestinal environment, indicating excellent pH sensitivity for insulin release in vitro. Similarly, Chen et al.^[^ ^129^ ^]^ used CS/ALG NPs as the carriers to develop an oral insulin delivery system for the oral co‐delivery of Cp1‐11 peptide (C‐peptide, the fragment EAEDLQGVE)/insulin (**Figure** **11**A). The results demonstrated that the particle size of blank ALG/CS nanoparticles was larger than 500 nm; however, the particle size, after loading C‐insulin was 237.2 nm. Furthermore, the supramolecular interaction between insulin and the Cp1‐11 peptide may inhibit insulin aggregation and improve its bioactivity. The reported system efficiently shielded insulin from degradation by simulated gastric fluid (pH 1.2), while also promoting insulin release in simulated intestinal fluid (pH 7.4). Besides, when compared to free insulin‐loaded NPs, the co‐delivery system (at the insulin dose of 50 IU kg^‐1^) appeared to have an excellent hypoglycemic effect and resulted in higher pharmacological availability of insulin in diabetic rats.

Other anionic natural polymers, such as poly‐*γ*‐glutamic acid and poly‐galacturonic acid, have been investigated as potential carriers for pH‐sensitive insulin delivery in addition to ALG.^[^ ^131^, ^132^, ^135^, ^136^ ^]^ It is worth noting that in these studies, CS is commonly used not only to stabilize and form nanosized drug carriers but also to mediate the opening of tight junctions between epithelial cells to improve drug permeability via the paracellular pathway.^[^ ^137^, ^138^ ^]^ Sonaje et al.^[^ ^131^ ^]^ reported self‐assembled pH‐sensitive NPs (218.0 ± 3.4 nm, 25.3 ± 0.9 mV) for oral insulin delivery prepared by combining the poly‐*γ*‐glutamic acid/insulin complex with CS in the presence of MgSO_4_ and sodium tripolyphosphate (Figure 11B). They found that orally administered insulin‐loaded NPs (30.0 IU kg^‐1^) adhered to the mucosal surface in vivo and that the formed components could permeate into the mucosal cell membrane. In this example, orally administered insulin‐loaded NPs exhibited considerable hypoglycemic activity in diabetic rats for at least 10 h, and the relative bioavailability of insulin was reported to be 15.1 ± 0.9%. A further investigation on the bio‐distribution in the same group's rat model revealed that a significant fraction of the insulin‐loaded NPs was retained in the stomach for an extended period following oral delivery, potentially leading to NP disintegration and insulin degradation. To address these problems, the NPs were lyophilized and placed in a gelatin capsule coated with an enteric polymer (Figure 11C).^[^ ^132^ ^]^ The enteric‐coated capsule remained intact in the acidic environment of the stomach but dissolved rapidly in the proximal segment of the small intestine after oral administration. In this case, the insulin‐loaded NPs (232.9 ± 2.0 nm, 26.3 ± 3.4 mV) were efficiently transported into the small intestine, boosting intestinal insulin absorption and a prolonged reduction in blood glucose levels. Furthermore, with the addition of an enteric‐coated capsule, the relative bioavailability of insulin was significantly increased (≈20%). These results revealed that these natural polysaccharide nanoparticles have great potential as pH‐sensitive nano‐vehicles for potential oral insulin delivery.

pH‐sensitive insulin nanocarriers could also be made using synthetic polymers with carboxyl‐acid groups. A series of amphiphilic pH‐sensitive block copolymers, including poly(methyl methacrylate‐*co*‐methacrylic acid)‐*b*‐poly(2‐amino ethyl methacrylate) ((PMMA‐*co*‐PMAA)‐*b*‐PAEMA) was synthesized. The hydrophilic pH‐sensitive PAEMA and hydrophilic pH‐sensitive PMAA allowed it to be self‐assemble into pH‐responsive cationic polymeric micelles (PCPMs) for oral insulin delivery (Figure 11D).^[^ ^133^ ^]^ The PCPMs displayed great stability in an acidic environment and a significant decrease in particle size (from around 500 to 200 nm) with increased pH (6.0 to 7.4). In vitro, the insulin‐loaded PCPMs exhibited low toxicity and pH‐triggered insulin release profile with remitted initial burst release, similar to natural anionic polymers. Furthermore, amphiphilic copolymers of dextran (DEX)‐poly(lactic‐*co*‐glycolic acid) (PLGA) were synthesized and self‐assembled to form polymersomes for insulin encapsulation.^[^ ^139^ ^]^ The in vitro release studies of the DEX5000‐PLGA13000 (F7) and DEX25000‐PLGA48000 (F14) polymersomes both showed negligible insulin release (a maximum of 12.7% (F14) and 17.34% (F7)) in the simulated gastric condition, but a significant increase in insulin release (a maximum of 79% (F14) and 84% (F7)) in the simulated intestinal condition. The strong hypoglycemic effects of insulin‐loaded DEX‐PLGA polymersomes (100‐200 nm size for F7, 200‐300 nm size for F14) in a diabetic rat model showed that these polymersomes could be used as oral insulin carriers.

The results of the preceding studies show that pH‐sensitive polymers have the potential to be used for oral insulin administration. While chemistry and material science have advanced rapidly several new materials with pH‐sensitive properties have also sparked increased interest in oral drug delivery. MOFs are a class of crystalline porous materials made up of metal nodes connected by organic ligands. However, MOFs functioning as a nanocarrier for oral delivery poses a challenge due to their instability in the stomach's aqueous acidic environment. Farha et al. recently showed that immobilizing insulin in zirconium MOFs (NU‐1000) had an acid‐resistant effect (Figure 11E).^[^ ^134^ ^]^ In the presence of stomach acid and the digestive enzyme (e.g., pepsin), acid‐stable MOFs encapsulated with insulin could successfully inhibit insulin degradation. Furthermore, under simulated physiological conditions, the majority of the encapsulated insulin (91%) was released from insulin@NU‐1000. The novel pH‐sensitivity of NU‐1000 demonstrated its great potential as a potential insulin nanocarrier for oral delivery. However, it should be noted that the general application of MOF materials remains a challenge. Aside from smart drug release (i.e., acid resistance), the potential toxicity of metal ions and organic ligands should be well examined. Nevertheless, with the specific requirements of an oral carrier in mind, we predict that novel nanomaterials with high biocompatibility and well‐controlled drug release profiles will be discovered. This would greatly contribute to the present polymer‐based antidiabetic drug delivery systems.

##### Intestinal Permeability

In addition to acid resistance, oral antidiabetic nanocarriers must be able to increase intestinal permeability and overcome mucosal absorption barriers.^[^ ^140^ ^]^ Orally administrated drug carriers adhere to the mucosal layer after traveling through the acidic stomach environment. The released drugs generated as a result of the pH increase must be then transported across the intestinal epithelium via a transcellular or a paracellular pathway, before being secreted into the bloodstream to regulate glucose levels. To improve transcellular and paracellular drug transport, molecular techniques such as ligand‐mediated transcytosis and epithelial tight junctions (TJs) opening have been developed in current oral delivery systems.

##### Ligand‐Mediated Transcytosis

**Figure 12:**
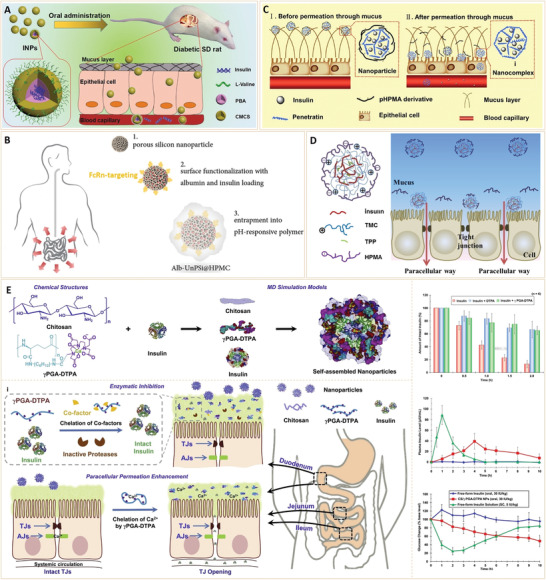
A) Schematic illustration for fabrication of CS‐based multifunctional nanocarriers that overcome multiple barriers for oral insulin delivery. Reproduced with permission.^[^ ^143^ ^]^ Copyright 2016, Elsevier B.V. B) Schematic representation of the engineered multifunctional nanoparticles developed in this study for oral administration, consisting of insulin‐loaded albumin‐functionalized PSi NPs entrapped into HPMC (Alb‐UnPSi@HPMC), for FcRn‐targeted therapy in diabetes. PSi, porous silicon; HPMC, hypromellose acetate succinate; UnPSi, undecylenic acid‐modified thermally hydrocarbonized PSi. Reproduced with permission.^[^ ^147^ ^]^ Copyright 2018, WILEY‐VCH. C) Schematic illustration of NP permeation across the mucus layer and the intracellular transport of NP in epithelial cells. Reproduced with permission.^[^ ^150^ ^]^ Copyright 2015, American Chemical Society. D) Schematic illustration of mucus permeation and tight junction opening by dissociable “mucus‐inert” agent coated trimethyl CS NPs for oral insulin delivery. Reproduced with permission.^[^ ^155^ ^]^ Copyright 2015, Elsevier B.V. E) Schematic diagrams displaying the compositions of CS/*γ*PGA‐DTPA NPs and (i) their mechanisms for enhancing enzymatic inhibition and paracellular permeation. NPs are instantaneously formed via ionic gelation. Reproduced with permission.^[^ ^156^ ^]^ Copyright 2011, Elsevier B.V.

Targeted nanocarriers have been extensively investigated for not only oral delivery but also subcutaneous injection, owing to significant advancements in antibody technology in recent years. The basic concept of ligand‐mediated transcytosis for oral administration is the encapsulation of antidiabetic drugs (e.g., insulin) within a biocompatible nano‐system with modified ligands of receptors that are specifically expressed on the GI tract, particularly the epithelial cell surface. L‐valine is a target ligand that was previously found to aid absorption in the small intestine.^[^ ^141^, ^142^ ^]^ In light of this, Li et al.^[^ ^143^ ^]^ prepared CS‐based multifunctional nanocarriers (190 nm) modified by L‐valine for oral insulin delivery (**Figure** **12**A). The addition of L‐valine to the surface of chitosan‐based nanocarriers extended their gastrointestinal retention period and promotes insulin uptake before elimination from the intestinal canal. Even though the penetration mechanism of L‐valine has not been thoroughly examined at this point, this work suggests that L‐valine modification is a viable strategy to enhance the intestinal permeability of orally administrated nanoparticles. In contrast, a reasonably evident transcytosis mediated by the neonatal Fc receptor (FcRn, which can bind to the Fc domain of immunoglobulin G (IgG) or albumin) has the potential to enhance the bioavailability of orally delivered drugs.^[^ ^144^, ^145^ ^]^ FcRn is expressed on the epithelium. As a result, nanomaterials containing the Fc component of IgG or albumin bind FcRn at the apical surface of epithelial cells.^[^ ^146^ ^]^ In light of this, Martins et al.^[^ ^147^ ^]^ proposed that (FcRn)‐mediated transcytosis be used to enhance insulin transport across the intestinal epithelium (Figure 12B). They created a novel insulin nanocarrier by surface conjugating porous silicon (PSi) NPs with albumin and encapsulating them in pH‐responsive hypromellose acetate succinate (HPMC) via nanoprecipitation. The albumin‐decorated FcRn‐targeted NPs (375 ± 30 nm, ‐33 mV) inhibited drug release and degradation in the harsh stomach environment and demonstrated a pH‐responsive and regulated insulin release profile. In vitro experiment demonstrated that the FcRn‐targeting effect greatly impacts insulin permeation across the intestinal epithelium.

It was also discovered that a synthetic peptide with epithelial cell affinity might be used to enhance oral delivery. For example, a CSK (CSKSSDYQC) peptide identified from a random phage‐peptide library using an in vivo phage display technique was found to have a high affinity for goblet cells, the second largest population of intestinal epithelial cells.^[^ ^148^ ^]^ Because of its goblet cell‐targeting ability, the CSK peptide may aid in the transport of M13 bacteriophage across the intestinal epithelium. CSK‐modified insulin nanoparticles (342.0 ± 5.8 nm) demonstrated improved transportability, resulting in a greater hypoglycemic effect with a 1.5‐fold higher relative bioavailability compared to unmodified insulin nanoparticles.^[^ ^149^ ^]^ This study also suggested that CSK peptide might be used as a potential goblet cell‐targeting ligand for oral delivery of nanocarriers.

Surface modification with cell‐penetrating peptides (CPPs), which have been widely used for systemic delivery of insulin across the intestinal mucosal membrane, is another ligand‐mediated technique to enhance intestinal permeability.^[^ ^150^, ^151^, ^152^, ^153^, ^154^ ^]^ Shan et al.^[^ ^150^ ^]^ reported a self‐assembled NP comprising of a CPP (i.e., penetratin) core and a dissociable hydrophilic poly(*N*‐(2‐hydroxypropyl) methacrylamide) copolymer (pHPMA) layer for efficient oral insulin delivery (Figure 12C). In vitro, the NPs (≈175 nm, ‐1.47 to ‐28.2 mV) showed excellent permeation in mucus due to the “mucus‐inert” pHPMA coating, as well as strong epithelial absorption mediated by CPP. The pHPMA coatings eventually dissociated from the NP's surface in the mucus layer region, and the CPP‐rich core enhanced nanoparticle uptake for subsequent transepithelial transport via the secretory endoplasmic reticulum/Golgi pathway and endocytic recycling pathway. Given this, orally administered NPs (75 IU kg^‐1^) elicited a significant hypoglycemic response, as well as an increase in insulin concentration in the serum of diabetic rats. This study, along with other published oral nanocarriers with CPP modification, demonstrated that CPPs‐enhanced intestinal permeability is an effective approach for improving protein and peptide drugs oral absorption.

It is worth noting that folic acid (FA) could be employed to boost the oral efficiency of drug nanocarriers. As a typical targeting moiety, FA has shown enormous promise in the tumor‐specific delivery of a variety of anticancer agents,^[^ ^157^, ^158^ ^]^ while some studies have embarked on investigating the feasibility of using FA conjugates for improved uptake and transport of poorly absorbed agents (such as proteins and peptides) across the GI tract.^[^ ^159^ ^]^ The intestinal absorption of FA is mediated via a specialized, acidic pH‐dependent carrier‐mediated process. For example, Jain et al.^[^ ^160^ ^]^ reported FA‐decorated polyethylene glycol (PEG)/polylactide‐coglycolide (PLGA) NPs for enhancing insulin oral absorption, while preventing premature protein degradation in the hostile GI tract environment. When compared to subcutaneously administered standard insulin solution, the insulin‐loaded FA‐PEG‐PLGA nanoparticles (size ≈260 nm; insulin loading ≈6.5% [w/w]; encapsulation efficiency: 87.0 ± 1.92%) exhibited a 2‐fold increase in oral bioavailability (double hypoglycemia) without any hypoglycemic shock. The current findings have thus launched a new technique for improving insulin oral delivery using FA‐mediated transport of NP across the gastrointestinal membrane. Similarly, the procedure can be extended to other sensitive oral drugs.^[^ ^161^, ^162^ ^]^

##### Epithelial Tight Junctions (TJs) Opening

In addition to ligand‐mediated transcytosis, the activation of epithelial TJs is another technique for increasing cellular permeability and oral delivery efficacy of antidiabetic nanoparticles.^[^ ^163^, ^164^ ^]^ This technique avoids the difficult intracellular transport of antidiabetic drugs and protects them against enzymatic degradation in the lysosome.

It was previously discovered that CS can adhere to the mucosal surface via TJs between epithelial cells, hence prolonging its residence time in the intestines. NPs based on CS or its derivatives were also discovered to mediate the reversible opening of TJs between epithelial cells, potentially increasing cellular permeability via the paracellular pathway.^[^ ^125^ ^][^ ^165^ ^]^ Initially, the mucoadhesive feature of CS was thought to be a favorable factor for absorption due to the increased retention time and local concentration of the NPs. A recent study, however, found that this feature might limit their access to the epithelial surface since a large proportion of the NPs were trapped in the mucus barrier and eliminated along with the detached mucus. Liu et al.^[^ ^155^ ^]^ proposed a novel self‐assembled NP (163.1 nm) consisting of insulin and trimethyl chitosan (TMC), as well as a dissociable “mucus‐inert” hydrophilic polymer pHPMA, to increase the CS‐mediated TJs opening during oral delivery of antidiabetic drugs (Figure 12D). The mucus‐inert pHPMA on nanoparticles allowed the NP core to reach the epithelial cell surface beneath the mucus. Due to the ability of TMC to transiently open the TJs, the TMC core was then exposed to allow transepithelial transport via the paracellular pathway. This study represents a typical sample of oral nanocarriers capable of overcoming the barriers of both the mucus layer and the epithelium sequentially.

In contrast to the mechanism used by CS for TJs opening, another small molecule diethylene triamine pentaacetic acid (DTPA) can disrupt adherens junctions (AJs) by chelating divalent metal ions (Ca^2+^). Because the formation of AJs has a direct effect on the formation of TJs, it can cause TJs to be indirectly disrupted.^[^ ^166^, ^167^ ^]^ Su et al.^[^ ^156^ ^]^ sought to integrate the TJs opening activities of DTPA in chitosan nanoparticles for oral insulin delivery (Figure 12E). To maintain the complexing agent concentrated on the intestinal mucosal surface, DTPA was covalently conjugated on poly(g‐glutamic acid) (*γ*PGA), and functional nanoparticles were prepared by combining cationic CS with the anionic *γ*PGA‐DTPA conjugate. The prepared nanoparticles (246.6 ± 4.8 nm, 37 ± 0.3 mV) protected insulin from enzymatic degradation by chelating divalent cations (such as Ca^2+^ or Zn^2+^), which are the essential co‐factors for intestinal proteases. The integration of enzyme inhibition and AJs/TJs disruption caused by DTPA promotes paracellular permeation of highly active insulin, thus providing synergy for successful oral insulin delivery. The pharmacodynamic (PD) and pharmacokinetic (PK) profiles revealed that, in addition to causing a prolonged drop in blood glucose levels, oral administration of the enteric‐coated capsule containing CS/*γ*PGA‐DTPA NPs resulted in a maximum insulin concentration at 4 h after treatment. Insulin's relative oral bioavailability was approximately 20%. All of the benefits of the CS/*γ*PGA‐DTPA nanoparticles resulted in a significant and prolonged hypoglycemic effect. As a result, antidiabetic insulin nanocarriers with the potential to modulate TJs/AJs activities may provide a critical additional approach to improve oral insulin absorption and therapeutic efficacy.

##### Glucose Responsiveness

Artificial pancreas‐like, closed‐loop, glucose‐responsive insulin delivery systems that use structural transformations of nanoparticles (e.g., shrinking, swelling, or dissociation) in response to elevated blood glucose levels, could result in insulin release on demand and thus improve its bioavailability.^[^ ^168^, ^169^ ^]^ These glucose‐responsive delivery systems have two mechanisms: 1) glucose‐oxidation‐triggered pH changes using GOx or 2) glucose‐binding‐induced collapse of the delivery system based on concanavalin A (Con A) and phenylboronic acid (PBA).^[^ ^170^ ^]^

**Figure 13:**
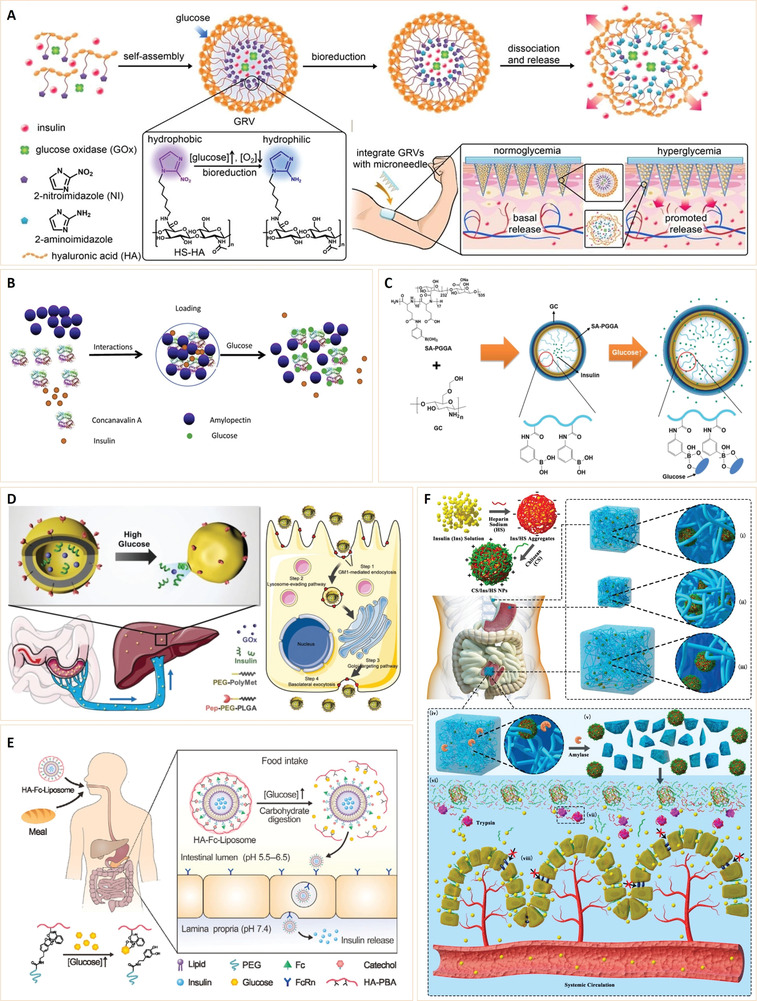
Glucose‐responsive nanoparticles for insulin release. A) Schematic of the glucose‐responsive insulin delivery system using hypoxia‐sensitive vesicle‐loading MN‐array patches. MN, HS, and GRVs represent microneedle, hypoxia‐sensitive, and glucose‐responsive vesicles, respectively. Reproduced with permission.^[^ ^168^ ^]^ Copyright 2015, National Academy of Sciences. B) Schematic synthesis of glucose‐responsive biopolymer NPs based on Con A and amylopectin. Reproduced with permission.^[^ ^176^ ^]^ Copyright 2018, Elsevier B.V. C) Schematic of glucose‐responsive GC/SA‐PGGA double‐layered nanogel formation and controlled insulin release via complexation between PBA derivatives and glucose. GC, SA, and PGGA indicate glycol chitosan, sodium alginate, and poly(L‐glutmate‐co‐N‐3‐L‐glutamylphenylboronic acid), respectively. Reproduced with permission.^[^ ^177^ ^]^ Copyright 2015, The Royal Society of Chemistry. D) Schematic of Pep‐PMS traversing the intestinal epithelium, accumulating in the liver, and releasing insulin in response to elevated blood glucose levels and the GM1 (ganglioside‐monosialic acid)‐mediated transcytosis of Pep‐PMS. GOx, PEG‐PolyMet, and Pep‐PEG‐PLGA represent glucose oxidase, methoxypolyethylene glycol‐polymethionine, and ganglioside GM1‐targeting peptide‐modified PEG‐poly (lactide‐co‐glycolide). Reproduced with permission.^[^ ^178^ ^]^ Copyright 2020, WILEY‐VCH E) Schematic of the glucose‐responsive oral insulin delivery system with glucose‐sensitive HA shell for postprandial glycemic regulation. HA indicates hyaluronic acid. Reproduced with permission.^[^ ^179^ ^]^ Copyright 2018, Tsinghua University Press and Springer‐Verlag, Springer Nature. F) Schematic of the behavior of CS/Ins/HS NPs packaged in CMS‐g‐AA/PMAA hybrid hydrogel during the delivery process in the gastrointestinal tract. Reproduced with permission.^[^ ^180^ ^]^ Copyright 2018, American Chemical Society.

GOx is a well‐studied glucose‐specific enzyme that can catalyze the oxidation of glucose and generate gluconic acid and hydrogen peroxide (H_2_O_2_). The formation of gluconic acid lowers pH levels, which may trigger the swelling or shrinking of pH‐sensitive polymer chains, and as a result, cause a change in their morphology. Because of the glucose‐triggered acidification, GOx has been widely used in diabetes therapy, including insulin delivery and glucose detection areas.^[^ ^171^, ^172^, ^173^ ^]^ Typical GOx‐based glucose‐responsive systems are made of pH‐sensitive carriers with GOx encapsulation such as hydrogels and liposomes. Podual et al.^[^ ^174^ ^]^ prepared a poly(diethylaminoethyl methacrylate‐g‐ethylene glycol) hydrogel immobilized with GOx. In response to the changes in pH (3.0‐7.4), the hydrogel demonstrated rapid swelling/shrinking dynamics. However, the efficiency is limited by a slow response to changes in blood glucose, particularly in a buffered physiological environment. In contrast, due to the enzymatic consumption of oxygen, enclosed nano‐/microsystems rapidly establish a local hypoxic microenvironment. Gu et al.^[^ ^168^ ^]^ developed a novel glucose‐responsive insulin delivery system by combining a microneedle‐array patch with insulin and GOx co‐loaded nanovesicles (GRVs; ≈118 nm). In a hyperglycemic state, the oxidation of glucose can occur in a local hypoxic microenvironment, which leads to the reduction of hypoxia‐sensitive 2‐nitroimidazole (NI) into 2‐aminoimidazoles in the hyaluronic acid (HA) scaffold. The dissociation of a self‐assembled nanosystem and subsequent release of insulin will occur when the hydrophilicity in the core of HA vesicles increases significantly (**Figure** **13**A). The rapid responsiveness of this painless (i.e., microneedle‐assisted drug administration^[^ ^175^ ^]^) insulin patch using hypoxia as a trigger holds promise in the treatment of hyperglycemia and hypoglycemia.

Besides GOx, a saccharide‐binding protein (concanavalin A, Con A) in the lectin family derived from the jack bean plant, also has high specificity and affinity to glucose and mannose. Con A binds two metal atoms (Ca^2+^, Mn^2+^) near the sugar‐binding site in its natural state. The active Con A can then associate with three other active Con A proteins, forming a tetramer aggregate. Each Con A protein in the aggregate can bind to a single glucose molecule.^[^ ^181^ ^]^ Therefore, Con A tetramer aggregates have been used to design glucose‐responsive insulin release.^[^ ^182^, ^183^, ^184^, ^185^ ^]^ For instance, Chang et al.^[^ ^176^ ^]^ developed a new glucose‐responsive nanosystem by assembling Con A and amylopectin (Figure 13B). They loaded insulin into Con A‐amylopectin‐co‐assembled NPs (100–300 nm) in situ. Con A has a higher affinity to glucose than to glycosylated moieties of amylopectin. Therefore, the increased glucose can competitively bind to Con A and substitute amylopectin in the nanoparticles, breaking the bond with nano‐structure and releasing capsulated insulin. The insulin‐release rate could increase by 2.23 times in 3 mg mL^−1^ glucose medium compared with medium without glucose, indicating the glucose‐responsive behaviors of the nanosystem.

Similarly, phenylboronic acid (PBA) also has strong glucose affinity and^[^ ^82^ ^]^ is a widely used non‐biogenic and glucose‐responsive molecule. PBA can change between the uncharged trigonal form (hydrophobic) and the charged tetrahedral form (hydrophilic) under equilibrium.^[^ ^186^ ^]^ PBA‐mediated glucose‐sensitive drug delivery system is mainly based on the reversible reaction between the two states of PBA moieties bound with the cis‐diol of saccharides.^[^ ^187^ ^]^ In recent years, PBA‐functionalized nanomaterials have attracted a lot of attention. For instance, Zhang et al.^[^ ^188^ ^]^ synthesized monodispersed glucose‐sensitive poly(*N*‐isopropylacrylamide) microgels (131 nm) using the PBA group as the glucose‐responsive moiety to develop a self‐regulated insulin delivery system. Furthermore, the PBA‐derivatives converted to hydrophilic moieties with an increased glucose level are crucial in controlled insulin delivery systems due to their stability and biocompatibility. Lee et al.^[^ ^177^ ^]^ also developed a glycol chitosan (GC)/sodium alginate (SA)‐poly(L‐glutmate‐*co*‐*N*‐3‐L‐glutamylphenylboronic acid) (PGGA) grafted polymer nanogel (≈240 nm, +5 mV) using PBA derivatives. The glucose molecules could bind to the boron atoms of the PBA derivative, converting the PGGA chains into hydrophilic structures, leading to swelling and insulin release (Figure 13C). PBA‐based drug delivery nanosystems are mainly based on the glucose‐triggered change from hydrophobicity to hydrophilicity.

PBA, Con A, and GOx are glucose‐sensitive and can be used to develop glucose‐responsive nanosystems. However, clinical trials rarely assess pH‐responsive GOx‐based glucose‐responsive systems, probably due to potential immunogenicity and slow responsiveness (slow swelling and disassociation) at the physiological pH in vivo.^[^ ^170^ ^]^ Efficient interaction between glucose and PBA (Con A) in PBA or Con A‐based glucose‐responsive systems, with a subsequent structural change in the matrix, requires a higher pH value than that in the physiological environment. Besides, the safety and toxicity of PBA conjugates are unknown. Also, the in vivo toxicity and instability of Con A limits its clinical applications.^[^ ^168^ ^]^ Nevertheless, all these glucose‐responsive insulin delivery systems have great potential in diabetes treatment. However, NP formulations using glucose‐responsive sensors can improve the efficiency of the insulin release system to some extent. They can be engineered to detect environmental fluctuations in blood glucose levels and respond by releasing antidiabetic factors through material degradation, disassembly, or swelling.^[^ ^189^ ^]^ Therefore, robust glucose‐responsive NPs have high clinical potential for nanotechnology‐based diabetes therapy.

##### Multifunctionality

Multifunctional or multiple stimuli‐responsive nanomaterials have been designed to treat diabetes since the responsiveness towards biological stimulus may not produce on‐time and efficient insulin release. Wang et al.^[^ ^178^ ^]^ recently showed that oral insulin systems that target the liver and mimic the glucose‐responsive secretion could treat the glucose metabolism defects caused by peripheral delivery. They designed smart polymersomes (Pep‐PMS) composed of glucose‐responsive polymers with peptides that readily bind to the ganglioside‐monosialic acid (GM1) receptor in the intestinal epithelium. The Pep‐PMS (154 nm, +5.22mV) can be efficiently transported across the intestinal epithelium through GM1‐mediated transcytosis, increasing its accumulation in the liver. Moreover, they efficiently released insulin in a glucose‐responsive manner (Figure 13D). Yu et al.^[^ ^179^ ^]^ also reported a glucose‐responsive oral insulin delivery system based on Fc receptor (FcRn)‐targeted liposomes with glucose‐sensitive HA shell (94 nm, ‐28.1 mV) for postprandial glycemic regulation (Figure 13E). Besides releasing insulin in hyperglycemia, the exposed Fc groups on the surface of liposomes enhance intestinal absorption in an FcRn‐mediated transport pathway. In vivo studies (diabetic mice treated with HA‐Fc‐Liposomes and insulin dose at 10 U kg^−1^) confirmed that the delivery approach can effectively reduce postprandial blood glucose excursions. Multiple stimuli‐responsive nanosystems also enhance the sensitivity of responsiveness for the delivery of diabetic drugs. Liu et al.^[^ ^180^ ^]^ developed a pH and amylase dual‐responsive hydrogel to improve the acid‐stability of insulin‐loaded NPs and enhance intestine‐targeted delivery (Figure 13F). The hydrogel had great pH sensitivity in the gastrointestinal tract and rapid enteric behavior under intestinal amylase. Therefore, the insulin release from the NP‐incorporated hydrogel (422.9 ± 5.3 nm) is efficiently accelerated in artificial intestinal fluid, especially in the presence of amylase. These novel platforms based on multiple stimuli‐responsive NPs or molecular targeting mechanisms might be a promising oral vehicle for enhanced insulin delivery, thus providing a better therapeutic effect than some monofunctional nanosystems.

#### 3.1.3 Nanomaterials Loaded with Other Antidiabetic Drugs

The above studies confirmed that insulin‐loaded NPs may be customized to improve insulin delivery. However, NPs can also improve the bioavailability of other types of antidiabetic drugs in vivo. For instance, stevioside, a non‐toxic and FDA‐approved sweetener, has outstanding antidiabetic potential. However, its therapeutic use is restricted in humans due to its poor intestinal absorption and bioavailability. Barwal et al.^[^ ^115^ ^]^ conjugated biodegradable Pluronic‐F‐68 copolymer‐based poly (lactic acid) NPs using stevioside via the nano‐precipitation method to enhance intestinal absorption and bioavailability. The resultant spherical NPs (110–130 nm) showed improved stevioside delivery. Similarly, metformin is poorly absorbed in the gastrointestinal tract (oral bioavailability; 50–60%). Mirazi et al.^[^ ^113^ ^]^ developed metformin‐conjugated nanotubes to reduce blood glucose levels, prolong drug delivery, and enhance efficacy in diabetic treatment compared with metformin alone (150 mg kg^‐1^, orally (p.o.)). Glibenclamide is also commonly used to treat Type II diabetes patients. Glibenclamide dissolution in a physiological medium is the rate‐limiting step for its absorption. A recent study showed that zinc NPs loaded with glibenclamide (196 nm) can induce a significant hypolipidemic effect in *Caenorhabditis elegans* worms, reducing about 15% of fat content. However, the NPs were not significantly modified, suggesting that zinc NPs loaded with glibenclamide can treat diabetes.^[^ ^190^ ^]^ Besides, glucagon‐like peptide‐1 (GLP‐1) is an incretin hormone that can treat Type II diabetes. However, the harsh conditions in the gastrointestinal tract and poor bioavailability limit the oral administration of GLP‐1.^[^ ^191^ ^]^ Martins et al.^[^ ^192^ ^]^ developed a nanosystem for GLP‐1delivery. They functionalized undecylenic acid‐modified thermally hydrocarbonized porous silicon (UnPSi) nanoparticles (NPs) using the Fc fragment of immunoglobulin G and coated it with mucoadhesive chitosan for targeting applications. GLP‐1 was then loaded into the NPs, enhancing GLP‐1 absorption through the intestinal monolayers and thus demonstrating the FcRn transcytotic capacity. Therefore, this nanosystem can be used for the oral delivery of GLP‐1. Moreover, exendin‐4, a glucagon‐like peptide‐1 (GLP‐1) mimetic, can be used to treat Type II diabetes. However, it is limited by the low patient compliance since it requires frequent injections. Ahn et al.^[^ ^193^ ^]^ found that the conjugation between the low molecular weight chitosan (LMWC) and cysteinylated exendin‐4 can maximize the availability of the active peptide. A pharmacokinetic study showed that the bioavailability of LMWC‐exendin‐4 conjugate (101 ± 41 nm) after oral administration (400 µg kg^‐1^) is 6.4% compared with the subcutaneous administration. Besides, the absorbed exendin‐4 showed a significantly enhanced hypoglycemic effect. Therefore, LMWC‐exendin‐4 conjugate is a potential oral antidiabetic agent for the treatment of Type‐II diabetes. Furthermore, Araújo et al.^[^ ^194^ ^]^ developed a multifunctional composite nanosystem using PLGA nanoparticles through the droplet microfluidics technique for dual delivery of GLP‐1 and dipeptidyl peptidase‐4 inhibitor (iDPP4) in vivo. The PLGA nanoparticles in this system were functionalized with chitosan (CS) and a cell‐penetrating peptide (CPP). The plasmatic insulin levels increased after 6 h of the oral administration of the system (the amount of administered particles was equivalent to a GLP‐1 content of 200 µg kg^‐1^ of weight of the rat). Moreover, the blood glucose level was significantly lower than in the control group. Therefore, NPs in delivery systems are a promising approach for the development of new oral formulations of antidiabetic agents.

#### 3.1.4 Nanomaterials Loaded with Islet

Unlike medication therapy, islet transplantation has recently become a promising, long‐term, compliance‐free, and complication‐preventing treatment for type I diabetes. Islet cells are mainly transplanted into the liver via portal vein injection since it is noninvasive and requires fewer islet cells than other organs. However, a nonspecific inflammatory and thrombotic reaction (instant blood‐mediated inflammatory reaction, IBMIR) may occur when islet cells are exposed to the recipient blood, causing substantial destruction of transplanted islet cells.^[^ ^195^ ^]^ However, encapsulating cells in an immunoisolating membrane can enhance their transplantation without the need for immunosuppression. So far, a large number of studies related to islet transplantation have focused on the encapsulation strategies.^[^ ^196^, ^197^ ^]^

Coating islets with nanosized polymer films has been burgeoned as an islet protection modality.^[^ ^198^ ^]^ For example, Krol et al.^[^ ^197^ ^]^ reported a novel approach for immune protection of human pancreatic islets via multilayer nanoencapsulation. In their study, trypan blue‐stained human islets were observed after encapsulation with three layers of polycation and polyanion pairs. The encapsulation with this nanosystem was shown to be a possible alternative to the more space‐consuming and random islet‐trapping microencapsulation after several analyses (release of insulin, fluorescence microscopy, etc.). In fact, the approach in the paper of Krol et al. may also be called a “layer‐by‐layer” (LBL) approach as it encapsulates the islets or other types of cell aggregates by altering positively and negatively charged polymers over the surface of the cell aggregate. Primary reasons for developing this approach were the reality of more standard cell aggregate hydrogel coatings that can leave openings in the coatings, and the diffusional considerations of hydrogel nanocapsules that can decrease encapsulated islet responsiveness to glucose changes. Moreover, it can significantly minimize the capsule thickness, increasing the rate of insulin release in response to glucose as well as promote enhanced diffusion of nutrients and waste products.^[^ ^199^ ^]^

Besides, downregulation of factors that mediate immune rejection using RNA interference holds potential for improving islet graft resistance to damaging factors after transplantation. Based on this theory, Wang et al.^[^ ^200^ ^]^ prepared a dual‐purpose therapy/imaging small interfering (si)RNA magnetic nanoparticle (MN) probe that targets b2 microglobulin (B2M), a key component of the major histocompatibility class I complex (MHC I). Besides being a siRNA carrier, MN‐siB2M probe can enhance monitoring of graft persistence noninvasively via magnetic resonance imaging (MRI). A study labeled human islets with these MNs before transplantation into B2M (null) NOD/scid mice. The MNs significantly improved preservation of graft volume after two weeks, based on longitudinal MRI in an adoptive transfer model. Importantly, mice transplanted with MN‐siB2M‐labeled islets demonstrated a significant delay in diabetes onset (up to 23.8 ± 4.8 d) after the adoptive transfer of T cells compared with the controls (6.5 ± 4.5 d). Therefore, this islet delivery nanosystem can protect pancreatic islet grafts from immune rejection and be used for allotransplantation and prevention of the autoimmune recurrence of T1DM in islet transplantation or endogenous islets.

Recently studies found that functionalized biogenic scaffolds with biocompatible 3D structure can provide efficient solutions for islet transplantation. Bioscaffolds contain spaces that can accommodate islets, while concurrently offering a unique interface which can be modulated to address critical shortcomings faced by islets in the immediate post‐transplantation period (i.e., hypoxia).^[^ ^201^, ^202^ ^]^ Razavi et al.^[^ ^203^ ^]^ prepared a collagen‐based cryogel bio‐scaffold to accommodate pancreatic islets, and added incorporates calcium peroxide (CPO) into its matrix to prevent hypoxia‐induced damage following transplantation. The in vivo study showed that when syngeneic islets are transplanted into the epididymal fat pad (EFP) of diabetic mice, the cryogel‐0.25 wt% CPO bioscaffold improved the islet function with diabetic animals re‐establishing glycemic control. Mice transplanted with cryogel‐0.25 wt% CPO bio‐scaffolds showed faster responses to intraperitoneal glucose injections and had a higher level of insulin content in their EFP compared to those transplanted with islets alone. The results indicated that the cryogel‐0.25 wt% CPO bio‐scaffold could provide a biostable and biocompatible 3D microenvironment for islets, which facilitated islet survival and function at extra‐hepatic sites of transplantation.

Overall, nanomaterials‐based encapsulation of islets can be an effective means for the treatment of diabetes. Whereas current strategies are all double‐edged, further efforts should be made for the limited nutrient transport, the requirement of large injection volumes, and poor stability. Hence, in order to provide a conformal and stable coating of islets, it is important to develop new strategies for design of new multifunctional coatings with immunomodulatory capabilities, which can maintain the physiological needs of the islets.

### 3.2 Special Functional Nanomaterials

Continuous delivery of antidiabetic drugs to the circulation is necessary for drug‐loaded nanomaterials for efficient diabetes treatment. The delivery system is better than the annoying daily insulin injection or other medications, increasing patient compliance. Some emerging functional nanoparticles have also shown unique advantages.

#### 3.2.1 Antioxidant Nanomaterials

**Figure 14:**
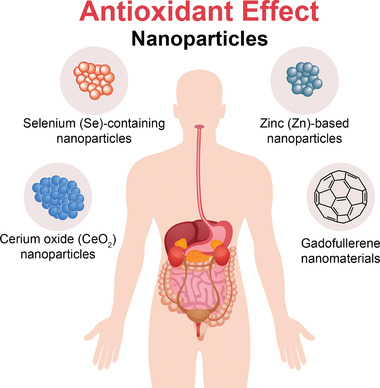
Schematic of antioxidative NPs.

Several studies have shown that cellular redox (redox) imbalance can regulate certain signaling pathways related to *β*‐cell dysfunction and insulin resistance, resulting in oxidative stress and the occurrence and development of diabetes and related complications.^[^ ^204^, ^205^ ^]^ Nanomaterials with antioxidant effect have been extensively studied, identifying several typical antioxidant nanomaterials with potential for diabetes treatment (**Figure** **14**).

For instance, selenium (Se)‐containing NPs can alleviate hyperglycemia and hyperlipidemia in streptozotocin‐induced diabetic rats.^[^ ^206^, ^207^ ^]^ Se plays a critical role in the antidiabetic activity of these NPs. Se is an essential trace element in humans and can efficiently ameliorate diabetes‐induced biochemical changes, oxidative stress, histological and subcellular injury, DNA damages, apoptosis, altered protein expressions of Nrf‐2, catalase, glutathione peroxidase 4 (GPX4) in rats.^[^ ^208^ ^]^ Besides, Selenate exerts several insulin‐like effects in rat adipocytes by stimulating glucose transport activity, cAMP phosphodiesterase activity, and ribosomal S6 protein phosphorylation. ^[^ ^209^ ^]^ Treatment of streptozocin‐induced diabetic rats with sodium selenate (10‐15 µmol kg^−1^ day^−1^) for seven weeks can decrease plasma glucose, food intake, and water intake to normal or near the normal levels, similar to the effect of insulin.^[^ ^210^ ^]^ However, Se functions are extremely restrained due to the narrow margin between its functionality and toxicity.^[^ ^211^ ^]^ Nevertheless, Se toxicity is greatly reduced when in a nanometer form, enhancing or maintaining its activity. Se NPs have attracted a lot of attention due to their unique biological activities and low toxicity. Besides, Se NPs play a role of versatile carrier for oral delivery of insulin, jointly potentiating the antidiabetic effect.^[^ ^212^ ^]^ Therefore, Se NPs may be a new antidiabetic drug substitute in the future.

The rare earth cerium oxide (CeO_2_) NPs also has unique antidiabetic property. Abdollahi et al.^[^ ^213^ ^]^ combined CeO_2_ NPs (60 mg kg^‐1^ d^‐1^) and sodium selenium (Na_2_SeO_3_, 5 µmol kg^−1^ day^−1^) for the treatment of diabetic rats to assess the antioxidant potential of CeO2 NPs. They aimed to show that increased oxidative stress promotes the development and progression of diabetes and its complications. The treated rats had a significant increase in weight and a decrease in blood glucose. The treated rats also showed significantly improved indexes of diabetic biomarkers, such as oxidative stress, energy compensation (ADP/ATP), lipid profile, and hepatic ROS levels, compared with the control rats. They also found that the combination of CeO_2_ NPs and Na_2_SeO_3_ can improve pancreatic islet function during isolation and transplantation procedures.^[^ ^214^ ^]^ These results indicate that the elimination of diabetes‐induced oxidative stress using antioxidant CeO_2_ NP can enhance diabetes treatment.

**Figure 15:**
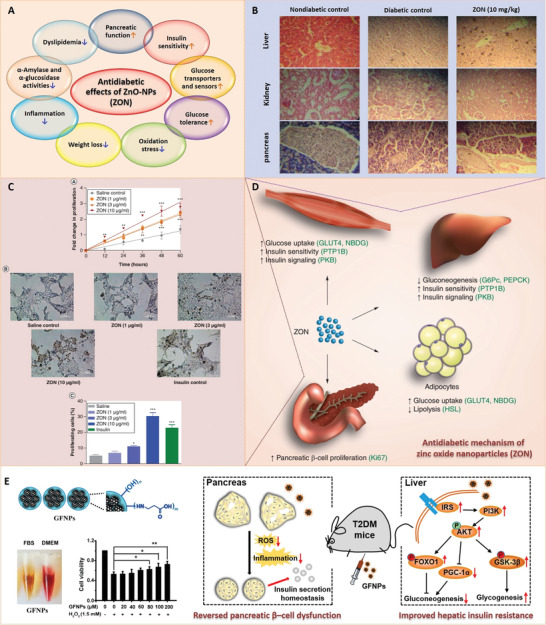
A) Antidiabetic effects of ZON. Reproduced with permission.^[^ ^215^ ^]^ Copyright 2019, Elsevier B.V. B) Photomicrographs (magnification 200 ×) of hematoxylin‐eosin‐stained sections of liver and kidney of non‐diabetic control, diabetic control, and ZON‐treated (10 mg kg^−1^) groups. ZON was administered to Type II diabetic rats once daily for weeks. Reproduced with permission.^[^ ^217^ ^]^ Copyright 2013, Future Medicine Ltd. C) Effect of ZON on pancreatic beta‐cell proliferation. (A) Cell viability assay. RIN5f cells were exposed to different ZON concentrations o(1, 3, 10 µg mL^−1^) at various time intervals. Cell viability was assessed using a trypan blue dye exclusion assay. Fold change in proliferation was calculated. (B) Ki‐67 immunocytochemistry of RIN5f cells. Cells were treated with ZON (1, 3, 10 µg mL^−1^) or insulin for 24 h. The cells were immunostained for Ki‐67, a cellular proliferation marker. (C) Morphometric analysis of proliferating cells (Ki‐67 positive). D) Proposed mechanism of antidiabetic action of ZON. Reproduced with permission.^[^ ^218^ ^]^ Copyright 2016, Future Medicine Ltd. E) Characterization of GFNPs in vitro and the biodistribution study of GFNPs in vivo and the proposed mechanism of the treatment of Type II diabetes via GFNPs. GFNPs, amino acid‐functionalized gadofullerene nanoparticles. Reproduced with permission.^[^ ^219^ ^]^ Copyright 2019, American Chemical Society.

Besides Se and Ce, zinc (Zn)‐based NPs also have the potential for antidiabetic applications. Zn is crucial in insulin synthesis, storage, and secretion. Therefore, researchers have investigated the antidiabetic effects of Zn‐containing NPs, confirming their potential antidiabetic applications (**Figure** **15**A).^[^ ^215^ ^]^ Gharbawy et al.^[^ ^216^ ^]^ demonstrated that zinc oxide NPs (ZON, ≈20 nm) alone have significant antidiabetic effects since they can restore the function and structure of beta cells and improve many of the indices of diabetic dysfunction (glucose tolerance, weight loss, insulin levels, fructosamine levels, pancreatic superoxide dismutase activity, and pancreatic histology). Furthermore, ZON (1, 3 and 10 mg kg^‐1^ d^‐1^ p.o.) and Vildagliptin (10 mg kg^‐1^ d^‐1^ p.o.) alone or in combination can significantly decrease microRNA‐103 and microRNA‐143 expression in a streptozotocin‐induced rat model of Type‐II diabetes compared with the diabetic group, indicating a similar that ZON and the oral anti‐hyperglycemic agent Vildagliptin have similar effects. Therefore, ZON supplementation in the prediabetic stage may prevent progression to Type‐II diabetes. Umrani and Paknikar.^[^ ^217^ ^]^ also found that ZON treatment (1, 3, and 10 mg kg^‐1^ d^‐1^ p.o.) increases serum superoxide dismutase (SOD) and catalase activities, indicating the antioxidant effects of ZON. Also, ZON treatment only treats the zinc deficiency in diabetes but does not result in any histological changes in liver and kidney tissue, necessitating further development of ZON as an antidiabetic agent (Figure 15B). In a follow‐up study, they also found that ZON has pleiotropic antidiabetic effects, such as improving insulin signaling, enhancing glucose uptake, decreasing hepatic glucose output, reducing lipolysis, and enhancing pancreatic beta‐cell mass (Figure 15C,D). ^[^ ^218^ ^]^ The results suggest that ZON acts as an insulinomimetic to activate PKB, translocate GLUT4, and increase glucose uptake. Furthermore, ZON acts as an insulin‐sensitizing agent, showing beneficial effects on glucose metabolism by inhibiting gluconeogenesis. ZON may also induce favorable effects in diabetes‐associated dyslipidemia via lipolysis inhibition. These positive functions, together with the enhancement of pancreatic beta‐cell proliferation suggest the pleiotropic antidiabetic effects of ZON.

In addition to metal oxide, fullerene and gadofullerene nanomaterials are potential antidiabetic agents due to their excellent scavenging abilities on oxygen radical species (ROS).^[^ ^219^ ^]^ Li et al. synthesized amino‐acid‐functionalized gadofullerene NPs (GFNPs, 137.5 ± 2.21 nm, −36.5 ± 1.70 mV) to assess their potential in Type‐II diabetes mellitus treatment (Figure 15E). They found that GFNPs positively regulate the IRS/ PI3K/AKT insulin signal pathway, ameliorating hepatic insulin resistance in a diabetic mice model. The GFNPs also improved glucose metabolism by inhibiting hepatic gluconeogenesis and promoting hepatic glycogenesis through AKT/FOXO1/PGC‐1*α* and AKT/GSK‐3*β* signal pathways. Therefore, the antioxidant GFNPs can be used as anti‐diabetic drugs. Therefore, functional NPs or their derivatives that eliminate oxidative stress in the body can become new alternatives to hypoglycemic drugs in the near future.

#### 3.2.2 Sugar Storing/Releasing Nanomaterials

**Figure 16:**
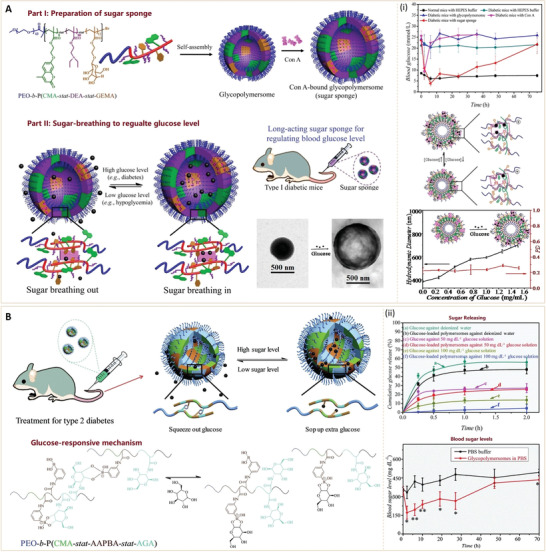
A) Preparation of a Con A bound glycopolymersome (sugar sponge) and its blood glucose regulation behavior via sugar breathing. The sugar sponges could bind/release glucose at high/low glucose concentrations, respectively. The sugar sponges can enhance long circulation for regulating blood glucose levels (i). Reproduced with permission.^[^ ^220^ ^]^ Copyright 2017, American Chemical Society. B) Blood glucose regulation behavior of glycopolymersomes. (ii) Antidiabetic tests of PBS buffer and KM mice treated with glycopolymersomes (4.0 mg kg^−1^). Reproduced with permission.^[^ ^221^ ^]^ Copyright 2019, The Royal Society of Chemistry. Con A, PEO‐b‐P‐(CMA‐stat‐DEA‐stat‐GEMA),HEPES, and PEO‐b‐P(CMA‐stat‐AAPBA‐stat‐AGA) represent Concanavalin A, poly(ethylene oxide)‐block‐poly[(7‐(2‐methacryloyloxyethoxy)‐4‐methylcoumarin)‐stat‐2‐(diethylamino)ethyl methacrylate‐stat‐(*α*‐d‐glucopyranosyl)ethyl methacrylate], 4‐(2‐hydroxyethyl)‐1‐piperazineethanesulfonic acid, and poly(ethylene oxide)‐block‐poly[(7‐(2‐methacryloyloxyethoxy)‐4‐methylcoumarin)‐stat‐3‐acrylamidophenylboronic acid‐stat‐N‐acryloyl glucosamine], respectively.

Unlike the biological mechanisms of antioxidant NPs, Xiao et al.^[^ ^220^, ^221^ ^]^ recently investigated a new nanosized “sugar sponge” for glucose control via a chemical mechanism. The novel nanomaterials, glycopolymersomes, were self‐assembled from sugar derivative‐containing copolymer with bound lectins Con A (**Figure** **16**A) or PBA moieties (Figure 16B). The Con A or PBA moieties could dynamically bind its glucosyl moiety and glucose, developing glucose storage in glycopolymersomes by reversibly substituting sugar. In addition, the Con A or PBA moieties could actively store excess glucose, releasing it gradually as the body requires it. Although the glycopolymersome does not involve insulin or anti‐diabetic drugs, it reversibly stops and removes glucose in the bloodstream, exhibiting a long‐acting regulation of blood sugar levels. The glycopolymersome NPs tend to bind glucose in the bloodstream by substituting the glucosyl on the polymer chain when the glucose concentration is high, leading to swelling of the glycopolymersomes. In contrast, the glycopolymersomes will shrink when the glucose level is low and subsequently releasing the bound glucose.^[^ ^220^ ^]^ However, lectin degradation in these two conjugates may limit its long‐term therapy in vivo since the sugar‐breathing behavior of this sugar sponge relies on the glucose responsiveness of the lectin.^[^ ^222^ ^]^ Furthermore, PBA groups can be used as the glucose‐responsive component since they can reversibly bind biologically important 1,2‐ and 1,3‐diols.^[^ ^223^ ^]^ Therefore, these two are useful for regulating blood glucose levels in Type I‐ and II‐diabetes, respectively. Overall, the new mechanism of “controlling sugar levels with sugar” provides insights into regulating the blood glucose level without insulin or other antidiabetic drugs. Wu et al. also reported a non‐hormonal hyperglycemia treatment strategy using an injectable glucose‐responsive boronic acid‐zwitterionic nanogel.^[^ ^224^ ^]^ The glucose‐binding PBA moieties were rationally tuned for glucose storage or release at high or low blood glucose concentrations, respectively. The optimized nanogels (≈400 nm) demonstrated excellent reversible glucose‐responsive volume change and a desirable profile for glucose regulation in vitro, indicating that this nanogel could act as an artificial nano‐liver. In vivo results have also further confirmed the excellent glucose‐regulating effect in Type‐II diabetes rats for at least 6 h after a single dose of nanogel injection. The above results warrant further development of a novel non‐hormonal polymeric glucose regulation system for diabetes treatment with severe insulin resistance.

## 4 Nanomaterials for Treatment of Diabetes Complications

High levels of intracellular glucose can increase ROS production, altering several critical downstream pathways that may lead to the development of diabetes complications.^[^ ^225^ ^]^ However, the development of diabetes complications cannot be predicted based on clinical risk factors and glycemic control alone. Diabetes is associated with long‐term damage to both large and small blood vessels in the body (macrovascular and microvascular systems, respectively). Although hyperglycemia‐induced damage to the macrovascular system is the leading cause of death in individuals with diabetes, hyperglycemia‐induced damage to the microvascular network in the retinopathy, nephropathy, and neuropathy is the most common and has a high mortality. Moreover, diabetic cardiomyopathy and delayed wound healing severely reduce the quality of life in individuals with diabetes.^[^ ^226^, ^227^ ^]^

### 4.1 Nanomaterials in Diabetic Retinopathy

#### 4.1.1 Nanoparticles as Drug Carriers

**Figure 17:**
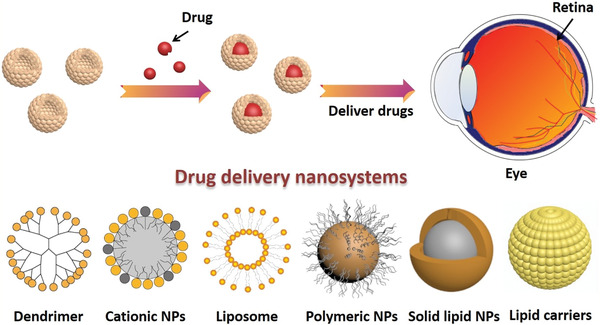
NP‐based drug delivery systems used to reach the posterior segment of the eye.

Long‐term hyperglycemia can cause several metabolic and physiologic abnormalities in the retina, greatly enhancing the recognized features of diabetic retinopathy (DR).^[^ ^228^ ^]^ DR is the leading cause of visual impairment and blindness, and it is mainly characterized by microvascular damage, swelling of blood vessels, and fluid leak. DR prevalence is increasing due to the increasing number and prolonged survival of diabetic patients. ^[^ ^229^ ^]^ Besides, the growth of new vessels if the DR is not well treated can lead to various complications, such as vitreous hemorrhage, retinal detachment, and glaucoma. Currently, retinopathy is the research focus of nanomaterial‐based medicine in ophthalmology. However, the drug molecules cannot reach the retina in therapeutic concentration due to the restricted permeability caused by blood‐retinal barriers.^[^ ^230^ ^]^ Besides controlling glucose levels, functional nanomaterials are also promising for DR treatment. As novel drug delivery systems, nanomaterials can overcome the natural barriers of the eye, including the cornea, conjunctiva, and blood‐retinal barriers. To date, various functional NPs loaded with drugs have been developed for DR treatment. These drug delivery nanosystems, with different formats, such as the liposomes, cationic nano‐emulsions, lipid, and polymeric nanoparticles, are shown in **Figure** **17**. ^[^ ^231^ ^]^

The pathological mechanism of DR at the molecular level is crucial for choosing appropriate drugs for DR treatment. Recent studies have revealed that DR patients have elevated vascular endothelial growth factor (VEGF) levels in the intraocular space. VEGF is an endothelial‐cell‐specific angiogenic and vascular permeability factor whose production is increased by hypoxia. VEGF is the primary pathogenic factor in DR angiogenesis.^[^ ^232^ ^]^ DR treatment with intravitreal injection of anti‐VEGF monoclonal antibodies (mAb) or anti‐angiogenic factors (microRNA200 family) produces significant effects. Repeated injections are required because they have a relatively short duration of efficacy. However, the injections up to seven or eight times a year increase the risk of injection‐related complications, such as intraocular bleeding, endophthalmitis, pain, and discomfort, resulting in poor patient compliance. NPs, as drug carriers, can overcome the current limitations of intraocular anti‐VEGF mAb injection since they can improve therapeutic efficiency by extending the release period and increasing the bioavailability.^[^ ^230^ ^]^ Han et al.^[^ ^232^ ^]^ investigated the effect of microRNA200‐b (miR200‐b) on VEGF‐mediated angiogenesis using a mouse model with late‐onset diabetic retinopathy via a compacted DNA nanoparticle. The miR200‐b‐loaded DNA NPs significantly reduced the protein levels of VEGF receptor‐2 and markedly suppressed angiogenesis after three months of injection. They suggested that NP‐mediated miR200‐b delivery negatively regulates VEGF receptor‐2 expression in vivo, showing potential in the management of VEGF‐mediated neovascularization in DR.

Some synthetic anti‐VEGF agents, including Bevacizumab^[^ ^233^ ^]^ and Apatinib,^[^ ^234^ ^]^ are also involved in polymeric nanosystems to improve DR treatment, similar to the mechanism of anti‐VEGF mAb and miR200‐b. These studies reveal that anti‐VEGF drug‐loaded nanosystems can significantly enhance inhibition of VEGF expression and angiogenesis and reduce retinal vascular leakage, compared with the drug injection controls. Moreover, drug‐loaded nanosystems have a longer duration of action, demonstrating the great potential for preventing and treating diabetes‐induced retinal vascular disorders.

Besides anti‐VEGF agents, therapeutic drugs with anti‐inflammatory or anti‐angiogenic activities are widely used to alleviate DR since the inflammatory and neovascular mediators are involved in DR pathogenesis. Mahaling et al.^[^ ^235^ ^]^ reported a core‐shell nanoparticle (163 ± 4 nm, ‐21 ±5 mV), consisting of a hydrophobic polycaprolactone core and a hydrophilic Pluronic F68 shell, loaded with a corticosteroid drug triamcinolone acetonide (TA) for noninvasive treatment of DR in rats. When administered as eye drops, the core‐shell nanoparticles could transport TA to the retina in therapeutic concentration (approximately 25 µL, twice a day). Therefore, NPs can reduce inflammation and improve structural and functional activity of retina in DR rats due to the anti‐inflammatory, antiangiogenic, antiapoptotic, and neuroprotective properties of TA. Some other corticosteroids, such as dexamethasone and fluocinolone acetonide, also induce anti‐inflammatory effects during the treatment of acute and chronic posterior segment eye diseases. For instance, Da Silva et al.^[^ ^236^ ^]^ reported a biodegradable dexamethasone‐loaded polyurethane/clay NP with reduced potential irritancy and toxicity since they have similar mechanical properties with ocular soft tissues, thus providing temporary support for transplanted retinal pigment epithelial cells. Therefore, drug‐loaded nanosystems can enhance DR treatment.

#### 4.1.2 Nanoparticles with Antioxidant Property

**Figure 18:**
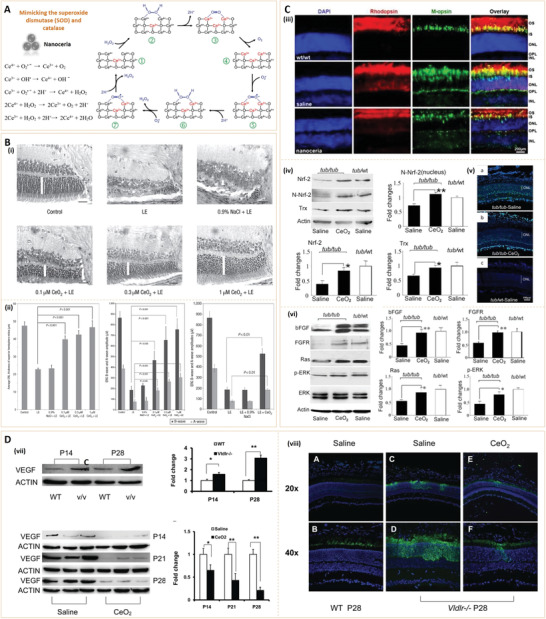
A) Proposed mechanisms of nanoceria‐based SOD mimic. Reproduced with permission.^[^ ^238^ ^]^ Copyright 2011, The Royal Society of Chemistry. B) Inhibition of ROIs via nanoceria particles (i) Intravitreal injection of nanoceria particles protects rat retina photoreceptor cells from light‐induced degeneration (ii). (i) Pre‐treatment of cultured retinal neurons with nanoceria particles (1 × 10^‐9^, 3 × 10^‐9^, 5 × 10^‐9^, 10 × 10^‐9^, and 20 × 10^‐9^ m) inhibits the intracellular accumulation of ROIs after exposure to 1 × 10^‐3^ m of H_2_O_2_ for 30 min. Incubation with nanoceria particles for a) 0.5 , b) 12, c) 24, and d) 96 h. (ii) Representative images of photomicrographs of H&E‐stained sections adjacent to the optic nerve. The white bars indicate the thickness of the layer of nuclei of rods and cones. a) No light exposure (LE) and no injection. Rats were exposed to 2700 lux white light for 6 h. b) No injection, c) injections of saline, d) 0.1 × 10^‐3^ m of CeO_2_, e) 0.3 × 10^‐3^ m of CeO_2_ and f) 1.0 × 10^‐3^ m of CeO_2_ 3 d before LE. The scale bars in the lower right corner represent 25 mm. Reproduced with permission.^[^ ^239^ ^]^ Copyright 2006, Nature Publishing Group. C‐iii) Photoreceptor‐specific proteins are mislocalized in tubby retinas and partially prevented by nanoceria. Immunocytochemistry at P28 reveals that rhodopsin (red) and M‐opsin (green) are localized in the OS of wt/wt retina. These proteins are mislocalized in the OS, ONL, OPL, and INL, representing the outer segment, inner segment outer nuclear layer, outer plexiform layer, and inner nuclear layer, respectively. Reproduced with permission.^[^ ^240^ ^]^ Copyright 2012, Elsevier Ltd. (iv) Nanoceria increases the expression of antioxidant‐associated proteins in the tubby retina. A) Western blot data and densitometric analysis showing increased B) Nrf‐2, C) N‐Nrf‐2 and D) Trx expression in the tubby retina to levels equivalent to the tub/wt controls after nanoceria treatment. The representatives of A) four Western blots and B–D) the average (mean ± SEM) of four retinas for quantitation. (v) ROS elevated in the tubby retina and decreased after nanoceria treatment. Intracellular ROS levels were detected using 2’, 7’‐dichlorodihydro‐fluorescein diacetate (DCFH‐DA) for hydrogen peroxide (H_2_O_2_) on fresh cryosections at P18. (vi) Nanoceria increases the expression of bFGF, FGFR, Ras, p‐ERK in the tubby retina. A) Western blots showing the expression levels of survival signaling pathway components. Densitometric analysis showing the elevation of B) bFGF, C) FGFR, D) Ras, and E) p‐ERK compared with the tub/wt levels when standardized to actin. The unchanged level of ERK protein after tubby mutation and nanoceria treatment (A). Reproduced with permission.^[^ ^241^ ^]^ Copyright 2011, Elsevier Ltd. D‐vii) Nanoceria prevents the ectopic increase of VEGF during postnatal development of the Vldlr‐/‐ retina. VEGF indicates vascular endothelial growth factor. (viii) Nanoceria inhibits the ectopic expression of VEGF in the ONL of the Vldlr‐/‐ retina. ONL indicates the outer nuclear layer. Reproduced with permission.^[^ ^242^ ^]^ Copyright 2011, The Authors. Published by PLOS.

The above studies indicate that nanomaterial‐mediated pharmacological therapies are effective for DR treatment. Furthermore, new therapeutic methods derived from some unique functions of nanomaterials are constantly emerging due to the development of nanotechnology and the understanding of DR pathological mechanisms. The intracellular concentration of reactive oxygen intermediates (ROIs) increases during the pathological process of DR. Photoreceptor cells have the highest rate of oxygen metabolism in the body and are continuously exposed to elevated levels of toxic ROIs. The deleterious effects of oxidative stress on sensitive cells of the retina could be the key cause of retinal diseases.^[^ ^237^ ^]^ Therefore, nanomaterials with antioxidant property have attracted increasing attention in recent years for both the treatment of diabetes and control of diabetic complications DR. CeO_2_ NP (nanoceria) (antidiabetic agent) has potential antioxidant ability due to the presence of mixed‐valence states of Ce^3+^ and Ce^4+^.^[^ ^89^ ^]^ The reactions involving redox cycles between the Ce^3+^ and Ce^4+^ oxidation states enhance the catalytic reaction between nanoceria and superoxide and hydrogen peroxide, mimicking the behavior of two key antioxidant enzymes, superoxide dismutase and catalase, potentially preventing all noxious intracellular ROS via a self‐regenerating mechanism (**Figure** **18**A).^[^ ^238^ ^]^ McGinnis et al. demonstrated for the first time that CeO_2_ NPs can prevent the increase in the intracellular concentrations of ROIs in primary cell cultures of rat retina in vitro and vision loss in an albino rat light damage model in vivo (Figure 18B).^[^ ^239^ ^]^ They concluded that the antioxidant effect of CeO_2_ particles may be effective in inhibiting the progression of ROI‐induced cell death. ROI‐induced cell death is involved in macular degeneration, retinitis pigmentosa, and other blinding diseases, including DR. They also indicated that CeO_2_ NPs can treat ROI‐induced death of other cell types in diabetes, Alzheimer's disease, atherosclerosis, stroke, etc. They also used the CeO_2_ NPs in the homozygous tubby mutant mouse to assess the short‐term (two weeks) and long‐term (more than one month) ability of nanoceria to slow the progression of retinal degeneration.^[^ ^240^, ^241^ ^]^ They found that CeO_2_ NPs protect the retina by decreasing ROS, up‐regulating the expression of neuroprotection‐associated genes, antioxidant associated genes and photoreceptor‐specific genes, down‐regulating apoptosis signaling pathways or upregulating survival signaling pathways, and decreasing the mislocalization of rod and cone opsins after a long‐term intravitreal injection (Figure 18C). Furthermore, McGinnis et al.^[^ ^242^ ^]^ found that ROS elimination caused by CeO_2_ NPs increase VEGF in the photoreceptor layer and the formation of the intraretinal and subretinal neovascular lesion based on a specific age‐related macular degeneration model (Figure 18D). Therefore, CeO_2_ NPs can prevent the downstream effects of oxidative stress in vivo, indicating that they can be used for treatment the of neurodegenerative diseases, such as macular degeneration and DR.

Similarly, some other NPs with intrinsic properties capable of addressing the pathological microenvironments of DR may also show potential for DR treatment. For instance, inorganic NPs composed of gold, silver, zinc oxide, and titanium dioxide exhibit outstanding anti‐angiogenic effects. The anti‐angiogenic effects of NPs may also have beneficial effects in choroidal and retinal neovascularization, involved in the development of vision‐threatening disorders, including diabetic retinopathy, retinopathy of prematurity after intravitreous injection, etc.^[^ ^243^ ^]^ In addition, most studies have shown that functional alterations and degeneration of retinal neurons precede and possibly activate the typical microangiopathic processes and disrupt the blood‐retinal barrier observed in DR. As a result, DR has enough pathophysiological traits to be considered a neurodegenerative eye disease. Therefore, a neuroprotective strategy may be used for the design of novel DR treatments.^[^ ^244^ ^]^

In conclusion, several researches on functional nanomaterials with antioxidative, anti‐inflammatory, anti‐angiogenic, or neuroprotective properties may be developed in the near future for the treatment of DR and other retinopathies. However, the safety, efficacy, and toxicology of these functional nanomaterials for ocular drug delivery are unknown. Therefore, further studies are needed to assess the above to accelerate the potential application of these functional nanomaterials in the treatment of DR and other retinopathies.

### 4.2 Nanomaterials in Diabetic Nephropathy

Diabetic nephropathy (DNep) is characterized by increased urinary albumin excretion (UAE) in the absence of other renal diseases. It is the leading cause of end‐stage renal disease, affecting about 90% of patients with diabetes.^[^ ^245^ ^]^ The pathological mechanisms of DNep include oxidative stress, derangement of glucose metabolism, and the activation of cell factors that cause cellular death.^[^ ^246^ ^]^ Therapeutic strategies for treating DNep usually involve controlling blood glucose levels and blood pressure. Drug therapeutics that inhibit the progression of renal dysfunction have been clinically nonsignificant,^[^ ^247^ ^]^ probably due to the inefficient administration, reduced distribution in the kidney, and adverse effects.^[^ ^248^, ^249^ ^]^

**Figure 19:**
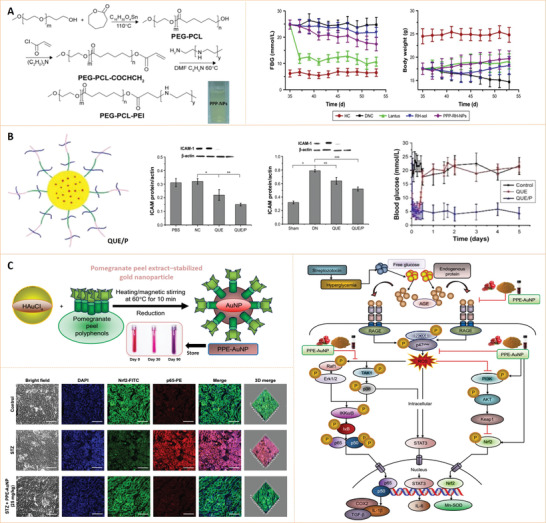
A) Kidney‐targeted drug delivery via rhein‐loaded polyethyleneglycol‐co‐polycaprolactone‐co‐polyethylenimine NPs (PPP NPs) for diabetic nephropathy therapy. Changes in FBG and body weight in different groups during treatment. FBG: fasting blood glucose; DN: diabetic nephropathy; DNC: DN control; HC: healthy control; rh‐sol: rh solution. Reproduced with permission.^[^ ^252^ ^]^ Copyright 2018, Dove Press Ltd. B) Therapeutic assessment of quercetin/PEG‐b‐(PELG‐g‐PZLL) (QUE/P) NP in diabetic nephropathy rats, including QUE/P downregulated ICAM‐1 expression in vivo as well as the activity of SOD and the levels of blood glucose, BUN, and Scr (iv). SOD: superoxide dismutase; BUN: blood urea nitrogen; Scr: serum creatinine. Reproduced with permission.^[^ ^246^ ^]^ Copyright 2017, Dove Press Ltd. C) Schematic representation of Au NP synthesis using PPE, the expression of Nrf2 and NF‐*κ*B (p65) in STZ‐induced diabetes mice when treated with PPE‐Au NP (25 mg kg^−1^) and the probable mechanism of action of PPE‐Au NP in ameliorating STZ‐induced diabetic nephropathy. PPE: pomegranate peel extract; PE: phycoerythrin; FITC: fluorescein. Reproduced with permission.^[^ ^254^ ^]^ Copyright 2019, Dove Press Ltd. D) Preparation of Pd‐Au HCDs/Ru(bpy)_2_(mcbpy)^2+^‐PEI‐ABEI@Ab/BSA bioconjugate and construction of ECL immunosensor, as well as the possible signal enhancement mechanism. Pd‐Au HCDs: convex hexoctahedral Pd@Au core‐shell nanocrystals; Ru(bpy)_2_(mcbpy)^2+^, Bis(2,2′‐bipyridyl)(4′‐Methyl‐[2,2] bipyridinyl‐4‐carboxylicacid) ruthenium (‖) dichloride; PEI: polyethyleneimine; ABEI: N‐(aminobutyl)‐N‐(ethylisoluminol); ECL: electrochemiluminescence. Reproduced with permission.^[^ ^74^ ^]^ Copyright 2017, Elsevier B.V.

Many natural products, such as rhein, have been used to treat DNep. Rhein is an anthraquinone derivative extracted from herbal medicines with various pharmacological effects, such as decreasing fasting blood glucose levels, creatinine, blood urea nitrogen, urine protein, oxidative stress, TGF‐*β*1 expression, etc.^[^ ^250^, ^251^ ^]^ However, the therapeutic effect of rhein on DNep is limited due to its poor water solubility, low bioavailability, and weak kidney targeting. Chen et al.^[^ ^252^ ^]^ synthesized a polyethyleneglycol‐*co*‐polycaprolactone‐*co*‐polyethylenimine (PEG‐PCL‐PEI) triblock amphiphilic polymer for the preparation of rhein‐loaded nanoparticles (PPP‐RH‐NPs) to enhance rhein delivery into the kidney and improve its therapeutic effect. They optimized the size of PPP‐RH‐NPs to 75 ± 25 nm for efficient kidney‐targeted drug delivery. The surface amine groups of polyethylenimine (positive charge) facilitate effective cellular uptake and quick endosomal escape. PPP‐RH‐NPs (200 µL/20 g) can improve the therapeutic effects of rhein on DNep by ameliorating several pathological indicators in a streptozocin‐induced DNep model (**Figure** **19**A). For instance, the levels of fasting blood glucose, body weight, the expression of TGF‐*β*1 in the kidney are significantly reduced in the PPP‐RH‐NPs group compared with the control groups. Besides rhein, crocetin (a phytoconstituent from Saffron, *Crocus sativus L*.) can also be used in nanosystems for DNep treatment.^[^ ^253^ ^]^ TGF‐*β*1 is an essential regulator of extracellular matrix (ECM) since it induces fibronectin and type IV collagen expressions in mesangial cells. The overexpression of TGF‐*β*1 can induce renal dysfunction, including albuminuria. Crocetin can down‐regulate the production and expression of fibrotic factors and fibronectin, which play significant roles in nephropathy pathogenesis. Therefore, nano‐formulation of crocetin can attenuate DNep symptoms.

Quercetin (QUE), a plant flavonoid, has sparked interest due to its antioxidant properties and potential as an effective drug for DNep. Delivery of QUE by NPs can improve its efficacy while decreasing side effects. Tong et al.^[^ ^246^ ^]^ demonstrated that a QUE‐loaded nanoparticle (QUE/P) had better therapeutic effects than free QUE in the treatment of DNep (hypodermic injection; Figure 19B). Although both free QUE (10 mg kg^‐1^) and QUE‐loaded NPs (10 mg kg^‐1^) downregulated the intercellular adhesion molecular‐1 (ICAM‐1) expression in the endothelium, QUE‐loaded NPs suppressed ICAM‐1 more significantly. Moreover, blood glucose, blood urine nitrogen (BUN), and serum creatinine (Scr) tests revealed that NP‐mediated QUE delivery (QUE/P) was the most effective. Since superoxide dismutase (SOD) activity was highest in the QUE/P group, we deduced that the antioxidant of QUE contributed considerably to the high therapeutic effect by relieving oxidative stress in DNep.

Similar to the treatment for diabetes, inorganic NPs or nanocomposites with antioxidant properties to neutralize ROS or quench oxidative stress were also used to treat DNep. A recent study found that antioxidative Se NPs reduced the severity of DNep.^[^ ^255^ ^]^ The authors discovered the ability of Se NPs to reduce oxidative stress accompanied by the activation of cytoprotective heat stress protein (HSP‐70) and the longevity protein SIRT‐1. When cells are under stress, HSP‐70, an inducible protein, prevents apoptotic cell death by modulating various apoptotic proteins, whereas SIRT‐1 enables the cell to survive under stressful conditions. With the highlighted positive functions, Se NPs effectively impeded the progression of DNep, implying their therapeutic potential.

It is emphasized that oxidative stress is the fundamental cause of DNep, and that it is elevated in diabetic kidneys before clinical signs of nephropathy appear.^[^ ^256^ ^]^ Furthermore, mounting evidence indicates that ROS‐mediated activation of the advanced glycated end products (AGE) receptor activates a molecular switch linked to the activation of NF‐*κ*B, resulting in renal inflammation. In contrast, Nrf2, a redox‐sensitive transcription factor, is involved in the initiation of cellular defense mechanisms and has been shown to play a protective role in DNep, as a new therapy option. Consequently, Manna et al.^[^ ^254^ ^]^ evaluated the meliorative role of pomegranate peel extract‐stabilized Au NP (PPE‐AuNP, 20.4 ± 0.347 nm) in streptozotocin‐induced DNep mice by assessing the NF‐*κ*B and Nrf2 signaling systems (Figure 19C). Immunofluorescence images revealed no significant differences in the expression levels of Nrf2 and NF‐*κ*B (p65) between streptozotocin‐induced diabetes mice and normal mice (control) after treatment with PPE‐AuNP (5, 15, and 25 mg kg^‐1^). The results indicated that the PPE‐Au NP reversed the oxidative state by inhibiting protein glycation, scavenging the ROS, and reducing the dephosphorylation of MAPK/NF‐*κ*B/STAT3‐mediated proinflammatory burden. The endogenous antioxidant response of PPE‐Au NPs was found to be beneficial in maintaining renal homeostasis in DNep mice (Figure 19C). Similarly, Alomari et al.^[^ ^257^ ^]^ discovered that Au NPs (50 nm) can ameliorate renal damage in DNep models by improving renal function and downregulating extracellular matrix protein accumulation, as well as inhibiting renal oxidative stress and reducing podocyte injury. Overall, these results demonstrated a potential therapeutic efficiency of Au NPs on DNep, albeit more research is needed to determine the exact mechanisms of action and physiological levels. Nevertheless, it can be concluded that antioxidants contribute greatly to DNep therapy and may open up novel and economical treatment options for diabetic progression and hyperglycemia‐induced complications.

In addition to treatment, nanomaterials have been found to be crucial in early diagnosis and therapy monitoring in DNep. For example, Liu et al.^[^ ^258^ ^]^ recently developed a sensitive electrochemiluminescence (ECL) immunosensor based on Pd@Au core‐shell nanoparticles with ABEI (*N*‐(aminobutyl)‐*N*‐(ethylisoluminol)) (55 ± 10 nm) and catalyzer (Fc, ferrocene monocarboxylic acid) bifunctionalization for the detection of human collagen type IV (Col IV), a typical biomarker that is associated with DNep. The immunosensor detected Col IV at concentrations ranging from 1 pg mL^−1^ to 10 ng mL^−1^, with a relatively low detection limit of 0.3 pg mL^−1^. The same group further constructed another sensitive immunosensor based on a tris(bipyridine) ruthenium (II) derivative (Ru(bpy)_2_(mcbpy)^2+^‐PEI‐ABEI) (Figure 19D). In their work, polyethyleneimine (PEI), which acted as a classic coreactant with a large number of amine co‐reactive groups, was found to significantly boost the ECL intensity of Ru(bpy)_2_(mcbpy)^2+^, especially through the intramolecular coreaction. Moreover, the intramolecular ECL resonance energy transfer (ECL‐RET) from PEI‐ABEI increased the ECL intensity of Ru(bpy)_2_(mcbpy)^2+^ (Figure 19D). Consequently, the novel immunosensor increased the detection range for Col IV from 0.5 pg mL^−1^ to 7.2 ng mL^−1^ and lowered the detection limit to 0.17 pg mL^−1^. The studies indicated that functional nanomaterials are comprehensively promising for not only treatment but also early diagnosis and monitoring of DNep progression.

### 4.3 Nanomaterials in Diabetic Neuropathy

**Figure 20:**
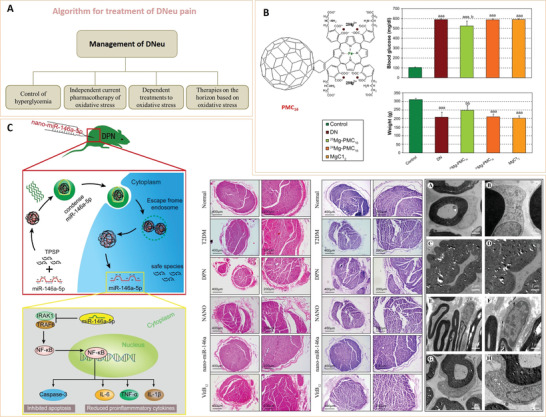
A) Algorithm for treatment of DNeu pain. Reproduced with permission.^[^ ^261^ ^]^ Copyright 2013, Hindawi Publishing Corporation. B) The PMC_16_ (Porphylleren‐MC16) NP structure and protective effects of various forms of magnesium on blood glucose and weight in DNeu rats after two months. Left‐hand side: Reproduced with permission.^[^ ^262^ ^]^ Copyright 2008, Elsevier Masson SAS. Right‐hand side: Reproduced with permission.^[^ ^263^ ^]^ Copyright 2010, Dove Press Ltd. Neuromorphology images of H&E staining, Luxol fast blue staining, and toluidine blue staining (iv) of the sciatic nerve from rats in each group; The ultrastructure of myelin in the sciatic nerve of rats from each group. A,B) Normal group; C,D) DPN group; E,F) NANO group; G,H) nano‐miR‐146a‐5p group; (vi) The expression levels of inflammatory response‐ and apoptosis‐related proteins in each group. NANO: empty NP, IL‐1*β*: interleukin 1 beta, IL‐6: interleukin 6, IL‐10: interleukin 10, COX‐2: cyclooxygenase‐2, and TNF‐*α*: tumor necrosis factor‐*α*. Reproduced with permission.^[^ ^268^ ^]^ Copyright 2019, Elsevier Inc.

Diabetic neuropathy (DNeu) is a group of neurological disorders associated with diabetes that affects over 50% of diabetic patients. High blood glucose and its toxic by‐products are the main causes of nerve dysfunction.^[^ ^259^, ^260^ ^]^ Similar to DNep, DNeu develops on a background of hyperglycemia and related metabolic imbalances, including mitochondrial dysfunction, decline of adenosine triphosphate (ATP), and oxidative stress‐induced neural death. There is currently no efficient clinical treatment for human DNeu, and practically all drugs (e.g., tricyclic antidepressants, anticonvulsant agents, and aldose reductase inhibitors) are used only to manage the disorder (**Figure** **20**A).^[^ ^261^ ^]^

Although the treatment of DNeu in clinics has stopped, research into experimental DNeu continues, particularly with the rapid development of functional nanomaterials. Fullerene‐C_60_‐based nanocationite particle with magnetic isotope of magnesium (^25^Mg‐PMC_16_) has low toxicity and high membranotropic antioxidant effect.^[^ ^262^ ^]^ The ^25^Mg‐PMC_16_ NPs selectively stimulated ATP overproduction in the oxygen‐depleted cells due to the release of ^25^Mg^2+^. Injecting rats with ^25^Mg‐PMC_16_ NPs protected their heart muscles against serious damage in a vast variety of hypoxia‐caused clinical conditions. Consequently, Hosseini et al.^[^ ^263^ ^]^ studied the protective effect of NPs in streptozotocin‐induced DNeu rats. They deduced that, by reducing oxidative stress and increasing ATP in dorsal root ganglion neurons, ^25^Mg‐PMC_16_ NPs would protect mitochondrial dysfunction in DNeu. As expected, although oxidative stress in DNeu damaged dorsal root ganglion neurons and eventually killed them, two consecutive days of ^25^Mg‐PMC_16_ NPs (0.5 lethal dose_50_) treatment protected rats from streptozotocin‐induced DNeu by reducing oxidative stress and improving ATP levels (Figure 20B).

Neuron‐targeted nanosystems capable of delivering therapeutic genes, in addition to antioxidant nanomaterials, are viable techniques for treating diabetic neuropathies.^[^ ^264^ ^]^ Nanomaterials offer the possibility to treat a variety of neuropathies by mediating gene delivery within nervous tissue.^[^ ^265^ ^]^ Lopes et al.^[^ ^266^ ^]^ highlighted the potential of polymeric NPs as non‐viral gene carriers to deliver therapeutic genes into peripheral neurons, allowing their application as an effective therapeutic intervention for neuropathies. Furthermore, Feng et al.^[^ ^267^ ^]^ reported that specific miRNAs (e.g., microRNA‐146a‐5p and miR‐146a‐5p) could be used to treat diabetic peripheral neuropathy (DPN) by suppressing hyperglycemia‐induced proinflammatory genes. Based on this background, Luo et al. ^[^ ^268^ ^]^ designed an imine backbone‐based polymeric nano‐carrier (TPSP) for miR‐146a‐5p delivery and investigated its therapeutic effects on Type‐II diabetes and DPN. In contrast to previous studies that used chemical drugs or miR‐146a‐5p mimics,^[^ ^269^, ^270^ ^]^ this work adopted low‐toxicity and nonimmunogenic cationic nanocarriers to create a nano‐miR‐146a‐5p that prevented siRNA leakage and nonspecific adsorption before reaching the target cells. The results showed that there was no obvious differences between the nano‐miR‐146a‐5p (1nmol per rat) and the vitamin B12 (VitB12, 550 µg kg^‐1^) groups in terms of histopathological and ultrastructure changes in myelin (Figure 20C). Since VitB_12_ is an indispensable vitamin for the nervous system and is involved in the synthesis of a lipoprotein in nerve tissue, the result indicated that nano‐miR‐146a‐5p had a comparable effect on peripheral nerve protection of the DPN rat model. Because typical virus vectors commonly suffer from their immunogenicity, the beneficial effects of nano‐miR‐146a‐5p on decreasing inflammatory response‐ and apoptosis‐related proteins levels are extremely valuable. Further studies revealed that there were no discernible differences in the ability of nano‐miR‐146a‐5p and ViB_12_ to regulate inflammation‐related proteins, implying that nanosystems for miR‐146a‐5p delivery could be a potential strategy for inhibiting inflammation and ameliorating apoptosis in DNeu.

Nanomaterial therapy in DNeu is an area in its infancy. Apart from the reported ^25^Mg‐PMC_16_ NP and nano‐miR‐146a‐5p, other antioxidants or anti‐inflammatory nano‐formats with enhanced functionalities and targets for DNeu treatment could be identified. Nevertheless, the available studies have only provided experimental evidence, and more research into the potential uses of nanomaterials as an improved therapeutic approach in DNeu treatment are highly anticipated.

### 4.4 Nanomaterials in Diabetic Cardiomyopathy

Diabetic cardiomyopathy (DC) is characterized by impaired cardiac diastolic and systolic functions and is associated with both Type‐I and II‐diabetes.^[^ ^271^ ^]^ Myocyte hypertrophy and myocardial fibrosis are the most frequently proposed mechanisms to explain cardiac changes in DC.^[^ ^272^ ^]^ Similar to diabetes and other complications, oxidative stress plays a key role in the pathophysiology of cardiac hypertrophic remodeling and dysfunction.^[^ ^273^ ^]^ Therefore, the effects of several natural products on DC treatment through antioxidant mechanism has been extensively explored.^[^ ^274^ ^]^ Curcumin, a natural chemical, has been used in traditional Chinese medicine for thousands of years as a diabetes treatment and as a means to decrease the risk of cardiovascular diseases. Since NP technology improves poor bioavailability and side effects of curcumin, Abdel‐Mageid et al.^[^ ^271^ ^]^ used an aqueous dispersion method to prepare a nano‐curcumin with the average size of around 200 nm, which was used to investigate the therapeutic potential of nano‐curcumin on DC attenuation. Nano‐curcumin has been shown to reduce cardiac inflammation, myocardial fibrosis, and programmed myocardial cell death through the inhibition of advanced glycation end products (AGEPs) accumulation and oxidative stress in heart tissue of chronic streptozotocin‐induced diabetic rats.

**Figure 21:**
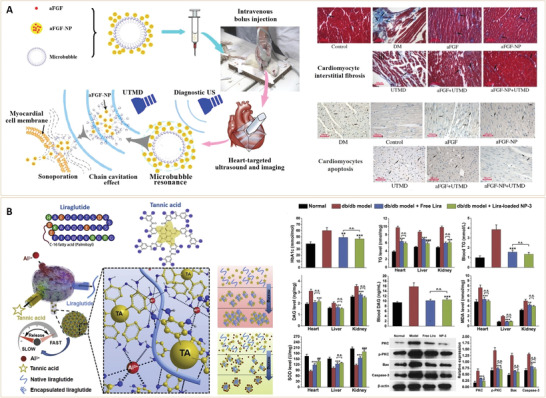
A) Schematic illustration of using UTMD technique in aFGF targeted delivery. aFGF‐NP prevented DM‐induced metabolism abnormalities and cardiomyocyte interstitial fibrosis: quantitative analysis of the blood glucose of rats (left), the body weight of rats (middle), and the heart‐to‐body ratio (HW/BW) of rats (right); representative pictures of myocardial tissue sections stained with Masson trichrome (400 ×). Arrow indicates myocardial fibrosis stained in blue; quantitative analysis of cardiac collagen volume fraction. Reproduced with permission.^[^ ^277^ ^]^ Copyright 2016, Elsevier B.V. B) Schematic illustration of ternary nanoparticle assembly. Lira: Liraglutide; TA: tannic acid. Long‐term cardiovascular protective effect of multiadministrated Lira‐loaded NP‐3. HbA1c: glycosylated hemoglobin, TG: triglyceride, DAG: diacylglycerol, MDA: malondialdehyde, and SOD: superoxidase. Reproduced with permission.^[^ ^275^ ^]^ Copyright 2019, Elsevier B.V.

Synthetic drugs, in addition to natural products, in nanosystems have been developed to improve cardiovascular function in diabetic patients. Liraglutide (Lira) is a GLP‐1 (glucagon‐like peptide‐1) receptor agonist that was recently approved for the treatment of Type‐II diabetes. It has a superior hypoglycemic effect while also improving cardiovascular functions in patients. However, the drug's short half‐life (≈13 h) makes clinical application difficult, necessitating urgent development of a long‐acting release system for Lira. He et al.^[^ ^275^ ^]^ described a ternary liraglutide/tannic acid (TA)/Al^3+^ nanosystem based on hydrogen bond formation between liraglutide and TA for DC prevention (**Figure** **21**A). In acidic conditions (pH < 6.0), TA can form complexes with Lira through hydrogen bonding. Moreover, Al^3+^, a trivalent metal ion, was used to quench the excessive phenol groups through coordination complexation, and halt the excessive growth of TA‐Lira complexes. Under physiological conditions (pH 7.4), partial ionization of phenol groups can weaken the hydrogen bonding, resulting in an electrolytic environment that could trigger decomplexation and release of Lira. The nanoparticle system could considerably improve therapeutic effects due to its long‐acting glycemic control and improved cardiovascular functions, in addition to reduced tissue damage in numerous organs (Figure 21A). Similarly, acidic fibroblast growth factor (aFGF) is a 15.8 kDa peptide with potential cardiomyopathy therapeutic benefits in diabetic patients.^[^ ^276^ ^]^ Suppression of oxidative stress and myocardial damage in hyperglycemia is the biological mechanism through which aFGF protects cardiovascular functioning. However, optimizing the mode of aFGF delivery to reduce its impact on systemic tissues (e.g., liver, spleen, lung, and kidney), while maintaining bioactivity in myocardial tissues is critical. Although nanocarriers for aFGF have been proven to be less risky and to improve aFGF stability both in vitro and in vivo, efficient delivery of aFGF to heart tissues with less side‐effects is still challenging. To increase the selectivity of nanocarriers towards cardiac tissues, Zhao et al.^[^ ^277^ ^]^ developed aFGF‐loaded heparin‐based NPs (aFGF‐NPs, 128 ± 1.65 nm) for enhanced DC prevention, assisted by an ultrasound‐targeted microbubble destruction (UTMD) technique (Figure 21B). Ultrasound could modify the permeability of cell plasma membrane, which is often termed as sonoporation. A localized sonoporation can be used to realize selectively enhanced endocytosis. In their work, UTMD‐mediated targeted aFGF delivery was achieved by administering aFGF‐NPs (15 µg kg^‐1^) with localized sonoporation. In vivo results strongly suggested that aFGF‐NPs used in conjunction with UTMD were the most effective in reducing apoptosis and fibrosis of cardiomyocytes, and increasing the density of microvasulature, showing the translational potential in delivery of aFGF for the prevention of DC.

Certain nanomaterials per se can reduce the possibility of cardiovascular disorders, when similar routines are used for other complications, due to their great efficiency in decreasing oxidative damages. However, the dosage of antioxidant NPs for cardiovascular system should be meticulously examined, because of not only the effective dose for cardioprotection but also the potential negative effects on the life‐related organ. For example, a moderate dose (3 mg kg^−1^ body weight, BW) of antioxidant ZnO NPs was found to reverse cardiac damage in rats with streptozotocin‐induced cardiomyopathy. After oral administration of moderate ZnO NPs, serum cholesterol, high density lipoprotein‐cholesterol, lipoprotein, tumor necrosis factor‐*α*, cardiac malondialdehyde, B‐type natriuretic peptide levels, as well as the atherogenic index and caspase‐3 activity were significantly reduced. Furthermore, apoptosis indices and histopathological features were also improved with a moderate dose. However, a higher dose (10 mg kg^−1^BW) was found to be toxic and resulted in aggravation of the injuries.^[^ ^278^ ^]^ The cardiac effect of Ag nanoparticles was also evaluated by Ramirez‐Lee et al.^[^ ^279^ ^]^ who found that Ag NPs can enter the cardiovascular system after dermal, inhalation, or oral exposure, and spread to the major organs, including the heart. High concentrations of Ag NPs were found to increase measured perfusion pressure, left ventricular pressure, and oxidative stress. Low concentrations of Ag NPs had no significant effect. In light of the stated results, the correlation between the NP dosage and cardioprotective effect needs to be further explored, despite the great potential of antioxidant nanoparticles in DC prevention.

### 4.5 Nanomaterials in Diabetic Wound Repair

Impaired wound healing is a typical complication and a major health concern that occur in diabetic patients. Diabetic chronic ulcers have become a great challenge for both patients and health care systems.^[^ ^280^, ^281^ ^]^ Several factors contribute to diabetic chronic ulcers disorder, including neuropathy, infection, and vascular injuries.^[^ ^282^ ^]^ First, chronic hyperglycemia leads to the development of advanced glycation end products (AGEs), which are sugar‐derived substances that obstruct wound healing by suspending the inflammatory and proliferative phases. Advanced glycation end products stimulate the production of free radicals which cause a discrepancy between the levels of free radicals and antioxidants.^[^ ^283^ ^]^ Impaired leukocyte functions of diabetics commonly result in limited neutrophils and macrophage migration to wound sites. Overall, the aforementioned pathological processes jointly contribute to delayed wound healing. To accelerate diabetic wound healing process, a variety of materials with essential biocompatibility and improved properties have been developed.^[^ ^284^, ^285^ ^]^ Functional nanomaterials outperform conventional wound protective dressings in terms of their ability to carry, protect, and sustain appropriate drug release to the wound surface for prolonged treatment.^[^ ^286^ ^]^ In recent years, direct use of nanoparticles or encapsulating of nanomaterials into wound protective dressings have been well developed for diabetic wound repair.

#### 4.5.1 Nanoparticles for the Treatment of Infected Diabetic Wound

Bacterial infections are among the most crucial factors that adversely affect the healing of diabetic wounds. This is because diabetic chronic ulcers, especially those on the skin, are more likely to contact pathogenic bacteria. Therefore, preventing bacterial infections when treating a diabetic wound is imperative. Certain metallic/metal oxide (e.g., silver, copper, and cerium oxide) and nonmetallic nanoparticles (e.g., graphene oxides) have been found to have antibacterial/antibiotic properties and a low risk of generating drug‐resistant bacteria. Therefore, such nanomaterials might provide an unrivaled approach for accelerating the healing of delayed acute and chronic wounds.^[^ ^287^ ^]^

Silver (Ag) is well known for its broad‐spectrum antibacterial property, which is mediated by blocking respiratory enzyme pathways and altering microbial DNA and the cell walls.^[^ ^288^ ^]^ Silver NPs have been reported as intrinsic therapeutic agents for promoting the healing of chronic wounds.^[^ ^289^, ^290^ ^]^ Silver NPs are capable of removing microbes that might otherwise interrupt and delay the normal phases of healing, and have potential to promote diabetic wound healing.^[^ ^287^ ^]^ In addition to antimicrobial properties, Krishnan et al.^[^ ^289^ ^]^ demonstrated that biogenic Ag NPs reduced the mRNA and protein expression of matrix metalloproteinases 2 and 9 (MMP‐2 and MMP‐9) in wounded granulation tissues, leading to faster wound healing in diabetic mice. Furthermore, this type of Ag NPs has been proven to be effective in the treatment of Gram‐negative bacilli infection in diabetic wounds.^[^ ^291^ ^]^ In contrast to the unique antibacterial property of Ag NPs, CeO_2_ NPs were first recognized as therapeutic agents for diabetic treatment due to their antioxidant property and low toxicity.^[^ ^238^, ^292^ ^]^ CeO_2_ NPs are preferentially exploited in the treatment of diabetic foot ulcers due to their oxygen vacancy and free radical scavenging properties.^[^ ^293^ ^]^ In addition, CeO_2_ NPs possess excellent antibacterial properties. Kobyliak et al.^[^ ^294^ ^]^ described the first case report of successful topical treatment of neuropathic diabetic foot ulcers using novel gel containing CeO_2_ NPs. The CeO_2_ NPs penetrated into the wound tissues, reduced the oxidative damages, killed pathogens, and protected the regenerative tissues. As a result of the bacteriostatic activity and anti‐inflammatory properties, CeO_2_ NPs prevented secondary infections of the wound, suggesting that they have good therapeutic potential for the topical treatment of diabetic chronic ulcers.

In addition to metallic/metal oxide, nonmetallic NPs were also proposed as curing agents for wound healing. Carbon‐based nanomaterials, including graphene oxide (GO), carbon nanotube (CN), and fullerenes, have shown tremendous potential in nanomedicine like bioimaging, tissue regeneration, and controllable medicinal products.^[^ ^295^ ^]^ Recent studies have established that GO has excellent antibacterial and angiogenic properties.^[^ ^296^ ^]^ Another study demonstrated that the different adhesive and bioactive properties of GO can prevent bacterial adhesion.^[^ ^297^ ^]^ Taken together, these studies suggested that GO is a potential agent for effective wound healing. Similarly, CN and fullerenes exhibit potential for wound healing due to their antibacterial, antioxidant, and anti‐inflammatory properties.^[^ ^298^ ^]^ Further explorations on these nanomaterials would reveal more detailed mechanisms, as well as more treatment options for diabetic chronic ulcers.

#### 4.5.2 Nanoparticles for Wound Dressing Design

When NPs are incorporated with biopolymeric materials, they act as a potential wound healing material for diabetic ulcer wounds.^[^ ^283^ ^]^ Antibacterial properties are crucial in wound healing. Thus, Ag NPs are widely used for wound healing purposes.^[^ ^299^, ^300^, ^301^ ^]^ Masood et al.^[^ ^290^ ^]^ designed an Ag NP‐loaded CS‐PEG hydrogel for the treatment of chronic diabetic wounds, which considerably accelerated wound healing in diabetes mellitus due to the special synergy between Ag NPs and CS. Similarly, Anisha et al.^[^ ^302^ ^]^ developed an antimicrobial sponge composed of CS, HA, and Ag NPs as a wound dressing for diabetic foot ulcers (DFU). Despite infection with drug‐resistant bacteria, the hydrogel‐based antibacterial wound dressing provides a moist environment, protects the wounded tissue surface from dryness, and promotes wound healing due to its good hydrophilic property and unique viscoelastic nature.^[^ ^303^ ^]^ Undoubtedly, the incorporation of antibacterial NPs into biopolymer materials may result in promising wound dressings for accelerating diabetic ulcer repair, especially in infected wounds.

**Figure 22:**
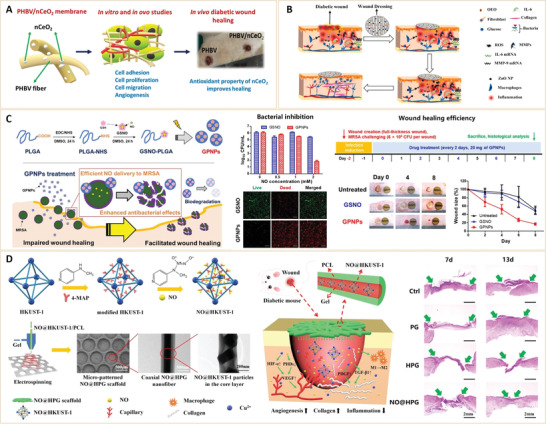
A) CeO_2_ NP incorporated electrospun poly(3‐hydroxybutyrate‐co‐3‐ hydroxyvalerate) membranes for diabetic wound healing. PHBV: poly(3‐hydroxybutyrate‐co‐3‐hydroxyvalerate). Reproduced with permission.^[^ ^305^ ^]^ Copyright 2020, American Chemical Society. B) Schematic illustration of the mechanism of action of the proposed wound dressing on the diabetic skin wound model. Reproduced with permission.^[^ ^304^ ^]^ Copyright 2021, The Authors. Published by Elsevier. C) Nitric oxide‐releasing GSNO conjugated PLGA NPs for the treatment of MRSA‐infected cutaneous wounds. NO release profile of GPNPs in simulated wound fluid (SWF) at 37 °C; Antibacterial effects of GPNPs against MRSA compared to that of GSN. Green fluorescence (SYTO 9) and red fluorescence (propidium iodide) indicate live and dead bacteria, respectively; In vivo wound‐healing promotion effects of GPNPs in a MRSA‐challenged full‐thickness wound mouse model. GSNO: S‐nitrosoglutathione, PLGA: poly(lactic‐co‐glycolic acid), and MRSA: *Staphylococcus aureus*. Reproduced with permission.^[^ ^309^ ^]^ Copyright 2020, MDPI. D) Schematic Illustration of the preparation of NO@HKUST‐1 and NO@HKUST‐1/PCL/Gel (NO@HPG) scaffold; Composite scaffold promotes the healing of diabetic wound. HKUST‐1, a kind of metal‐organic frameworks with copper ions as the metal center and trimesic acid (H_3_btc) as the organic ligand; PCL: hydrophobic polycaprolactone and Gel: gelatin. Reproduced with permission.^[^ ^306^ ^]^ Copyright 2020, American Chemical Society.

Incorporating antioxidant NPs into wound dressings may further improve performance by accelerating the wound healing process.^[^ ^304^ ^]^ Augustine et al.^[^ ^305^ ^]^ prepared an electrospun poly(3‐hydroxybutyrate‐co‐3‐hydroxyvalerate) (PHBV) membranes with antioxidant CeO_2_ NPs (nCeO_2_) for diabetic wound repair (**Figure** **22**A). As a result of the antioxidant and angiogenic activities of nCeO_2_ NPs, the nCeO_2_‐loaded electrospun membranes (PHBV/nCeO_2_) exhibited excellent cytocompatibility for cell adhesion, cell proliferation, cell migration, and angiogenesis in vivo. When used as a wound dressing in diabetic rats, the antioxidant membranes PHBV/nCeO_2_ showed considerably enhanced diabetic wound healing properties compared to the nCeO_2_‐free membrane (PHBV). In addition, Khan et al.^[^ ^304^ ^]^ developed a ZnO‐loaded core‐shell electrospun membrane for the treatment of diabetic wounds (Figure 22B). To enhance efficiency, Atta et al. employed oregano essential oil (OEO), an antioxidant and antibacterial natural plant extract. They anticipated that the synergetic anti‐inflammatory actions of co‐loaded ZnO and OEO will enhance the healing rates. As expected, the bi‐component electrospun membrane successfully healed the wound with rapid epithelialization, neo‐vascularization, and collagen deposition. An in‐depth study revealed that the membranes exhibited anti‐inflammatory activities by downregulating the inflammatory‐related gene expression, indicating that ZnO and OEO have a considerable influence on the elimination of oxidative stress. These studies reflected to the possibility that antioxidant NPs in wound dressing may act as important agents in the healing of diabetic wounds.

Since nanomaterials may also serve as carriers for therapeutic agents in wound dressing design, wound dressings may have more biological functions that match the healing process of diabetes‐related chronic ulcers. During the wound‐healing process, endogenous nitric oxide (NO) synthesis has been shown to inhibit inflammation, promote angiogenesis, and stimulate collagen deposition.^[^ ^306^ ^]^ However, decreased NO bioavailability in Type‐II diabetes contributes to impaired wound healing.^[^ ^307^ ^]^ Therefore, the delivery of NO to the wound sites of diabetic patients is anticipated. Indeed, NO exhibits perfect antibacterial property.^[^ ^308^ ^]^ Lee et al.^[^ ^309^ ^]^ fabricated a S‐nitrosoglutathione (GSNO)‐conjugated PLGA NPs (GPNPs) to prolong NO release. Degradation of the GSNO groups in GPNPs led to a continuous controlled NO release for up to 12 h. Consequently, nanoparticles (20 mg) showed excellent antibacterial efficacy against methicillin‐resistant *Staphylococcus aureus* (MRSA) and accelerated cutaneous wound closure in vivo (Figure 22C). The study revealed that NO‐releasing nanomaterials have a great potential for diabetic chronic wound repair. With this background, NO‐releasing wound dressings aroused concerns. In comparison with other nanomaterials, MOFs exhibit superiority in NO delivery due to their porosity, high specific surface area, and coordinatively unsaturated metal sites (CUS) capable of bonding with NO.^[^ ^310^ ^]^ In addition, the release of coordinative metal ions (e.g., cobalt, zinc, and copper) may also play an important role in wound healing. Consequently, Zhang et al.^[^ ^306^ ^]^ prepared a copper‐based MOF (HKUST‐1) as a NO‐loading vehicle and designed a fibrous NO‐releasing wound dressing using electrospinning of hydrophobic polycaprolactone (PCL) and gelatin (Gel) (Figure 22D). The exudate‐induced degradation of nanofibers at the wound site led to the release of NO and copper ions. Moreover, the release behavior of NO and copper ions can be further controlled by modifying the structure of the coaxial fiber during the electrospinning process. The results showed that the copper ions released by the degradable HKUST‐1 played a synergistic role with NO to promote endothelial cell growth and significantly improve the angiogenesis, collagen deposition, and anti‐inflammatory properties in the wound bed, resulting in faster diabetic wound healing (Figure 22D). The study implied that such kind of NO‐loaded MOF nanoparticles for wound dressing fabrication would provide a promising strategy for highly efficient diabetic wound repair.

Nanofibers can also act as a vehicle for drug delivery.^[^ ^311^ ^]^ Various drugs and biomolecules have been incorporated within nanofibers during fabrication or post‐modification methods to enhance their functional properties for biomedical applications.^[^ ^312^ ^]^ Recently, nanofibers have been studied for delivering therapeutic active components (i.e., growth factors,^[^ ^313^ ^]^ metformin,^[^ ^314^, ^315^ ^]^ curcumin,^[^ ^316^ ^]^ etc.) to wound sites. The functional fibrous scaffolds influenced diabetic wound healing by stimulating cell proliferation, migration, angiogenesis, collagen secretion, and inhibiting microbes.^[^ ^317^ ^]^ For example, Bhadauriya et al.^[^ ^318^ ^]^ created a carbon nanofiber (CNF) wound dressing that was immobilized with yeast extract (YE) and encapsulated Cu NPs (Cu‐CNF‐YE) to simultaneously control the glucose and bacterial infections in diabetic wounds. Owing to the antibacterial characteristics of the immobilized YE and Cu ions, the Cu‐CNF‐YE effectively inhibited *E. coli* and *S. aureus* strains. Subsequently, a 1 cm circular full thickness skin wound was created in diabetic Wistar rats to evaluate the efficiency of in vivo wound repair. The results showed that in the group treated with Cu‐CNF‐YE dressing, fibrous tissues with scars appeared, and the initiation of proper dermis and epidermis formation as well as neovascularization and a few hair follicles were observed at the periphery of defect. Complete wound closure occurred with a ≈0.9 mm fibrous scar tissues in the Cu‐CNF‐YE dressing group, demonstrating excellent wound repair. In addition, the Cu‐CNF‐YE showed a significant consumption of glucose. Taken altogether, this multifunctional fibrous wound dressing can be applied as an effective material to treat both diabetes and diabetic wounds.

**Figure 23:**
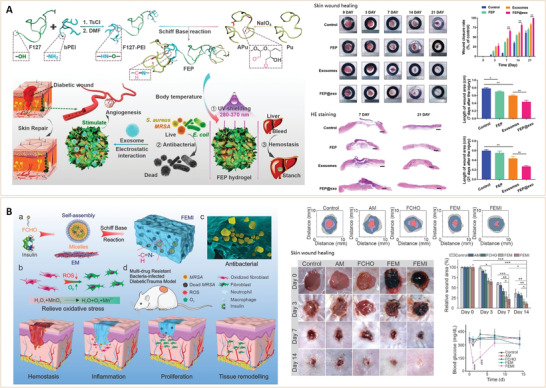
A) Schematic illustration showing the synthesis of F127‐PEI, APu, multifunctional FEP scaffold dressing, nanoscale exosome‐loaded FEP scaffold dressing, and the potential application in diabetic wound healing and skin reconstruction. Representative images of wound healing process in mice treated with control, FEP, exosomes, and FEP@exo; Wound closure rates at different time points of four group. Reproduced with permission.^[^ ^319^ ^]^ Copyright 2019, American Chemical Society. B) Schematic diagram illustrating FEMI hydrogel for MDR bacteria‐infected diabetic wound healing. a) The FEMI hydrogel was fabricated by reversible Schiff‐based reaction between EM and insulin loaded FCHO micelles. b) The FEMI hydrogel could protect fibroblasts from oxidative stress by decomposing the extensive ROS (H_2_O_2_) into O_2_; c) An efficient antibacterial performance was achieved synergistically through the positive charged EPL and the sharp “nano‐knife” like MnO_2_ nanosheets; d) FEM hydrogel contributed to the simulative wound healing in vivo by accelerating hemostasis, eradicating MDR infection, consuming large amounts of deleterious ROS, and ameliorating the perpetuate inflammatory microenvironment. Representative images of the wound healing process in mice treated with control, antibiotic ampicillin, FCHO, FEM, and FEMI hydrogels; (iv) In vivo wound closure rates of the five groups at different time points. Reproduced with permission.^[^ ^323^ ^]^ Copyright 2020, American Chemical Society.

In addition to fibrous scaffolds, the biomedical hydrogel has been investigated as a promising medium for delivering drug/cells diabetic wound healing therapies.^[^ ^63^, ^319^, ^320^, ^321^ ^]^ Hydrogels are water‐rich hydrophilic 3D polymer networks with configurable viscoelasticity and biocompatibility. Biomolecules and NPs can be incorporated in hydrogels, allowing for more advanced topical drug delivery with unique benefits such as improved tissue localization, minimized burst release, and controlled sequential drug release.^[^ ^322^ ^]^ Wang et al.^[^ ^319^ ^]^ fabricated an injectable adhesive thermosensitive multifunctional polysaccharide‐based dressing (FEP) with sustained pH‐responsive exosome (exo) release for promoting angiogenesis and diabetic wound healing (**Figure** **23**A). The FEP@exo dressing exhibited multifunctional properties, including efficient antibacterial properties towards multidrug‐resistant bacteria, fast hemostatic ability, self‐healing behavior, and tissue‐adhesive and good UV‐shielding performance. Importantly, the in vivo results from a diabetic full‐thickness cutaneous wound model showed that FEP@exo dressing accelerated wound healing (Figure 23A). In a similar study, an injectable multifunctional hydrogel (FEMI) was developed for diabetic wound repair. The FEMI hydrogel was fabricated through a Schiff‐based reaction between *ε*‐poly lysine (EPL) coated MnO_2_ nanosheets (EM) with insulin‐loaded self‐assembled aldehyde Pluronic F127 (FCHO) micelles (Figure 23B).^[^ ^323^ ^]^ Due to the antibacterial characteristics of MnO_2_ nanoenzyme and the therapeutic activity of insulin, the FEMI hydrogel effectively inhibited multidrug‐resistant bacteria strains and controlled glucose levels. The in vivo results confirmed that the FEMI hydrogel could considerably accelerate diabetic wound healing (Figure 23B) due to its multifunctionality such as eradicating MRSA infection, attenuating hyperglycemia, ameliorating oxidative stress, and continuously supplying O_2_.

Generally, it has been established that delivering specific biomolecules using a combination of nanomaterials and macroscopic scaffolds (e.g., hydrogels and nanofibers) can produce surprising results in terms of re‐epithelialization, collagen fiber deposition, tissue regeneration, and ultimately a faster wound closure in chronic diabetic ulcers. Furthermore, innovative nanomaterials capable of regulating all phases of wound healing, incorporating the antibacterial properties, possessing self‐healing property, excellent mechanical properties, and wound dressing adhesive ability, will improve performance in clinical applications. Therefore, this research direction is an important future scope for researchers working in this area. The antibacterial nanomaterial‐encapsulated scaffolds have proven to be potentially efficient dressing materials for treating wound infections in diabetic patients. Thus, the use of various novel functional nanomaterials in combination with a macroscopic scaffold can be a point of differentiation between conventional and modern wound‐healing therapies, and it is certainly worth further research.

## 5 Conclusion and Future Perspectives

### 5.1 Nanomaterials for Diabetic Sensor Fabrication

**Figure 24:**
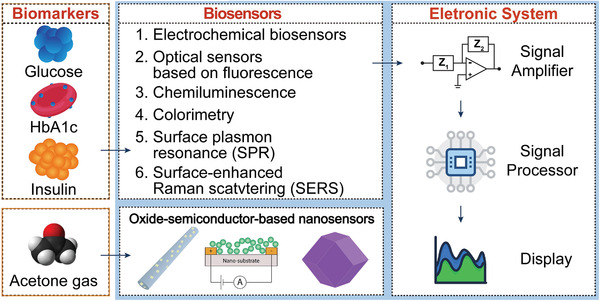
Biosensors for diabetes biomarker detection.

Early diabetic diagnosis can provide guidance for individuals who need interventions such as lifestyle management or pharmacological therapies to prevent dysglycemia or even the onset of disease‐related complications. Biosensors have been extensively studied for detecting diabetes‐related biomarkers including plasma glucose, glycated hemoglobin (HbA1c), insulin, and acetone gas in human breath. The rapid development in the field of nanotechnology brings new opportunities for diabetic biosensors. To obtain high sensitivity and fast response rate, biosensing materials should have large specific surface areas and high accessibility for target molecules to the materials surface by diffusion (**Figure** **24**). Consequently, nanomaterials have unique advantages due to the large specific surface areas and high porosity of their aggregates. For example, the aforementioned studies have demonstrated the importance of porosity and specific surface area of semiconductor NPs for the catalytic reduction/oxidation of diabetes‐related acetone gas. Despite the improvement in acetone gas sensing efficiency, developing ultrafast response for high‐accuracy detection of trace acetone gas in human exhaled breath for clinical diabetes diagnosis remains a challenge, mainly due to the complex component and non‐specificity of catalysis occurring at the metal oxide surface. In contrast, current nanosensors for glucose or HbA1c detection seem to be superior. The problem of acetone sensors perceiving non‐specificity can be addressed by modifying the nanosensors with glucose oxidase or phenylboronic acid. However, further efforts are needed towards the point‐of‐care diagnosis, since current diabetic sensors mostly rely on special instruments to analyze data. Fortunately, progress has been made in this area, and we anticipate that the color‐changing contact lenses and paper‐based glucose and acetone gas test strips will be one of the most promising directions for diabetic diagnosis.

### 5.2 Nanomaterials as Drug Carriers

**Figure 25:**
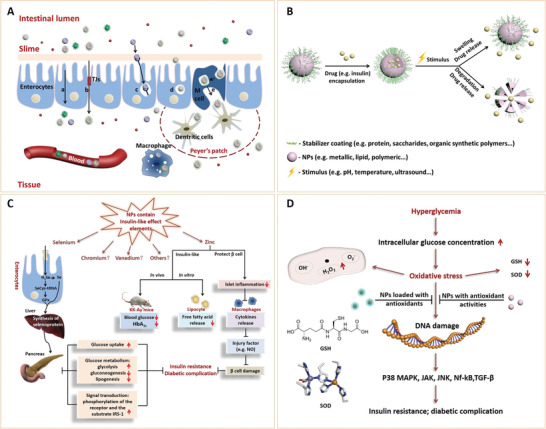
A) Schematic diagram of nanocarrier transport. Five potential pathways for the transepithelial transport of nanocarriers: a) transcellular pathway; b) paracellular pathway through TJs located between epithelial cells; c) receptor‐mediated endocytosis; d) adsorptive‐mediated endocytosis; and e) M cell‐associated pathway across the epithelium. B) Mechanisms of stimulus‐responsive drug release: pH, temperature, redox potential, enzyme, light, or ultrasound stimulation changes nanocarrier structure (swelling or degradation), which leads to drug release. C) The potential antidiabetic mechanisms of NPs containing elements with insulin‐like activities (selenium, zinc, chromium, vanadium, and so forth). (↑): activation or increase, (↓): inhibition or decrease, (┤): inhibition, (?): not clear, HbA_1c_: Hemoglobin A_1c_, IRS‐1: insulin receptor substrate‐1, NPs: nanoparticles, SeCys: selenocysteine, GPx: glutathione peroxidase, and NO: nitric oxide. D) Inhibition of diabetes and the complications through the antioxidant activity of NPs. (↑): activation or increase, (↓): inhibition or decrease, (┤): inhibition, P38 MAPK: P38‐mitogen‐activated protein kinase, JAK: Janus tyrosine kinase, JNK: Jun N‐terminal kinase, Nf‐ĸB: nuclear factor‐ĸB, NPs: nanoparticles, and TGF‐*β*: transforming growth factor beta. Signal transduction pathways mediated by P38 MAPK, JAK, JNK, JUN, Nf‐kB and TGF‐*β* promote the occurrence and/or development of diabetic complications.

Effective nanoparticles for delivering antidiabetic drugs and various extracts from antidiabetic natural substances have been developed. The antidiabetic substances are loaded onto NPs for protection against digestion by the GI enzymes and to enhance their bioavailability. Due to their good tolerability, sufficient physical, and biological stability, NPs can deliver drugs across the intestinal epithelial barrier in multiple ways,^[^ ^189^ ^]^ including transcellular or paracellular diffusion, receptor or adsorptive‐mediated endocytosis, and microfold cell (M cell) antigen sampling (**Figure** **25**A). In addition, employment of stimulus‐response systems such as varying pH values, differences in temperature, or magnetic systems can impel the release of drugs at the target point for further therapy (Figure 25B).

Nanoparticles can be effectively transported through the cells via active transcellular transport.^[^ ^324^ ^]^ For example, insulin‐loaded HA NPs were transported across Caco‐2 cell mainly via the transcellular pathway, and HA NPs significantly enhanced insulin transport through the duodenum and ileum.^[^ ^325^ ^]^

Nanoparticles have been shown to expedite paracellular transport of macromolecules by opening the TJs between intestinal epithelial cells.^[^ ^326^ ^]^ Su et al.^[^ ^156^ ^]^ found that chitosan poly(*γ*‐glutamic acid)‐diethylene triamine pentaacetic acid (CS/*γ*PGA‐DTPA) nanoparticles (prepared by mixing cationic CS with anionic *γ*‐PGA‐DTPA conjugate) promoted insulin absorption throughout the entire small intestine via paracellular transport by chelating Ca^2+^ from AJs, resulting in TJ disruption.

Nanoparticles prepared with bound ligands for specialized receptors expressed on epithelial cell surfaces can be transcytosed across epithelial barriers.^[^ ^189^ ^]^ Liu et al.^[^ ^327^ ^]^ synthesized NPs by coating the insulin‐loaded poly(lactide‐co‐glycolide)‐monomethoxy‐poly(polyethylene glycol) micelle cores with FQS (targeting ligand FQSIYPpIK) modified trimethyl CS chloride for oral insulin delivery. The results revealed that the NPs improved the drug release profile and enzymatic stability, and displayed observably accelerated intracellular uptake by the ligand‐receptor‐mediated endocytosis. Moreover, both transcellular and paracellular pathways demonstrated improved transport of the targeted NPs through the Caco‐2 cells. Additionally, adsorptive‐mediated endocytosis, which is based on material interaction with the cellular membrane, has also been used to describe cellular entry of NPs.^[^ ^328^ ^]^ Indeed, many drug delivery strategies targeting the brain have focused on adsorptive‐mediated transcytosis, which is triggered by electrostatic interactions between cationic molecules and anionic microdomains on the cytoplasm membrane of brain capillary endothelial cells.^[^ ^329^ ^]^ Therefore, cationic charged nanoparticles can exploit this pathway for transport across epithelial barriers.

Microfold (M) cells, which are specialized for antigen sampling, are attractive targets for NP delivery.^[^ ^330^ ^]^ The cells are specialized lymphoid cells present in the gut‐associated (intestinal Peyer's Patches) and mucosa‐associated lymphoid tissue (e.g., Nasal Associated Lymphoid Tissue).^[^ ^331^ ^]^ Microfold cells have reduced protease activity and glycocalyx properties, which are both beneficial to NP transcytosis.^[^ ^332^ ^]^ However, transport of nanoparticles via M cells may be limited. For example, because M cells occupy just 10% of the follicle‐associated epithelium in mouse and human, and NPs do not have M‐cell specificity, nanoparticles absorption by M cells could be restricted.^[^ ^333^ ^]^ Moreover, NPs transported by M cells can be captured by macrophages and dendritic cells. These findings indicated that NPs have limited entry into the bloodstream and provide an opportunity for oral vaccination.^[^ ^330^ ^]^

### 5.3 Nanomaterials with Insulin‐Like Activities

Because the elements in some NPs have an insulin‐like effect, they have significant antidiabetic properties (Figure 25C). In previous studies, the element Se was found to act as an insulin mimic and to have antidiabetic properties. Although its antidiabetic mechanism remains unclear, Se is incorporated into the synthesis of many antioxidant proteins that serve as scavengers of ROS, such as glutathione peroxidase.^[^ ^334^ ^]^ Since oxidative stress is a major cause of diabetes, Se's antioxidant property could be one of its key antidiabetic mechanisms. Another conceivable mechanism is the influence of Se on glucose metabolism.^[^ ^335^ ^]^ Like insulin, Se can regulate key enzymes in sugar and lipid metabolism. The levels of tyrosine phosphorylation of two signaling molecules in the insulin signaling network, including insulin receptor and IRS‐1 (insulin receptor substrate‐1), are increased in the presence of selenium. Thus, administering Se supplements was deemed to be an effective strategy to prevent and treat diabetes.^[^ ^336^ ^]^ Recently, the antidiabetic property of Se NPs, which are composed of a type of red elemental Se in a colloidal state, has been confirmed.^[^ ^337^ ^]^ Se NPs have attracted extensive attention due to their high bioavailability, biological activity, and low toxicity.

Similarly, zinc supplementation has been shown to augment glycemic control in Type‐I and ‐II diabetes in both animals and humans, but the underlying molecular mechanisms are still being clarified. Zinc has been shown to inhibit free fatty acid release after epinephrine stimulation of adipocytes in vitro and to lower blood glucose and hemoglobin A_1c_ levels in KK‐Ay mice when administered intraperitoneally. Zinc shows insulin‐like effects by interacting with molecules involved in insulin signal transduction and protecting insulin‐producing beta cells from death, resulting in higher plasma insulin level.^[^ ^338^ ^]^

The use of chromium and vanadium in the treatment of Type‐I and ‐II diabetes symptoms has also been proposed. Vanadium appears to be an insulin mimic, whereas chromium (III) apparently can amplify insulin sensitivity.^[^ ^339^ ^]^ These results suggested that chromium and vanadium NPs may also have significant antidiabetic properties, which are yet to be reported probably due to the controversy that surrounds their effects in diabetes therapy. For example, Domingo and Gómez^340^ ^]^ concluded that using vanadium to treat human diabetes was misplaced. Consequently, additional research is needed to determine whether chromium (III) compounds have potential to cure the symptoms of Type‐II diabetes and related conditions.^[^ ^339^ ^]^ In addition, more research is required to determine if chromium and vanadium NPs can be used as carriers for the delivery of antidiabetic drugs, and to test whether they can be used to detect diabetes like some magnetic NPs.

### 5.4 Nanomaterials with Antioxidant Activities

Oxidative stress plays a vital role in the development of diabetes and its complications due to ROS overproduction and/or decreased ROS scavenging by antioxidants like superoxide dismutase and glutathione. Hyperglycemia is thought to induce ROS by causing excessive glucose and lipid oxidation. Reactive oxygen species, including superoxide anion (O_2_^−^), H_2_O_2_, and hydroxyl radical (OH·), are formed by the reduction of molecular oxygen or the oxidation of water.^[^ ^341^ ^]^ Large quantities of ROS can damage cellular lipids, membranes, proteins, and DNA as they are electron deficient, lipophilic and easily move inside the biological membranes. They can also activate signal transduction factors such as P38 mitogen‐activated protein kinase (P38 MAPK), Janus tyrosine kinase (JAK), Jun N‐terminal kinase (JNK), nuclear factor‐ĸB (Nf‐ĸB), and transforming growth factor (TGF)‐*β*, which promote the occurrence and/or development of diabetic complications (Figure 25D).^[^ ^342^ ^]^ Nanomaterials that inhibit or delay the oxidation of a substrate (i.e., antioxidant activity) could be useful in the treatment of both Type‐I and ‐II diabetes. A very enlightening example is that overexpression of antioxidant enzymes in diabetic patients prevented diabetic complications such as retinopathy, nephropathy, and cardiomyopathy.^[^ ^205^ ^]^ To date, an increasing number of NPs with substantial antioxidant properties have been found (**Table** **1**). For example, nano‐ZnO has similar properties to the SOD and catalase, whereas CeO_2_ NPs may eliminate oxidative stress through a hypoglycemic effect and glycogenolysis inhibitory mechanism. Because Se‐containing NPs can eliminate oxidative stress by enhancing glucose transport activity, they have a potential synergistic effect with CeO_2_ NPs. In addition to Zn‐, Ce‐, and Se‐based antioxidant NPs, gadofullerene nanomaterials, a representative of carbon‐based nanomaterials, have demonstrated good ROS scavenging capabilities.

**Table 1:** Nanoparticles with antioxidant activities (ABTS, 2,2‐azino‐bis‐3‐ethylbenzothiazoline‐6‐sulphonic acid; DPPH, 2,2‐diphenyl‐1‐picrylhydrazyl; MDA, malondialdehyde; ROS, reactive oxygen species; SOD, superoxide dismutase)

Nanoparticle	Size [nm]	Antioxidant activity assay	Refs.
Selenium	–	In vivo antioxidant activity	^[^ ^206^, ^213^ ^]^
Cerium oxide	–	In vivo antioxidant activity	^[^ ^213^ ^]^
Levan‐coated cerium oxide	5	DPPH and ROS scavenging activities	^[^ ^343^ ^]^
Gadofullerene	138 ± 2	In vivo antioxidant activity	^[^ ^219^ ^]^
Zinc oxide	10 nm (synthesized in aqueous phase); 40 nm (synthesized in organic phase)	Scavenging activities (DPPH, ABTS, OH^•^, O_2_^−•^); Melt chelating	^[^ ^344^ ^]^
Copper	42–90	DPPH scavenging activity	^[^ ^345^ ^]^
Silver	9–46	DPPH scavenging activity	^[^ ^346^ ^]^
Silver (using *Garcinia indica* extract)	5–30	Scavenging activities (DPPH, OH^•^, NO); Reducing power activity	^[^ ^347^ ^]^
Bismuth	20–120	DPPH scavenging activity; Reducing power activity	^[^ ^348^ ^]^
Gold	–	DPPH scavenging activity	^[^ ^349^ ^]^
Melanin	–	In vivo antioxidant activity	^[^ ^350^ ^]^
Poly(caprolactone)	185	Scavenging activities (DPPH, O_2_^−•^); Fe^2+^ chelating	^[^ ^351^ ^]^
Bovine serum albumin	175 ± 6	Hypochlorous acid scavenging activity	^[^ ^352^ ^]^
Silicon dioxide	50	Rotary oxygen bomb test	^[^ ^353^ ^]^

Overall, due to their intrinsic antioxidant activities and their ability to deliver other antioxidants, the aforementioned NPs have shown great potential in the prevention and therapy of diabetes and related complications. However, more research into the bio‐toxicity of these NPs is needed. Bio‐security might be the major problem that is impeding the practical application of these functional nanomaterials.

### 5.5 Nanomaterials against Human Islet Amyloid Polypeptide (hIAPP) Aggregation

Human islet amyloid polypeptide (hIAPP) is a 37‐residue peptide that coexpresses and cosecretes with insulin in pancreatic *β* cells.^[^ ^354^ ^]^ However, it was named for its ability to aggregate into pancreatic islet amyloid deposits, which are seen frequently in people with type II diabetes.^[^ ^355^ ^]^ Existing evidence shows that hIAPP oligomers and mature fibrils are both toxic and may contribute to *β*‐cell loss in type II diabetes.^[^ ^184^, ^185^, ^186^ ^]^ Hence, inhibiting hIAPP aggregation has been considered as one of the primary strategies to treat Type‐II diabetes.^[^ ^356^ ^]^

**Figure 26:**
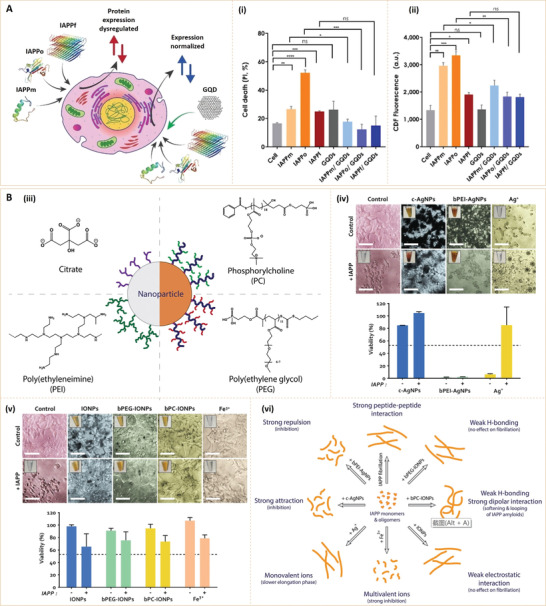
A) Viability, morphology and ROS generation of *β*TC6 pancreatic *β*‐cells exposed to the IAPP species with or without GQDs (i). Proteomics analysis of IAPP‐ and GQD‐treated pancreatic *β*‐cells. Expression of 29 proteins was altered by the different forms of IAPP treatments (ii). Reproduced with permission.^[^ ^358^ ^]^ Copyright 2019, Tsinghua University Press and Springer. B) Schematic of the metal NPs: a silver nanoparticle (gray) coated with citrate or branched polyethyleneimine (c‐AgNP, bPEI‐AgNP), and an iron oxide NP (brown) grafted with brushed phosphorylcholine or polyethylene glycol (bPC‐IONP, bPEG‐IONP) (iii). The differential effects of silver and iron oxide NPs (Ag NPs and IONPs) on the mesoscopic properties of human islet amyloid polypeptide (IAPP) aggregation associated with Type‐II diabetes (iv). Viability of HEK293 cells treated with bPEG polymer, bPC polymer, citrate salt and bPEI after 24 h (v). Viability of HEK293 cells treated with IAPP, AgNPs, Ag^+^, IAPP, IONPs, and Fe^3+^ after 24 h (vi). Reproduced with permission.^[^ ^362^ ^]^ Copyright 2017, The Royal Society of Chemistry.

In recent studies, nanoparticles have shown potential in inhibiting hIAPP aggregation. Mo et al.^[^ ^356^ ^]^ proposed that hydroxylated single‐walled carbon nanotubes (SWCNT‐OH) can obstruct hIAPP fibrillation. They performed explicit solvent replica exchange molecular dynamics (REMD) simulations to investigate the influence of SWCNT‐OHs on the first step of hIAPP aggregation: dimerization. The results demonstrated that SWCNT‐OHs can dramatically inhibit interpeptide *β*‐sheet formation and completely suppress the previously reported *β*‐hairpin amyloidogenic precursor of hIAPP. Furthermore, detailed analyses of hIAPP‐SWCNT‐OH interactions indicated that hydrogen bonding, van der Waals, and *π*‐stacking interactions between hIAPP and SWCNT‐OH significantly weaken the inter‐ and intrapeptide interactions that are crucial for *β*‐sheet formation. Indeed, several carbon nano‐materials have been found to interfere with protein misfolding and aggregation. Guo et al.^[^ ^357^ ^]^ used molecular dynamics simulations to explore the effects of three different carbon nanomaterials including graphene, carbon nanotube, and C_60_ on the aggregation behavior of islet amyloid polypeptide fragment 22–28 (IAPP_22–28_). The results showed that these nanomaterials can prevent *β*‐sheet formation to varying degrees and affect the aggregation of IAPP_22–28_. Furthermore, Faridi et al.^[^ ^358^ ^]^ examined how proteomes of *β*TC6, a pancreatic *β*‐cell line, changed upon exposure to monomeric, oligomeric, and fibrillar IAPP, and detailed cellular protein expression rescued by graphene quantum dots (GQDs), an IAPP inhibitor. The liquid chromatography tandem‐mass spectrometry, fluorescence quenching, helium ion microscopy, cytotoxicity, and discreet molecular dynamics simulations data revealed that GQDs have a remarkable capacity for regulating aberrant protein expression through H‐bonding and hydrophobic interactions, pointing to nanomedicine as a new frontier against human amyloid diseases (**Figure** **26**A). In addition, recent studies have reported that certain NPs (e.g., gold,^[^ ^359^ ^]^ polymeric NPs, ^[^ ^360^ ^]^ dendrimer,^[^ ^361^ ^]^ etc.) have distinct effects on inhibiting IAPP aggregation. Wang et al.^[^ ^362^ ^]^ demonstrated that both citrate‐ and branched polyethyleneimine‐coated Ag NPs (c‐AgNPs, bPEI‐AgNPs) inhibited IAPP aggregation at 500 µg mL^−1^, likely through electrostatic attraction and sequestering of IAPP monomers from fibrillation (Figure 26B).

Taken together, different types of NPs showed potential in the treatment and prevention of diabetes due to their capacities to establish connections like hydrogen bonding, hydrophobic interaction, and *π*‐stacking with the amyloid peptide, thus disrupt the latter's amyloid aggregation.

### 5.6 Perspectives

Early diagnosis, management, and the treatment of diabetes and its complications have become an ineluctable challenge for all researchers and healthcare personnel. Fortunately, recent development in nanomedicine has presented new opportunities for diabetes‐related medical requirements. In this review, we provide an overview of the recently developed nanomaterials for both diabetic biomarker detection and diabetes treatment. Despite the numerous advantages of nanomaterial‐mediated diabetic theranosis, there are still a number of problems that need to be addressed.

The efficient, fast, accurate, and convenient detection of diabetic biomarkers represent the key issues for future development in diabetes diagnosis. This is a relatively easy task for researchers since the use of biosensors for diabetes diagnosis is commonly based on in vitro or ex vivo detection. In comparison to detecting diabetic biomarkers in blood samples (e.g., glucose and A1c), monitoring of acetone gas in human exhaled breath is a particularly appealing method because it is noninvasive. Accordingly, acetone nanosensors with ultrafast response and high accuracy are currently being developed. Future research should focus on the development of commercially available semiconductor oxide acetone sensors and adapting the practical aspects of clinical action testing.

Nanomaterials for diabetes therapy, unlike diabetic diagnosis, inevitably involve in vivo assays. Although numerous studies show that NPs have antidiabetic properties, there are still a number of issues to be investigated concerning the use of nanoparticles in diabetes therapy, such as the doses of NPs that the patients can safely and effectively take, the possible antidiabetic mechanisms of NPs, and the epigenetic effects of NPs. Furthermore, some NPs, such as superparamagnetic NPs, can be recognized and phagocytosed by cells present in the reticulocyte‐endothelial system of the liver, spleen, and lymph nodes. Accumulation in these cells limits their therapeutic activity and may lead to toxicity, a possibility that needs comprehensive scientific evaluation. Importantly, how may these endothelial phagocytosis systems be avoided so that NPs can accurately reach their target? This is a common concern in nanomedicine that has to be solved.

As previously stated, the mechanisms underlying NP toxicity and other multiple roles in diabetes prevention and therapy are extremely essential from both a fundamental and application point of view. To lower the toxicity of NPs, the raw materials and processes involved in their synthesis must be optimized. Minimizing the use of toxic chemical reagents or choosing to use natural substances in the synthesis process may improve biosecurity. Furthermore, studies on the in vivo regulation and control mechanisms of antidiabetic elements or oxides are vital for the application of their nanoparticles in diabetes. The greatest effect of nanoparticles will be ensured by developing innovative strategies to effectively target nanoparticles towards damaged tissues in diabetic patients. Since an ideal NP drug delivery system should be able to reach, recognize, bind, and deliver its load to sick tissues while minimizing or avoiding drug‐induced damage to healthy tissues, modification of NPs with specific targeting ligands is likely to be the most simple and efficient strategy.

Overall, nanoparticles have shown great potential in the area of diabetes diagnosis and treatment. Although we have witnessed many accomplishments of functional nanosystems in the detection, treatment, and prevention of diabetes and its complications, this is still a developing research area in need of multidiscipline cooperation, combining chemistry, material science, biology, medicine, and electronics and computer science. Further efforts towards the discovery of new diabetic biomarkers, the development of advanced sensing mechanisms and point of care detection devices, the design of high‐efficacy drug nanocarriers and functional nanosystems with unique antidiabetic properties, and the reduction of biotoxicity of current nanomaterials are fundamental. We hope the concepts presented in this review article, including the existing challenges and future growing trends, will inspire researchers to develop unique nanomaterials with novel functions and properties for diabetes diagnosis and therapy.

## Conflict of Interest

The authors declare no conflict of interest.
